# Allergen fragrance molecules: a potential relief for COVID-19

**DOI:** 10.1186/s12906-021-03214-4

**Published:** 2021-01-21

**Authors:** Aslı Deniz Aydın, Faruk Altınel, Hüseyin Erdoğmuş, Çağdaş Devrim Son

**Affiliations:** 1 EPS FRAGRANCES (Erdogmus Parfum Sanayi), Mithatpaşa Mah, Gökşin Sok, No: 33A, 34075, Kemerburgaz, İstanbul, Türkiye; 2 Başkent Üniversitesi, İzmir Zübeyde Hanım Uygulama Merkezi, Caher Dudayev Bulvarı no: 175 Bostanlı Karşıyaka, İzmir, Türkiye; 3 Department of Biological Sciences, Middle East Technical University, Universiteler Mah. Dumlupinar, Blv. No: 1, 06800 Çankaya, Ankara, Türkiye

**Keywords:** COVID-19, SARS-CoV-2, Coronavirus, Docking, Fragrance allergen molecules, Anti-viral fragrance molecules

## Abstract

**Background:**

The latest coronavirus SARS-CoV-2, discovered in China and rapidly spread Worldwide. COVID-19 affected millions of people and killed hundreds of thousands worldwide. There are many ongoing studies investigating drug(s) suitable for preventing and/or treating this pandemic; however, there are no specific drugs or vaccines available to treat or prevent SARS-CoV-2 as of today.

**Methods:**

Fifty-eight fragrance materials, which are classified as allergen fragrance molecules, were selected and used in this study. Docking simulations were carried out using four functional proteins; the Covid19 Main Protase (MPro), Receptor binding domain (RBD) of spike protein, Nucleocapsid, and host Bromodomain protein (BRD2), as target macromolecules. Three different software, AutoDock, AutoDock Vina (Vina), and Molegro Virtual Docker (MVD), running a total of four different docking protocol with optimized energy functions were used. Results were compared with the five molecules reported in the literature as potential drugs against COVID-19. Virtual screening was carried out using Vina, molecules satisfying our cut-off (− 6.5 kcal/mol) binding affinity was confirmed by MVD. Selected molecules were analyzed using the flexible docking protocol of Vina and AutoDock default settings.

**Results:**

Ten out of 58 allergen fragrance molecules were selected for further docking studies. MPro and BRD2 are potential targets for the tested allergen fragrance molecules, while RBD and Nucleocapsid showed weak binding energies. According to AutoDock results, three molecules, Benzyl Cinnamate, Dihydroambrettolide, and Galaxolide, had good binding affinities to BRD2. While Dihydroambrettolide and Galaxolide showed the potential to bind to MPro, Sclareol and Vertofix had the best calculated binding affinities to this target. When the flexible docking results analyzed, all the molecules tested had better calculated binding affinities as expected. Benzyl Benzoate and Benzyl Salicylate showed good binding affinities to BRD2. In the case of MPro, Sclareol had the lowest binding affinity among all the tested allergen fragrance molecules.

**Conclusion:**

Allergen fragrance molecules are readily available, cost-efficient, and shown to be safe for human use. Results showed that several of these molecules had comparable binding affinities as the potential drug molecules reported in the literature to target proteins. Thus, these allergen molecules at correct doses could have significant health benefits.

**Supplementary Information:**

The online version contains supplementary material available at 10.1186/s12906-021-03214-4.

## Background

Coronaviruses (CoVs) belong to the family Coronaviridae, which are enveloped, single-stranded, positive-sense RNA viruses. These viruses generally contain large (~ 20 nm), surface projections called “spikes,” which in electron micrographs create an image reminiscent of the solar corona, thus giving the name to the family. CoVs commonly cause respiratory problems but can also disrupt the digestive system or lead to systemic problems in mammals, birds, and reptiles. In humans, they can cause very severe respiratory diseases such as SARS-CoV in 2002 and Middle East respiratory syndrome coronavirus (MERS-CoV) in 2012 [1]. The latest coronavirus SARS-CoV-2, discovered in China, affected millions of people and killed hundreds of thousands worldwide. The World Health Organization (WHO) announced “COVID-19” as the name of this new disease caused by SARS-CoV-2 [2]. The ongoing SARS-CoV-2 threat that emerged in China has rapidly spread Worldwide and continuing to spread. Thus, many efforts directed to investigate drug(s) suitable for preventing and/or treating human SARS-CoV-2.

As of the publication date of our study, there are no specific drugs or vaccines available to treat or prevent SARS-CoV-2. Several countries implemented drugs based on symptom-based therapies [3–5] to avoid further complications and organ damage [6]. There are various preliminary studies for the treatment of SARS-CoV-2 infected patients. Anti-retroviral drugs such as remdesivir, lopinavir, ritonavir, oseltamivir used in individual healing trials, or animal experiments. From the Wuhan Institute of Virology, Wang et al. investigated some of the FDA approved drugs and found that remdesivir and chloroquine could effectively inhibit the virus in cell-based assay with EC_50_ of 0.77 and 1.13 μM, respectively [7]. Similar studies reported that a combination of protease inhibitor lopinavir/ritonavir could be used for the treatment of SARS-CoV-2 [8].

Other anti-viral treatments include; nucleoside analogs, neuraminidase inhibitors, umifenovir (arbidol), tenofovir disoproxil (TDF), and lamivudine (3TC) [9]. According to binding free energy calculations, using the molecular mechanics, Xu et al. indicated that among four tested drugs (nelfinavir, pitavastatin, perampanel, and praziquantel) nelfinavir was identified as the most potent inhibitor against COVID-19 MPro [10]. Besides, alternative traditional Chinese medicine implementations have been reported [3, 4, 11, 12]. Although results from these preliminary studies remain unapproved for therapeutic use in clinical settings, they are still precious for drug studies against the current pandemic. To speed up possible clinical trials and drug discovery against SARS-CoV-2, many compounds that are being used as drugs or supplements for humans are started to be tested as potential lead molecules. Molecules that are going to be implemented as anti-viral treatment and protection have several requirements: First of all, stock of the drug must be sufficient and readily available; the safety of the procedure, treatment should be tolerated by the patients, and finally, the cost should be as low as possible. In this respect, we investigate selected fragrance materials, which are classified as allergen fragrance molecules according to the 7th amendment of the 76/768/EEC Directive (European Economic Community Cosmetics Directive), as mentioned at Scientific Committee on Consumer Safety (SCCS) [13].

Fragrance substances are mixtures of natural essential oils and synthetic organic odorous compounds with characteristics, usually pleasant odors. They are used in perfumes and scented cosmetic products, detergents, soaps, fabric softeners, air care, incense, and other household products where fragrance may be used to mask unpleasant odors from raw materials and give a pleasant smell [14–18]. These substances are also used in aromatherapy and other products as topical medicaments for their antiseptic, antibacterial, antifungal, and anti-viral properties.

Throughout the evolutionary history of life on Earth, essential oils in plants and trees have been theorized to evolve with viruses, bacteria, and fungi to protect plants and trees from viral, bacterial, and fungal infections. Many studies on the anti-viral behavior of plants showed the defense mechanism is based on essential oils [19, 20]. Thus, fragrance materials under various categories have been tested for anti-viral activities [21]. More importantly, some essential oils such as *Laurus nobilis* Oil, *Juniperus oxycedrus* Oil and *Theileria orientalis* Oil shown to be effective against the SARS-CoV-1 virus [22]. When the major constituents of these oils analyzed, we identified 16 molecules (β-Pinene, Eugenol, Cinnamaldehyde, α-Terpinene, α-Terpineol, Anethole, β-Caryophyllene, Camphor, Citral, Geranial, Geraniol, Limonene, Linalool, Linalyl Acetate, Menthol, Terpinolene) that belongs to fragrance allergens subgroup.

Fragrance contact allergy has long been recognized. Contact allergy to fragrance ingredients may develop following skin contact with a sufficient amount of these substances, often through the use of cosmetic products. Contact allergy is an altered specific reactivity in the immune system, which entails recognition of the fragrance allergens in question by immune cells, indicating an interaction with surface proteins such as receptors. The 58 molecules used in this study are classified as Fragrance allergens in cosmetic products by Scientific Committee on Consumer Safety (SCCS), where only 26 of them are officially accepted as allergens according to the DIRECTIVE 2003/15/EC OF THE EUROPEAN PARLIAMENT AND OF THE COUNCIL of 27 February 2003 amending Council Directive 76/768/EEC on the approximation of the laws of the Member States relating to cosmetic products. There are no preventions for these products’ human use as long as the labels indicate the contents, and none showed known allergic reactions upon inhalation. We hypothesize that these allergen molecules at correct doses could have significant health benefits, and studies with these molecules could help us identify potential target molecules for drug development.

Considering the potential anti-viral effects of these molecules and the urgency of an effective drug that can prevent and/or treat the pandemic, we carried out the repurposing approach by screening fragrance molecules using docking simulations to identify lead molecules against COVID-19 [23]. For this purpose, high resolution crystal structures of viral proteins and proteins that could be important for viral infection were searched. Four functional proteins that have structure information submitted to Research Collaboratory for Structural Bioinformatics Protein Data Bank (RCSB-PDB) with non-covalent ligand co-crystallized and reported as essential for COVID-19 spread were used as target proteins. These proteins include the Covid19 Main Protase (MPro), Receptor binding domain (RBD) of spike protein, Nucleocapsid, or the N-protein and Host Bromodomain protein (BRD2).

The first protein we used in this study is the target protein used in many recent studies, the Main protease, which is also named as chymotrypsin-like protease [24–26]. This protease can cleave many sites in the polyproteins and generate nonstructural proteins (nsps) that play a role in the assembly of replicase-transcriptase complex (RTC). Inhibition of this protease suggested as a potential drug that can prevent the spread of Covid19. Several groups resolved the crystal structure of this protein, and RCSB-PDB contains many entries for it. In our studies, we used the structure of COVID-19 main protease bound to potent broad-spectrum non-covalent inhibitor X77 (6W63) [27].

The next protein we picked is part of the surface spike glycoprotein, which consists of two subunits (S1-S2) and is a heterodimer. The RBD located on the head of S1 binds with the cellular Receptor angiotensin-converting enzyme 2 (ACE2), initiating the membrane fusion of the virus and host cell. Thus, blocking this interaction could slow down or inhibit the infection. The RCSB-PDB structure 6VW1, containing the RBD and part of ACE2, was downloaded and prepared for docking studies [28].

The third protein in our study is the Nucleocapsid (N) protein of COVID-19, which has nearly 90% amino acid sequence identity with SARS-CoV nucleocapsid protein. The N protein of COVID-19 may play an essential role in suppressing RNA interference (RNAi), which could overcome the host defense. Thus, blocking this protein might help the host defense against COVID-19 [29]. The crystal structure of the RNA binding domain of nucleocapsid phosphoprotein from SARS-CoV-2 was downloaded from RCSB-PDB (6VYO) [30].

Unlike the first three proteins, the fourth protein in our study is a host protein, which is a member of the bromodomain extra terminal protein family. These proteins are known to regulate the expression of ~ 1450 genes. Early reports from the cell mapping initiative at Quantitative Biosciences Institute Coronavirus Research Group indicate that BRD2, a member of the bromodomain protein family, may interact with SARS-CoV-2 envelope proteins. The bromodomain proteins recognize and bind to acetylated histones and play a critical role in the host’s hype-immune response. COVID-19 virus protein E mimics acetylated histones and could bind to the same site on BRD-2 [31]. Inhibitors targeting the bromodomain proteins are already being used in the clinic, such as Resverlogix’s apabetalone, in phase 3 trials for cancer and phase 1 for pulmonary arterial hypertension; AbbVie’s ABBV-744, in phase 1 for cancer; and Constellation Pharmaceuticals’ CPI-0610, in phase 2 for cancer, suggesting that BRD2 inhibitors can be used as drug candidates. Similarly, it has been suggested that BRD2 inhibitors can potentially block where COVID-19 envelop protein E binds and could be used as drug targets for COVID-19. In our study, we used the crystal structure (5UEW) of BRD2 obtained from RCSB-PDB [32].

The allergen fragrance molecules used in docking simulations are summarized in Table 1.

**Table 1:** Allergen Fragrance Materials used in this study

Name	CAS number
3-PROPYLIDENE PHTHALIDE	17369–59-4
ACETYL ISOEUGENOL	93–29-8
ALPHA AMYL CINNAMALDEHYDE	122–40-7
ALPHA AMYLCINNAMYL ALCOHOL	101–85-9
ALPHA DAMASCONE	43052–87-5 / 23726–94-5
ALPHA HEXYL CINNAMALDEHYDE	101–86-0
ALPHA ISOMETHYLIONONE	127–51-5
ALPHA PINENE	80–56-8
ALPHA TERPINENE	99–86-5
ALPHA TERPINEOL	98–55-5
AMYL SALICYLATE	2050-08-0
ANETHOLE	4180-23-8
ANISE ALCOHOL	105–13-5
BENZALDEHYDE	100–52-7
BENZYL ALCOHOL	100–51-6
BENZYL BENZOATE	120–51–4
BENZYL CINNAMATE	103–41-3
BENZYL SALICYLATE	118–58-1
BETA CARYOPHYLLENE	87–44-5
BETA DAMASCENONE	23696–85-7
BETA DAMASCONE	23726–92-3
BETA PINENE	127–91-3
CAMPHOR	76–22-2 / 464–49-3
CARVONE	99–49-0 / 6485-40-1 / 2244-16-8
CINNAMALDEHYDE	104–55-2
CINNAMYL ALCOHOL	104–54-1
CITRAL	5392-40-5
CITRONELLOL	106–22-9 / 1117-61-9 / 7540–51–4
COUMARIN	91–64-5
DELTA DAMASCONE	57378–68-4
DIHYDROAMBRETTOLIDE	109–29-5
DIMETHYLBENZYLCARBINYL ACETATE	151–05-3
EBANOL	67801–20-1
EUGENOL	97–53-0
EUGENYL ACETATE	93–28-7
FARNESOL	4602–84-0
GALAXOLIDE	1222-05-5
GERANIAL	141–27-5
GERANIOL	106–24-1
GERANYL ACETATE	105–87-3
HYDROXYCITRONELLAL	107–75-5
ISO E SUPER	54464–57-2 / 54464–59-4 / 68155–66-8 / 68155–67-9
ISOEUGENOL	97–54-1
LILIAL (BUTYLPHENYL METHYLPROPIONAL)	80–54-6
LIMONENE	138–86-3
LINALOOL	78–70-6
LINALYL ACETATE	115–95-7
LYRAL (HYDROXYISOHEXYL-3-CYCLOHEXENE CARBOXALDEHYDE)	31906–04-4 / 51414–25-6
MAJANTOL	103694–68-4
MENTHOL	1490-04-6 / 89–78-1 / 2216-51-5
METHL-2-OCTYNOATE	111–12-6
METHYL SALICYLATE	119–36-8
SALICYLALDEHYDE	90–02-8
SANTALOL	11031–45-1
SCLAREOL	515–03-7
TERPINOLENE	586–62-9
VANILLIN	121–33-5
VERTOFIX (ALPHA ACETYL CEDRENE)	32388–55-9

## Methods

In this study, molecular interaction of allergen fragrance molecules was investigated with four protein structures (BRD2, Main Protease, Nucleocapsid, and Receptor Binding Domain) commonly used in docking studies in search of a lead compound to treat and/or prevent the spread of COVID-19.

Three-dimensional structures of the allergen fragrance molecules in .sdf format were obtained from the PubChem database, which includes three databases; substance, compound, and bioassay databases [33, 34]. The corresponding CAS numbers for the compounds used in this study were presented in Table 1.

The protein .pdb structures with relative accession numbers 5UEW, 6VW1, 6VYO, and 6W63 were received from the PDB database, which is an archive used worldwide, that includes crystal structures of biological macromolecules [35, 36].

### Virtual screening with Vina

The test compounds were subject to docking by using Vina 1.1.2 [37]. For each target protein, the binding pocket was determined by the position of the crystalized ligand in the corresponding .pdb file. The ligand was removed, and the grid box was generated using MGL-AutoDockTools 1.5.7 grid-box option.

Allergen Fragrance Materials’ .pdbqt files were generated using the MGL-AutoDockTools 1.5.7 from the .pdb data of the molecules obtained from PubChem. Similarly, .pdbqt files of proteins were prepared by removing water and metal atoms and then adding polar hydrogens and Kollman charges via MGL-AutoDockTools.

Default settings of Vina were used, as the scoring matrix in this program is stochastic, and each run uses a random seed position; each molecule was docked at least four times using four different exhaustiveness, including the default value of 8, 12, 15 and 20. Statistical analysis of the results was done using GraphPad Prism 8.0.2. The mean and standard error of the mean (SEM) for each compound reported in the results section and compounds with mean binding affinities ≤ − 6.5 kcal/mol were picked for further docking studies.

### Virtual screening with Molegro virtual Docker

After the selection of compounds that had a strong interaction with target proteins using Vina, further confirmation was performed with Molegro Virtual Docker 7 (MVD) [38]. Target protein .pdb files were first imported into the program and prepared for docking using the preparation tab. Later the .pdb file for each allergen fragrance molecule was introduced to the workspace. Using the “detect cavity option,” the possible binding pocket(s) on the target proteins were identified and confirmed the match with the original ligand position in the .pdb file. Following this step Docking wizard option was executed with the MolDock scoring function and default settings. Resulting poses were sorted according to the re-rank score.

### Flexible docking

Following the virtual screening, top 10 selected allergen fragrance molecules and five molecules; Artemisinin (CAS number 63968–64-9) that occurs naturally in *Artemisia annua*, Favipiravir (CAS number 259793–96-9), Hydroxychloroquine (CAS number 118–42-3), Nigellidine (CAS number 120993–86-4) which occurs naturally in *Nigella sativa* seeds and Remdevisivir (CAS number 1809249–37-3), presented in the literature with a potential to be a drug against COVID-19 were used in flexible docking studies. Both Vina and MVD keeps target protein residues fixed while using flexible ligand options during docking. To achieve docking with a higher degree of freedom, binding pocket residues of target proteins allowed to be flexible. For this purpose, flexible and rigid parts of the target protein were generated using MGL-AutoDockTools 1.5.7. Amino acids that were identified as contact residues in crystal structures picked as flexible sites. Generated flexible and rigid .pdbqt files for BRD2 and MPro proteins were used in Vina. When the residues of the target proteins allowed to be flexible, the search space increase significantly, leading a much longer competition time; thus, flexible docking was only applied for selected allergen fragrance molecules that had good binding affinities during the virtual screening.

### AutoDock

Besides the flexible docking option of Vina, AutoDock 4.2.6. with a genetic algorithm was run 10 times for selected compounds using the default settings (population size 150, Maximum number of evals 2,500,000 and maximum number of generations 27,000) to analyze the docking poses and binding energies. The amino acids of the target proteins binding to the ligands were visualized by MGL-AutoDockTools 1.5.7.

## Results

### Virtual screening

**Fig. 1:**
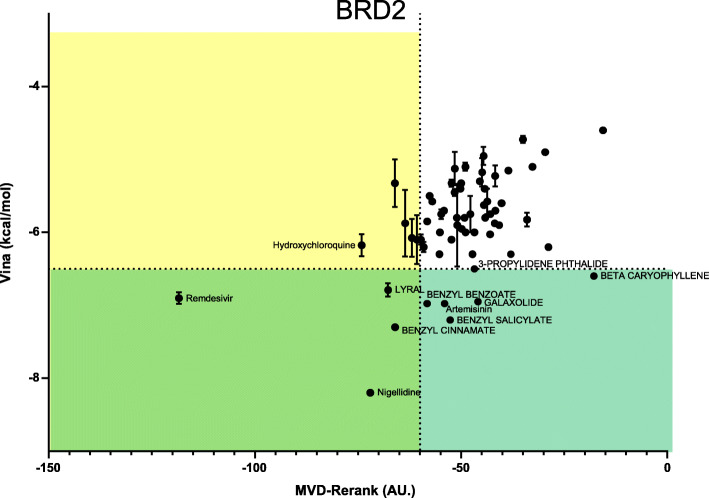
XY-scatter plot for Docking studies using BRD2 as a target protein: Vina and MVD Rerank scores plotted as XY-scatter plot. Regions below − 6.5 kcal/mol and − 60 AU. were marked. The intersect quadrant (green) represents the molecules that satisfy both cut-offs

**Fig. 2:**
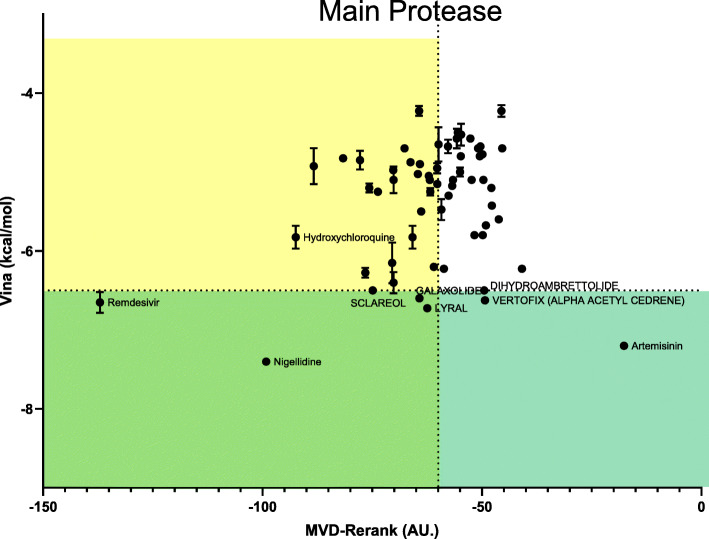
XY-scatter plot for Docking studies using Main Protease as the target protein: Vina and MVD Rerank scores plotted as XY-scatter plot. Regions below − 6.5 kcal/mol and − 60 AU. were marked. The intersect quadrant (green) represents the molecules that satisfy both cut-offs

**Fig. 3:**
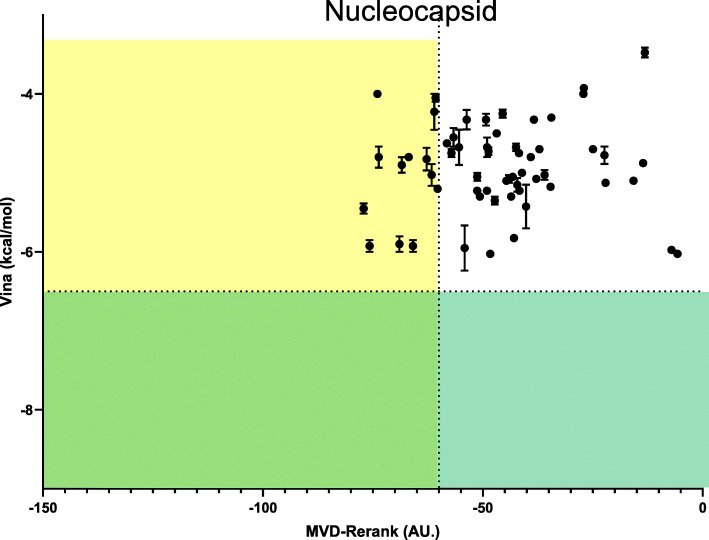
XY-scatter plot for Docking studies using Nucleocapsid as the target protein: Vina and MVD Rerank scores plotted as XY-scatter plot. Regions below − 6.5 kcal/mol and − 60 AU. were marked. The intersect quadrant (green) represents the molecules that satisfy both cut-offs

**Fig. 4:**
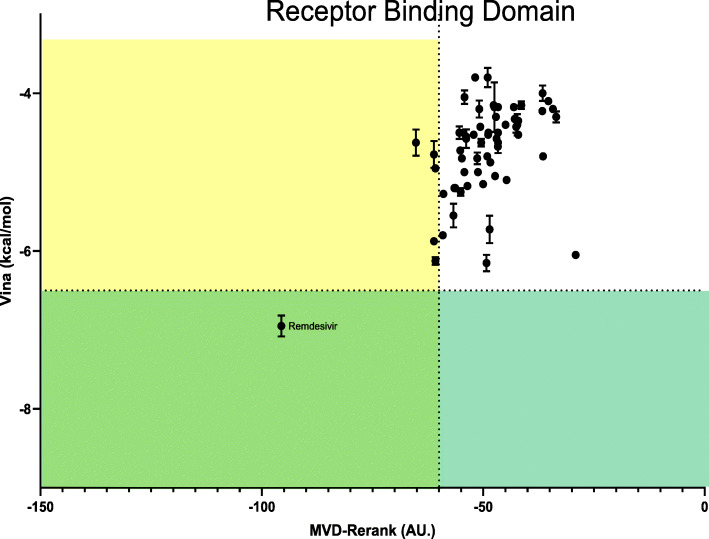
XY-scatter plot for Docking studies using Receptor Binding Domain as the target protein: Vina and MVD Rerank scores plotted as XY-scatter plot. Regions below − 6.5 kcal/mol and − 60 AU. were marked. The intersect quadrant (green) represents the molecules that satisfy both cut-offs

All 58 allergen fragrance molecules were docked to four target proteins using Vina and Molegro Virtual Docker. Results of these *in slico* experiments tabulated and ranked according to the binding affinities (data presented in supplementary materials Table S1 and Table S2). Results were compared with the five molecules (Artemisinin, Favipiravir, Hydroxychloroquine, Nigellidine, and Remdesivir) reported in the literature with the potential to be a drug against COVID-19. For Vina docking binding affinity of − 6.5 kcal/mol was used as a cut-off. On the other hand, MVD presents two docking scores Moldock score and Rerank score. According to the literature, the re-rank score was preferred to compare binding affinities. For this software, − 60 AU. was used as a cut-off score. To present molecules that can satisfy both cut-off scores, we generated XY scatter plots for all the molecules docked to four target proteins (Figs. 1, 2, 3 and 4).

When the Vina results with BRD2 protein were analyzed, seven out of 58 allergen fragrance molecules and only 3 out of five selected molecules had binding affinities ≤ − 6.5 kcal/mol. The best binding score in this experiment was for Nigellidine with − 8.20 kcal/mol binding affinity, followed by Benzyl Cinamate with − 7.30 kcal/mol, Benzyl Salicylate with − 7.20 kcal/mol and Benzyl Benzoate with − 6.98 kcal/mol. Artemisinin and Remdesivir had − 6.98 kcal/mol and − 6.90 kcal/mol affinity to BRD2, respectively. Allergen fragrance molecules; Galaxolide (− 6.95 kcal/mol), Lyral (− 6.79 kcal/mol), Beta Caryophylene (− 6.60 kcal/mol) and 3-propylidene Phthalide (− 6.50 kcal/mol) also showed good binding affinities. When the MVD scores were analyzed, 6 out of 58 allergen fragrance molecules and 3 out of 5 selected molecules, namely Remdesivir, Hydroxychloroquine, Nigellidine, Lyral, Hydroxycitronellal, Benzyl Cinnamate, Farnesol, Alpha Amyl Cinnamaldehyde, and Alpha Hexyl Cinnamaldehyde satisfied the cut-off. There were only four molecules that had a good binding affinity in both docking software; Remdesivir, Nigellidine, Lyral, and Benzyl Cinnamate, as presented in Fig. 1.

Docking results using Main Protease (MPro) as target protein showed that five out of 58 allergen fragrance molecules had binding affinities equal to or lower than − 6.5 kcal/mol. Besides the Lyral (− 6.73 kcal/mol) and Galaxolide (− 6.60 kcal/mol) that had good predicted binding to BRD2; Vertofix (alpha Acetyl Cedrene) (− 6.63 kcal/mol), Dihydroambrettolide (− 6.50 kcal/mol), and Sclareol (− 6.50 kcal/mol) had been predicted to bind to MPro. Out of the five selected potential drugs Nigellidine (− 7.40 kcal/mol), Artemisinin (− 7.20 kcal/mol), and Remdesivir (− 6.65 kcal/mol) were binding better than the cut-off. On the other hand, MVD results showed 29 molecules passing the cut-off. When we analyzed the molecules that satisfy both cut-offs, we saw three of the allergen fragrance molecules Lyral, Galaxolide, and Sclareol, while only two of the five selected molecules Nigellidine and Remdesivir satisfy these criteria (Fig. 2).

Unlike BRD2 and MPro, none of the tested molecules had binding affinities lower than − 6.5 kcal/mol to Nucleocapsid protein. The best binding compounds we tested were Artemisinin and Dihydroambrettolide, both with approximately − 6 kcal/mol binding affinity. On the other hand, MVD scores showed 10 of the allergen fragrance molecules, and three of the selected drug candidates, including Artemisinin, Favipiravir, and Hydroxychloroquine, passed the cut-off. When we analyzed Fig. 3 for the molecules that satisfy both criteria, we see that none of the tested molecules pass our elimination, thus for further docking experiments, Nucleocapsid was not considered as a potential target for allergen fragrance molecules.

Similar to Nucleocapsid, docking experiments between the Receptor Binding domain and allergen fragrance molecules did not result in promising binding affinities. While five molecules hardly had satisfactory scores following MVD, only Remdesivir satisfy both of our cut-offs (Fig. 4). That is why, like Nucleocapsid, we conclude that the receptor binding domain is not a potential target for allergen fragrance molecules.

### Flexible docking

For the 10 selected allergen fragrance molecules and five molecules (Artemisinin, Favipiravir, Hydroxychloroquine, Nigellidine, and Remdesivir) presented in the literature with a potential to be a drug against COVID-19, binding pocket amino acids of BRD2 and MPro proteins were picked to be flexible during Vina simulations. Binding affinities of these 15 molecules were calculated and presented in Table 2.

**Table 2:** Binding affinities of the 15 molecules used in flexible docking studies (kcal/mol)

	BRD2			MPro		
	Lowest	Mean	SEM	Lowest	Mean	SEM
3-PROPYLIDENE PHTHALIDE	−6.7	− 6.63	0.07	− 7.0	− 6.83	0.09
**BENZYL BENZOATE**	**−8.1**	**−7.95**	**0.10**	−6.3	−5.83	0.17
**BENZYL CINNAMATE**	**−8.3**	**−8.30**	**0.00**	−6.8	−6.65	0.07
**BENZYL SALICYLATE**	**−8.1**	**−7.88**	**0.09**	−6.9	−6.70	0.07
BETA CARYOPHYLLENE	−7.4	− 7.40	0.00	−5.4	− 5.33	0.03
**DIHYDROAMBRETTOLIDE**	**−8.0**	**− 7.83**	**0.17**	−7.1	−6.97	0.09
**GALAXOLIDE**	**−8.6**	**− 8.40**	**0.10**	−7.4	−7.37	0.03
LYRAL	−6.8	− 6.63	0.06	−6.7	−5.58	0.39
**SCLAREOL**	−7.5	− 7.37	0.07	**−8.0**	**−7.73**	**0.15**
VERTOFIX (ALPHA ACETYL CEDRENE)	−6.9	−6.77	0.09	−7.2	−7.00	0.12
Artemisinin	−7.9	−7.75	0.09	−7.4	− 7.15	0.13
Favipiravir	−6.0	−5.75	0.22	−6.0	− 5.50	0.30
Hydroxychloroquine	−7.1	−6.85	0.10	−6.6	−6.30	0.12
**Nigellidine**	**−8.8**	**−8.50**	**0.18**	**− 8.3**	**−7.83**	**0.21**
**Remdesivir**	**−8.9**	**−8.50**	**0.16**	**−8.4**	**− 8.10**	**0.18**

Flexible docking results showed that Nigellidine and Remdesivir had very good binding affinities against both target proteins BRD2 and MPro. The other three drugs re-tested in our docking studies showed binding affinities higher than − 8 kcal/mol. Though Artemisinin was very close to the − 8 kcal/mol cut-off, Favipiravir and Hydroxychloroquine showed binding affinities suggesting that the target for these molecules is unlikely the two proteins we used in this study. Five out of 10 allergen fragrance molecules tested had binding affinities − 8 kcal/mol or better to BRD2. Suggesting that, these molecules could tightly bind to BRD2. Especially Galaxolide, (which is a trade name of International Flavors & Fragrances company) had comparable binding affinities to Nigellidine and Remdesivir, suggesting that it might be a good candidate.

On the other hand, only one allergen fragrance molecule Sclareol showed low binding affinity (− 8 kcal/mol) to MPro with the two drug candidates Nigellidine and Remdesivir that had binding affinities − 8.3 kcal/mol and − 8.4 kcal/mol, respectively.

When the interacting amino acids analyzed using Ligplot+ software, that generate diagrams of protein-ligand interactions [39], we see that same or similar amino acids of the target proteins interact with the allergen fragrance molecules and drug candidates suggesting that they have the same binding pocket with similar binding affinities (supplementary Fig. 1).

**Fig. 5:**
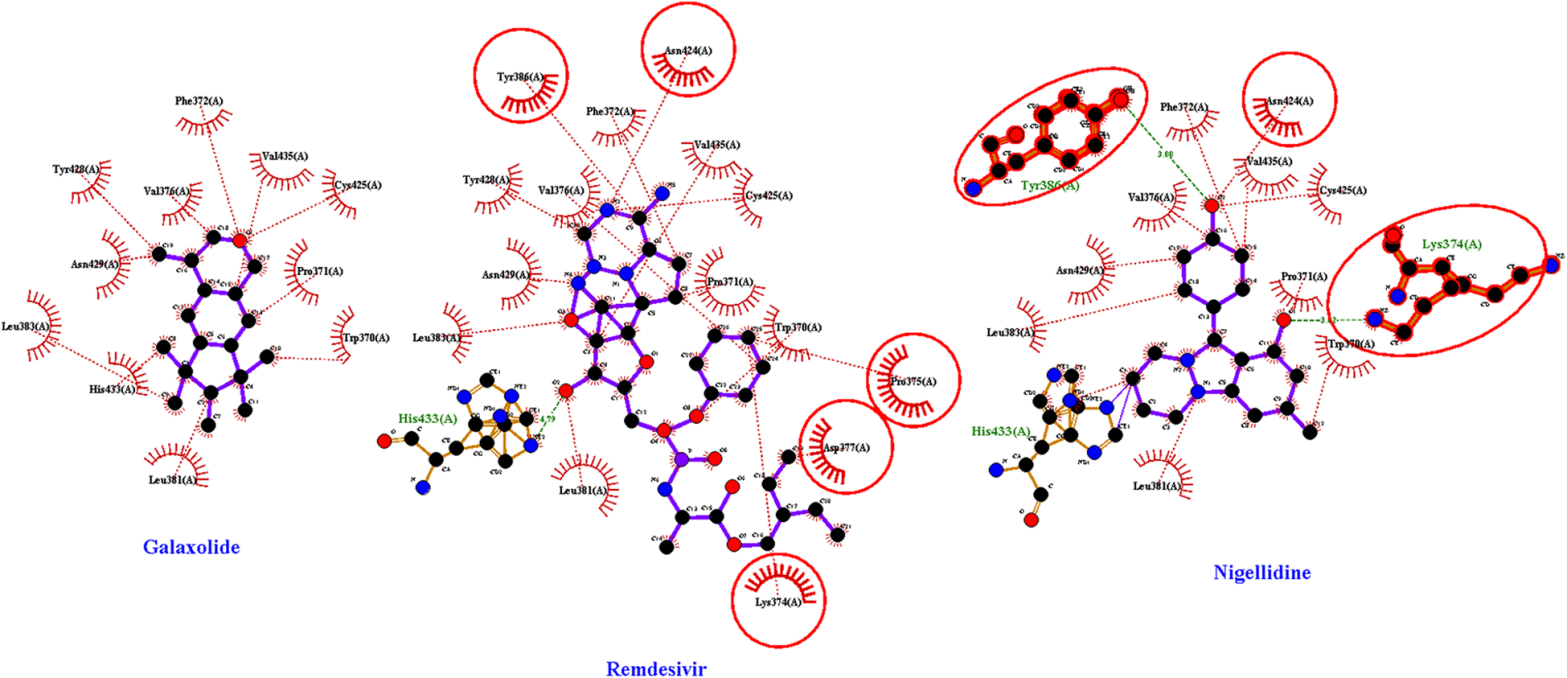
LigPlot+ diagrams of protein-ligand interactions for Galaxolide, Remdesivir, and Nigellidine: Using BRD2 as the target protein amino acids that can generate hydrogen bonds with the ligand were printed in green. An arc represents hydrophobic contacts with spokes radiating towards the ligand atoms they contact. Unique amino acids for Remdesivir and Nigellidine were circled with a red line

Figure 5 shows the interacting amino acids of BRD2 with Galaxolide, Nigellidine, and Remdesivir. Unlike Remdesivir and Nigellidine, Galaxolide is missing a hydrogen bond with BRD2. However, it had good hydrophobic interactions with 11 amino acids in the binding pocket. On the other hand, Nigellidine had two, and Remdesivir had one possible hydrogen bond with the target protein.

**Fig. 6:**
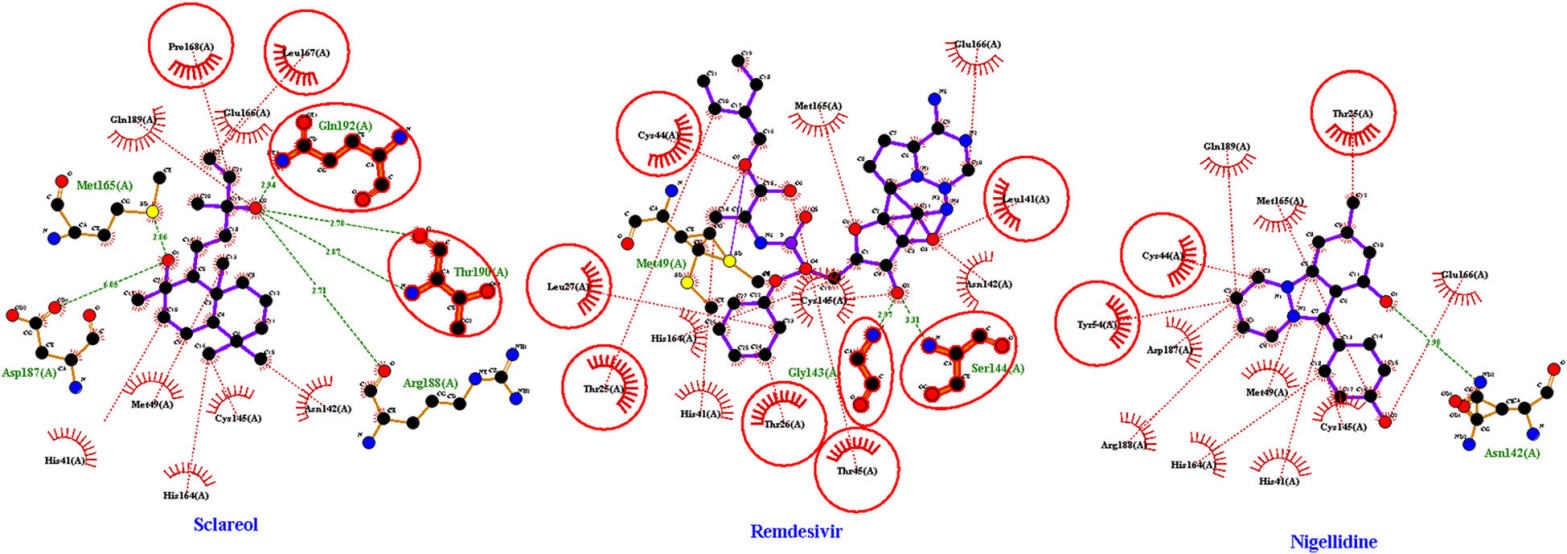
LigPlot+ diagrams of protein-ligand interactions for Sclareol, Remdesivir, and Nigellidine Using MPro as the target protein amino acids that can generate hydrogen bonds with the ligand were printed in green. An arc represents hydrophobic contacts with spokes radiating towards the ligand atoms they contact. Different amino acids possibly interacting with molecules were circled with a red line

Similarly, when we analyzed the interactions with MPro, we see that Sclareol share interactions with 10 amino acids that had potential connections with the drug candidates Remdesivir and Nigellidine (Fig. 6). It showed proximity and possible hydrogen bond with Met165, Asp187, Arg188, Thr190, and Gln192.

Flexible docking simulations suggested that allowing more degree of rotational freedom around the binding site permits the molecules to bind with better affinities to the target proteins. Further docking studies using Molecular dynamic or/and Montecarlo simulations could lead to more accurate binding calculations. However, in this study, we aimed to quickly scan many candidate molecules and identify potential lead compounds that would be further tested by in vitro and in vivo experiments.

### AutoDock

Following the virtual screening studies carried out, the selected 15 molecules were used in AutoDock simulations. When the binding affinities obtained were sorted, we see that Remdesivir had very good binding to BRD2 with − 9.58 kcal/mol binding affinity (Table 3). Subsequently, Hydroxychloroquine, Nigellidine, and Artemisinin with − 7.74 kcal/mol, − 7.50 kcal/mol and − 7.33 kcal/mol, respectively. Similar to flexible docking, Galaxolide had one of the best binding affinities among the allergen fragrance molecules with − 7.27 kcal/mol potential binding affinity. The lowest binding affinity calculated for Benzyl Cinnamate was − 7.35 kcal/mol, while the average for four separate dockings was − 7.16 kcal/mol.

**Table 3:** Binding affinities of the 15 molecules used in AutoDock studies (kcal/mol)

	BRD2		MPro	
	Mean	Lowest	Mean	Lowest
3-PROPYLIDENE PHTHALIDE	−6.65	− 6.66	− 5.64	− 5.65
BENZYL BENZOATE	− 6.73	− 6.87	− 6.22	− 6.36
**BENZYL CINNAMATE**	**− 7.16**	**− 7.35**	− 6.62	− 6.65
BENZYL SALICYLATE	− 6.40	− 6.81	− 6.07	−6.43
BETA CARYOPHYLLENE	−6.24	−6.24	− 6.38	−6.38
**DIHYDROAMBRETTOLIDE**	**− 7.21**	**− 7.22**	**− 7.13**	**−7.14**
**GALAXOLIDE**	**−7.27**	**−7.27**	**− 7.07**	**−7.08**
LYRAL	−5.91	−6.16	−6.07	− 6.48
**SCLAREOL**	−6.84	− 6.94	**−8.06**	**− 8.89**
**VERTOFIX (ALPHA ACETYL CEDRENE)**	− 6.32	− 6.40	**−7.87**	**− 7.88**
**Artemisinin**	**−7.32**	**− 7.33**	**− 7.04**	**−7.04**
**Favipiravir**	**−4.77**	**− 4.83**	**−4.74**	**− 4.86**
**Hydroxychloroquine**	**−7.59**	**−7.74**	**− 6.51**	**−6.51**
**Nigellidine**	**−7.50**	**− 7.50**	**− 7.12**	**−7.12**
**Remdesivir**	**−9.34**	**−9.58**	**−7.73**	**−7.73**

Similar to BRD2, Remdesivir had the best binding to MPro among the drug candidates reported in the COVID-19 literature. However, unlike BRD2, some of the tested allergen fragrance molecules, namely Sclareol and Vertofix, had batter binding affinities to MPro then the five drug candidates used in this study with − 8.89 kcal/mol and − 7.88 kcal/mol, respectively (Table 3).

**Fig. 7:**
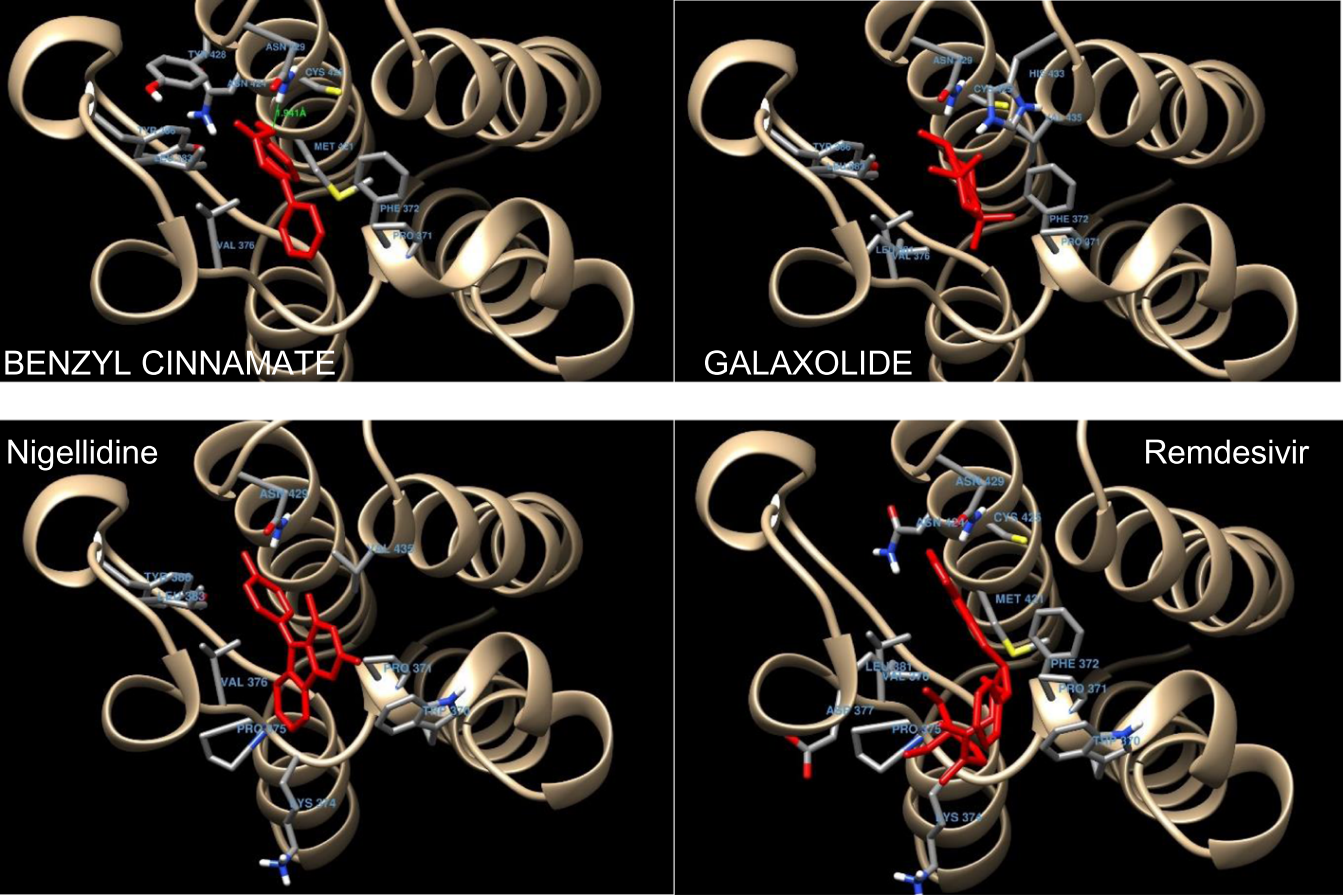
AutoDock binding poses of Benzyl Cinnamate, Galaxolide, Nigellidine, Remdesivir: Using BRD2 as the target protein amino acids in the interaction distance to the ligand were labeled in the dock poses. Docked ligands colored in red

The binding poses for all the tested molecules were analyzed using autodock tools, and screenshots were recorded to visualize the possible interacting residues (supplementary Fig. 2). When we compare Benzyl Cinnamate, Galaxolide, Nigellidine, and Remdesivir docking pose on BRD2, we see that many of the binding pocket residues had proximity to the ligand molecule as expected (Fig. 7). Asn424 showed potential hydrogen bonding with Benzyl Cinnamate and Remdesivir.

**Fig. 8:**
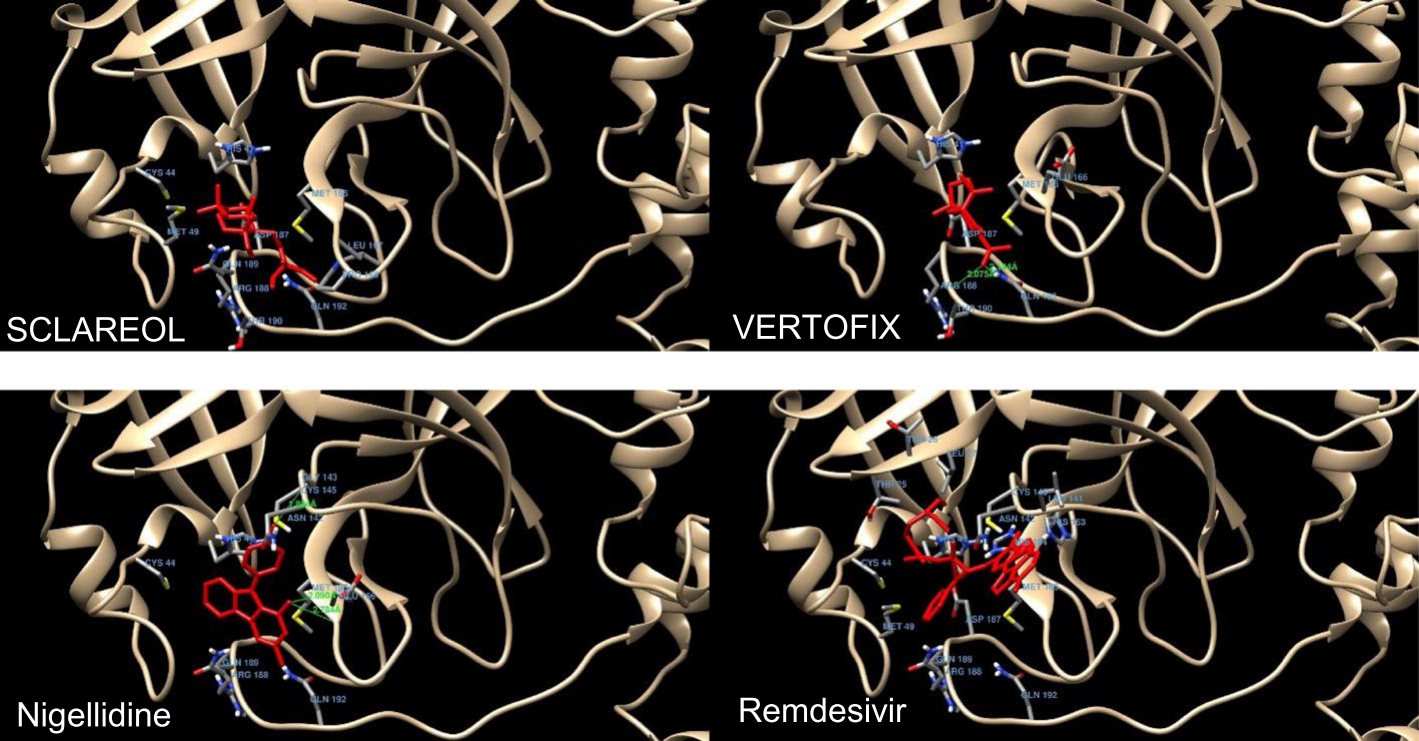
AutoDock binding poses of Sclareol, Vertofix, Nigellidine, Remdesivir: Using MPro as the target protein amino acids in the interaction distance to the ligand were labeled in the dock poses. Docked ligands colored in red

The top-scored two drug molecules and two allergen fragrance molecules were posed on MPro, similar to the BRD2 docking. Results indicate that His41, Met165, and Arg188 were shared by all four ligands, whereas Remdesivir and Nigellidine have some unique interacting residues (Fig. 8). Different from the other three, Sclareol likely to interact with Leu167 and Pro168.

These results suggest that allergen fragrance molecules had good if not better binding affinities to BRD2 and MPro target proteins compared to the five drug molecules tested. The binding pocket and possibly the interacting residues with the ligands were shared in most cases, while some unique residues specific for individual ligands were identified.

## Discussion

The ongoing SARS-CoV-2 threat that emerged in China has rapidly spread Worldwide and continuing to spread as of today. It is apparent that unless a vaccine or treatment discovered, the virus will continue to threaten humanity. While many vaccine studies are ongoing worldwide, discussions on the effectiveness and logistics of the distribution are growing every day. Besides the potential protective effect of a possible vaccine, it has limited applications for current COVID-19 patients. Thus, many efforts have been directed to investigate a drug suitable for preventing and/or treating human SARS-CoV-2. The implementation of anti-viral treatment and protection has several requirements: First of all, the stock of the drug must be sufficient and readily available; secondly, the safety of therapy should be tolerated, and finally, the cost should be affordable. As the fourth requirement under a pandemic situation, the drug needs to be discovered in a short period of time. Considering the severity of the pandemic, and urgency to find relief, many drug studies focused on the pre-existing drug molecules that have already satisfy most of these requirements.

With the same motivation in the present study, potential anti-viral effects of 58 allergen fragrance molecules on COVID-19 were investigated by docking simulations. For this purpose, four functional proteins that have structure information submitted to RCSB-PDB and reported as essential for SARS-CoV-2 were picked as target proteins in our docking studies. These proteins include the COVID-19 Main Protase (MPro), Receptor binding domain of spike protein, Nucleocapsid, or the N-protein and host Bromodomain protein (BRD2).

Fragrance molecules are a mixture of natural essential oils and synthetic organic odorous compounds with characteristics, usually pleasant odors. They are used in perfumes and scented cosmetic products, in detergents, soaps, fabric softeners, air care, incense, and other household products.

Essential oils (volatile oils) are aromatic oily liquids obtained from plant materials (flowers, buds, seeds, leaves, twigs, bark, herbs, wood, fruits, and roots) [40]. These oils have been theorized to evolve with viruses, bacteria, and fungi to protect plants and trees from viral, bacterial, and fungal infections. Thus, if we look up the anti-viral behavior of plants, we can easily see that the defense mechanism is based on essential oils. The viricidal activity of essential oils, which are lipophilic by nature, is due to disruption of the viral membrane or interference with viral envelope proteins involved in host cell attachment. Consequently, many of these essential oils have been used in various cultures for medicinal and health purposes, food preservation, pharmaceuticals, alternative medicine, and natural therapies for centuries. Under physiological stresses, pathogen attacks, and ecological factors, plants produce essential oils and gums. In addition, oils can be obtained by expression, fermentation, extraction, and distillation. Some of these essential oils have antibacterial, antifungal, anti-viral, insecticidal, and antioxidant properties [41, 42]. They have been used in cancer treatment [43], in food preservation [44], aromatherapy [45], and fragrance industries [46]. More importantly, studies showed that oils such as *L. nobilis* oil exhibited an effective action against the SARS-CoV-1 virus with an IC_50_ value of 120 mg/ml [22]. Although it is not emphasized in these studies, some of the major constituents of these essential oils, reported to possess strong antiviral properties, belong to fragrance allergens subgroup.

Analysis of docking studies, carried by three different software and total of four different methods, showed that several of the tested molecules showed low binding affinities, as good as or better than the drug candidate molecules against COVID-19, presented in current literature. Following the virtual screening and lead identification studies presented in this study, lead optimization and clinical studies could initiate the discovery of new drug(s) that can potentially prevent and/or cure COVID-19 infection.

## Conclusion

Our results showed that many of the allergen fragrance molecules tested in docking simulations had potential binding affinities to four target proteins used. Compared to the reported molecules, Artemisinin, Favipiravir, Hydroxychloroquine, Nigellidine, and Remdesivir, several of these molecules had as good as, if not better, binding affinities against especially BRD2 and MPro.

Although Receptor binding domain (RBD) of spike protein and Nucleocapsid were both potential viral targets used in several other studies in the literature, our results did not indicate a strong interaction between the tested molecules and these proteins. On the other hand, Covid19 Main Protase (MPro) and host Bromodomain protein (BRD2) had good binding affinities for several of the tested molecules. When the binding patterns are analyzed, hydrophobic interactions and sporadic hydrogen bonds stabilize the ligands in the binding pocket. For BRD2, Asn429 is often observed in a hydrogen bond with the docked ligand, whereas His 433, Lys 374, and Asp 377 are occasionally involved in hydrogen bonding. Compared to BRD2, MPro binding pocket contains more polar and charged amino acids. Among these, His 163, Gln 189, and 192 observed in hydrogen bonding, while some of the other polar and charged amino acids, such as Arg 188, involved in orienting the ligands in the pocket.

Benzyl Cinnamate, a naturally occurring molecule that is present in Tolu and Benzoin Resinoids; Dihydroambrettolide (16 -Hexadecanolide), a synthetic musk product used in the fragrance industry and Galaxolide (4,6,6,7,8,8 – Hexamethyl - 1,3,4,6,7,8 - hexahydrocyclopenta[g] isochromene isomers) a trade name of International Flavors & Fragrances company, which is a synthetic musk smelling product widely used from household to fine fragrance compositions that do not occur in nature, had good binding affinities to BRD2. While Dihydroambrettolide and Galaxolide showed potential to bind to MPro, too; Sclareol a naturally occurring product mostly found in *Salvia sclare* extracts which, nowadays can also be synthesized by the biochemical route and mainly used as starting material of Natural Ambergris main ingredient of Ambrox; and Vertofix a trademark name of International Flavors & Fragrances company which is a synthetic product produced from Cedarwood oil acetylation and used in all applications of fragrance as woody scent; had the best calculated binding affinities to this target.

When the flexible docking results analyzed, all the molecules tested had better calculated binding affinities as expected in addition to the three potential molecules identified by AutoDock results Benzyl Benzoate, a naturally occurring product found in white flowers and resinoid extracts and. Benzyl Salicylate, one of the highest volumes used fragrance ingredients; where, for the fragrance industry, mainly the synthesized version is used, showed comparable binding affinities to BRD2. In the case of MPro, Sclareol had the lowest binding affinity among all the tested allergen fragrance molecules.

In conclusion, these allergen fragrance molecules, which are readily available, cost-efficient and shown to be safe for human use, alone or in combinations could be used in air-conditions, space nebulizer, electrical diffusers, reed diffusers, aerosols, cologne, liquid soaps, household cleaning products, etc. as an anti-viral supplement. Results indicate that at correct doses, these molecules could have significant health benefits through inhalation. Further, in vitro and in vivo studies could help us develop potential lead compounds as an anti-viral drug in respiratory applications against COVID-19.

## Supplementary Information

**Additional file 1: Table S1.** Mean binding affinity (kcal/mol) calculated by Vina: Selected top 9 compounds in this study, previously reported compounds, and binding affinities ≤ − 6 kcal/mol were printed in bold letters. **Table S2.** Reranked scores from Molegro Virtual Docker: Top nine molecules from Vina results and previously reported compounds printed in bold letters. **Supplementary Figure 1.** Figures for Flexible Docking with BRD2. **Supplementary Figure 2.** Figures for Flexible Docking with MPro. **Supplementary Figure 3.** Figures AutoDock poses for BRD2. **Supplementary Figure 4.** Figures AutoDock poses for MPro

**Additional file 2.** SwissADME results: SwissADME.

**Additional file 3.** PASSonline Toxicology results: allergen toxicology data.

**Additional file 4.** PASSonline probability of being active or inactive (Pa, Pi) results: pa-pi activity all.

## Data Availability

The datasets used and/or analysed during the current study available from the corresponding author on reasonable request.

## Abbreviations

3TC: Lamivudine
ACE2: Angiotensin-converting enzyme 2
BRD2: Host Bromodomain protein
COVID-19: Coronavirus disease 2019
CoVs: Coronaviruses
FDA: Food and Drug Administration
MERS-CoV: Middle East respiratory syndrome coronavirus
MPro: Covid19 Main Protase
MVD: Molegro Virtual Docker
Nsps: Nonstructural proteins
RBD: Receptor binding domain
RCSB-PDB: Research Collaboratory for Structural Bioinformatics Protein Data Bank
RNAi: RNA interference
RTC: Replicase-transcriptase complex
SARS-CoV-2: Severe acute respiratory syndrome coronavirus 2
SARS: Severe acute respiratory syndrome
SCCS: Scientific Committee on Consumer Safety
SEM: Standard error of the mean
TDF: Tenofovir disoproxil
WHO: World Health Organization
