# Supplementary material for: Allergen fragrance molecules: a potential relief for COVID-19
Source: BMC Complement Med Ther. 2021 Jan 21;21:41. doi: 10.1186/s12906-021-03214-4 (PMC7819625; doi:10.1186/s12906-021-03214-4)
Supplement: Supplementary file 1 — Additional file 1: Table S1. Mean binding affinity (kcal/mol) calculated by Vina: Selected top 9 compounds in this study, previously reported compounds, and binding affinities ≤ − 6 kcal/mol were printed in bold letters. Table S2. Reranked scores from Molegro Virtual Docker: Top nine molecules from Vina results and previously reported compounds printed in bold letters. Supplementary Figure 1. Figures for Flexible Docking with BRD2. Supplementary Figure 2. Figures for Flexible Docking with MPro. Supplementary Figure 3. Figures AutoDock poses for BRD2. Supplementary Figure 4. Figures AutoDock poses for MPro [file 12906_2021_3214_MOESM1_ESM.docx]

**Supplementary Materials:**

**SF1: Figures for Flexible Docking with BRD2.**

| 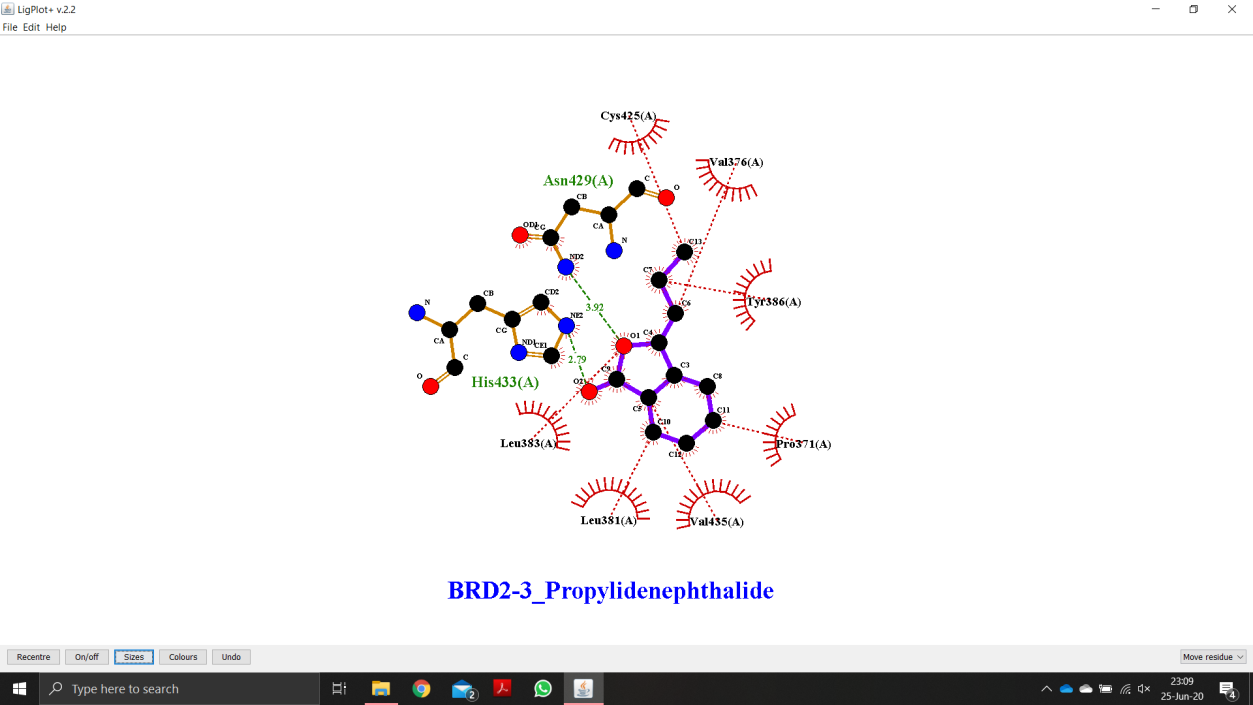 | **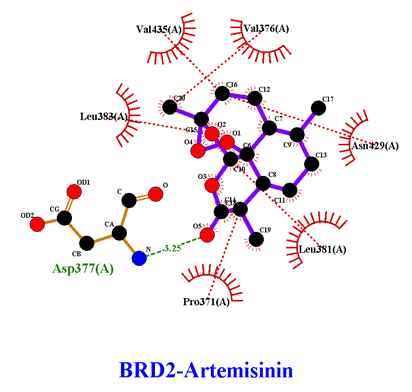** | 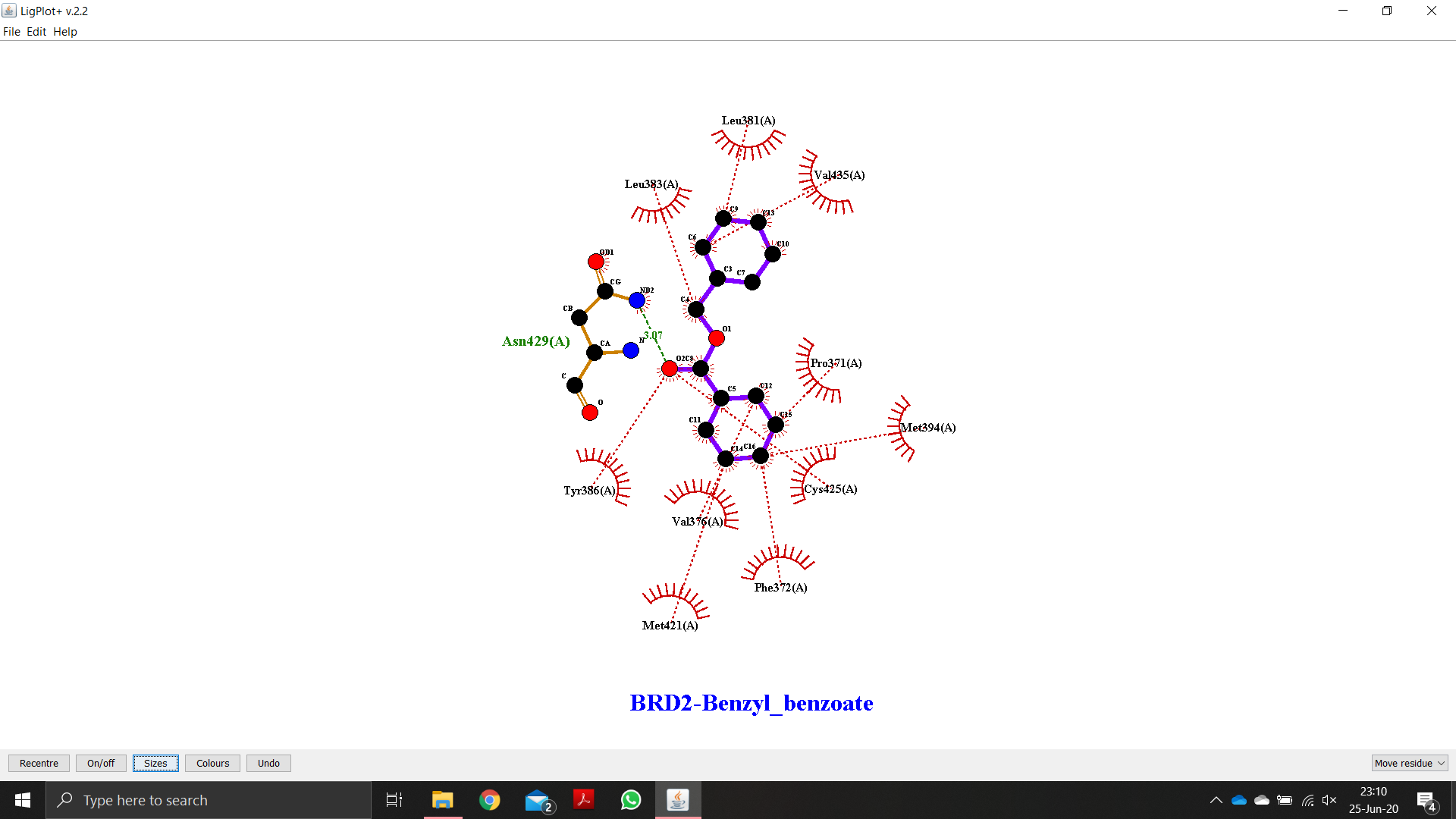 |
| --- | --- | --- |
| 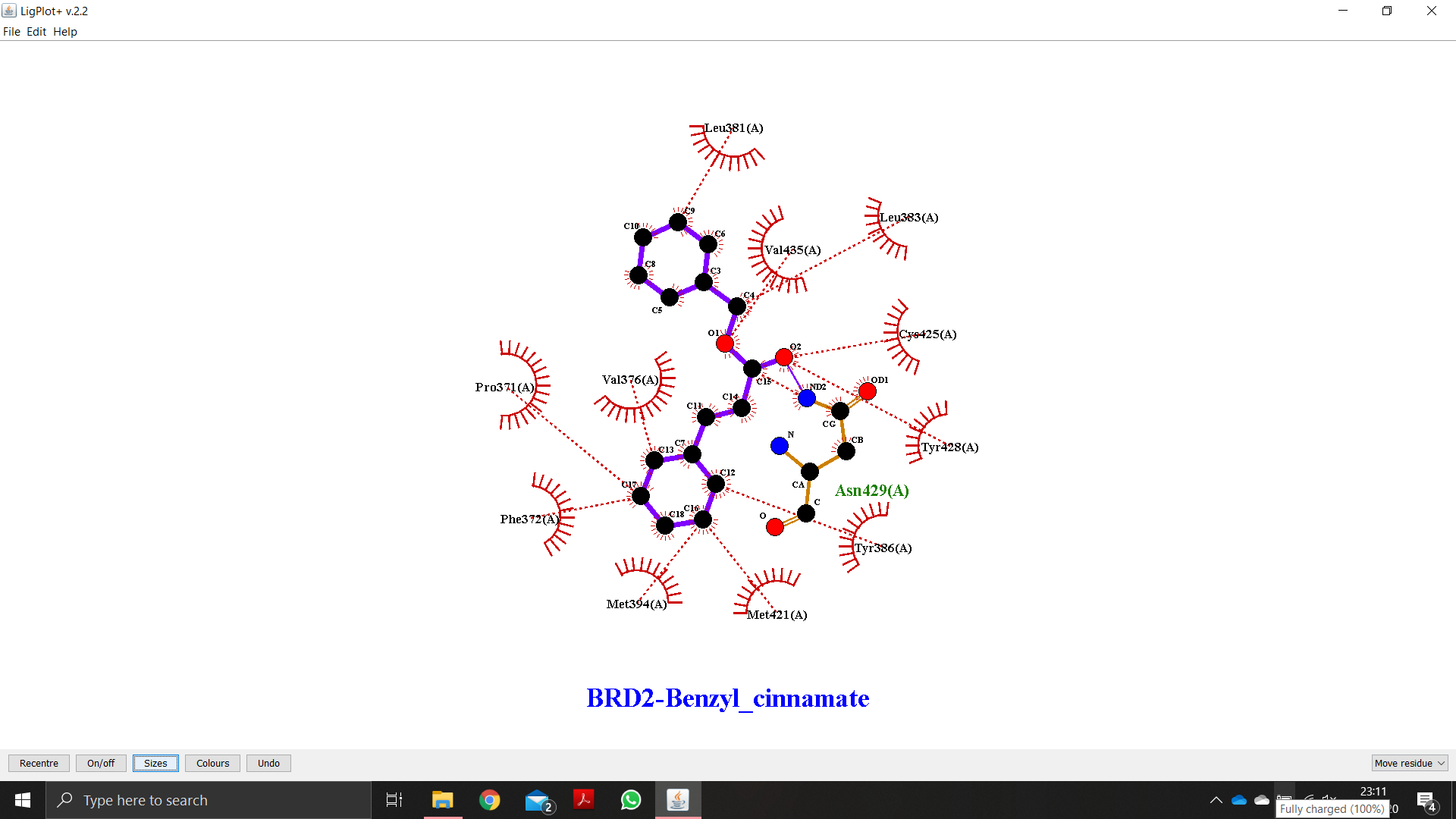 | 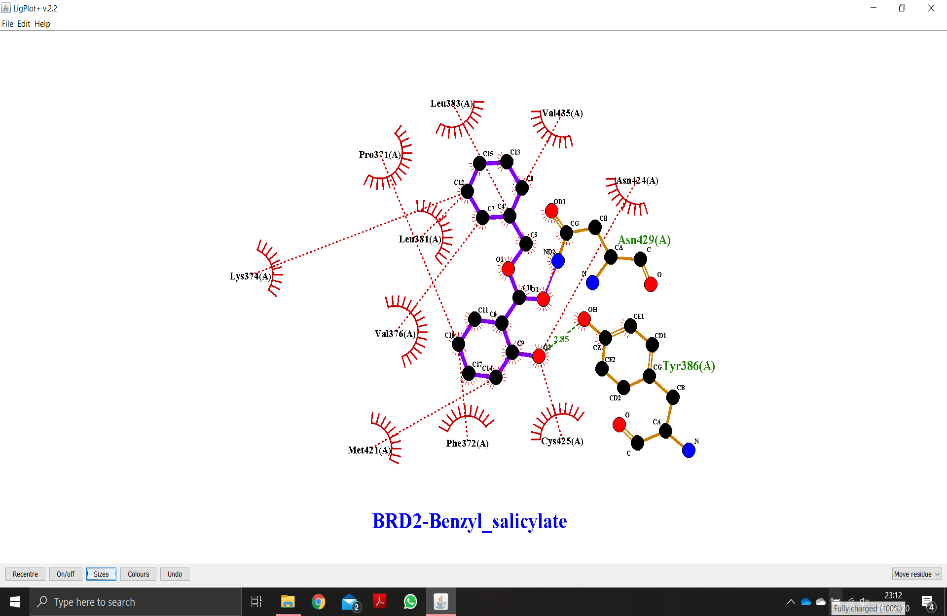 | 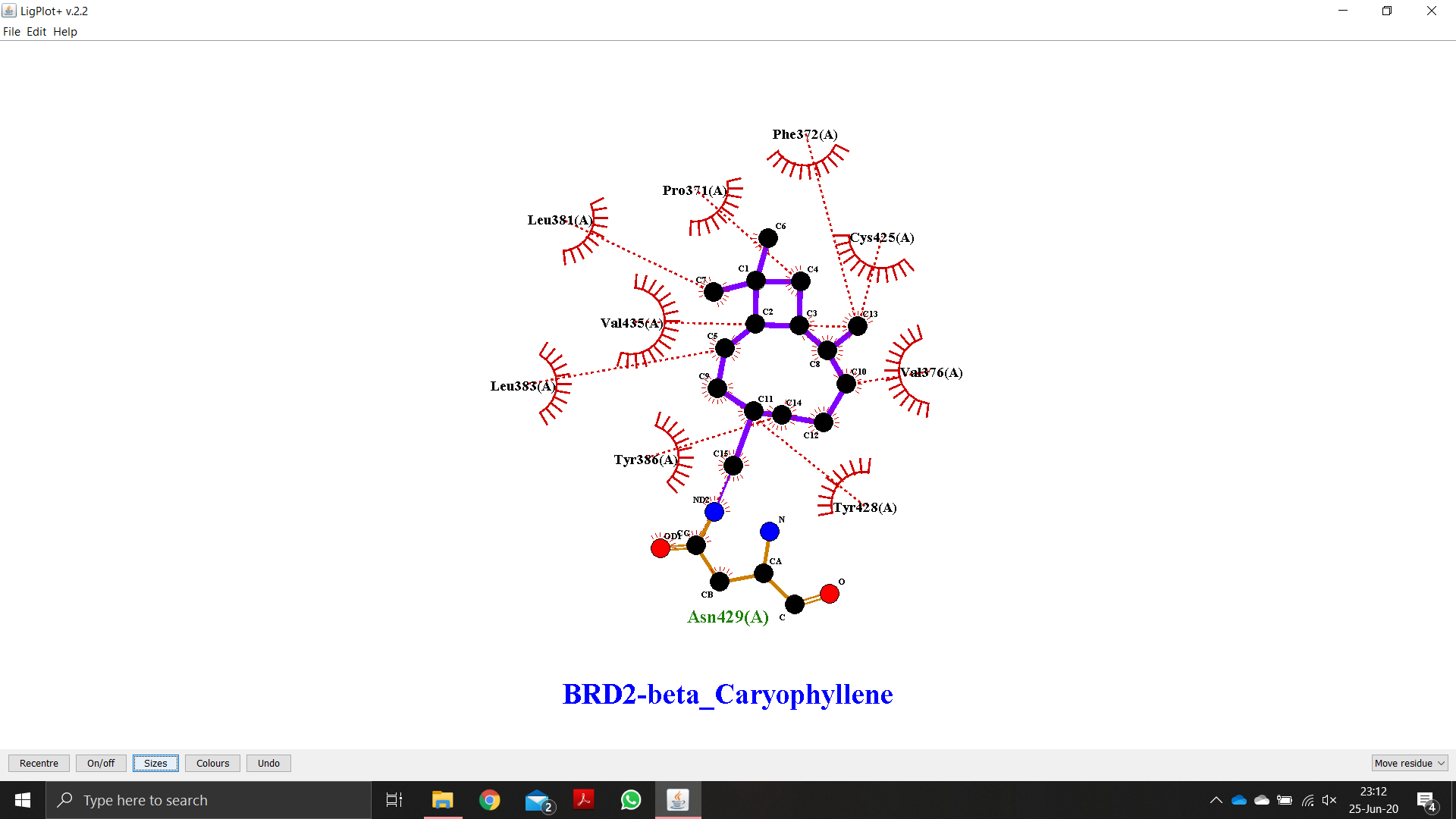 |
| 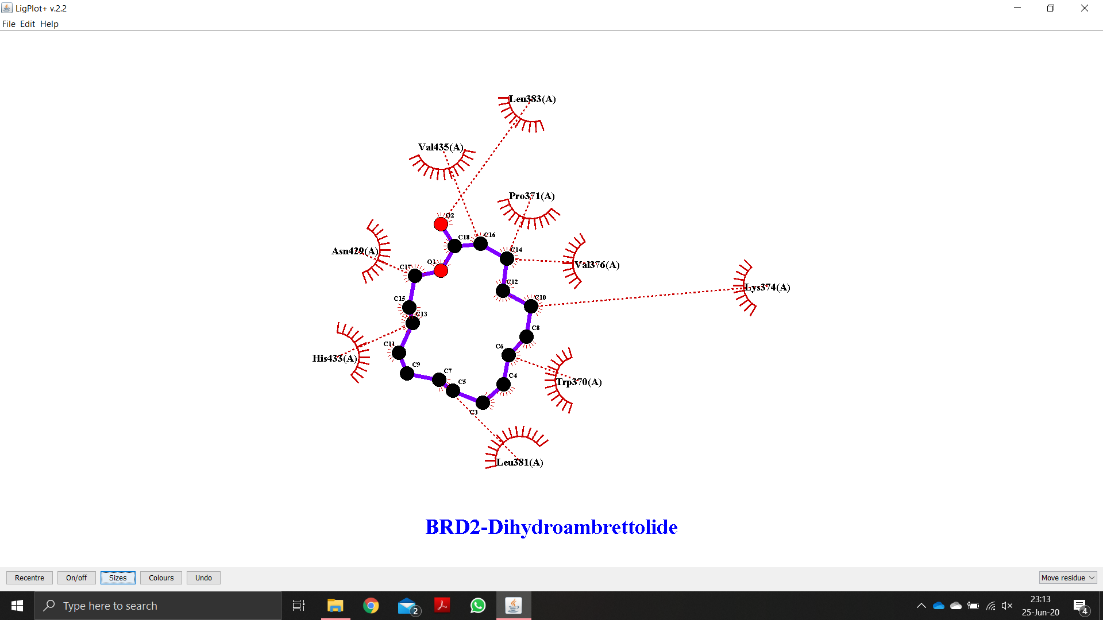 | 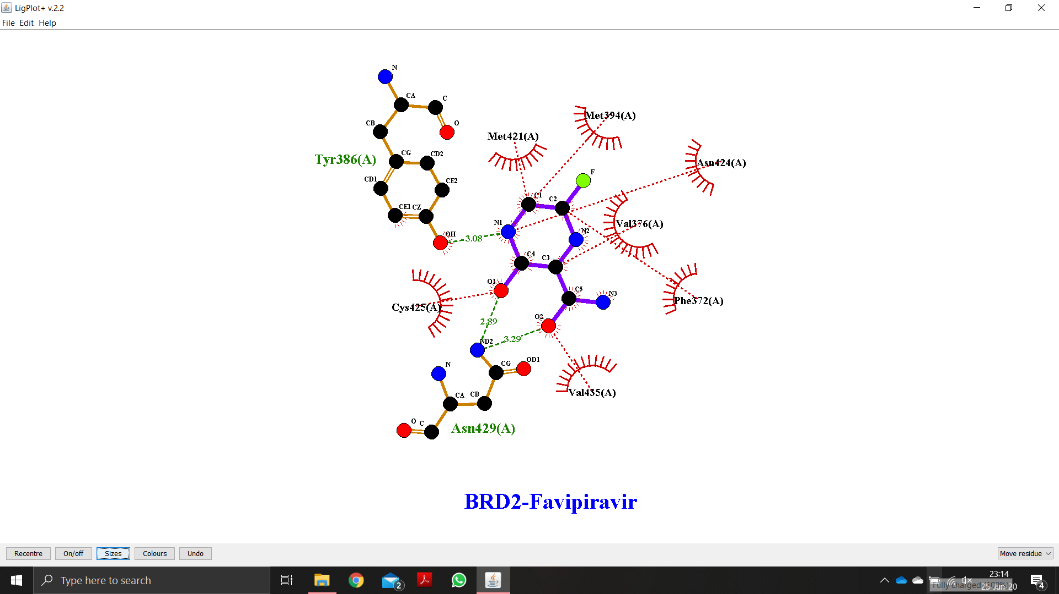 | 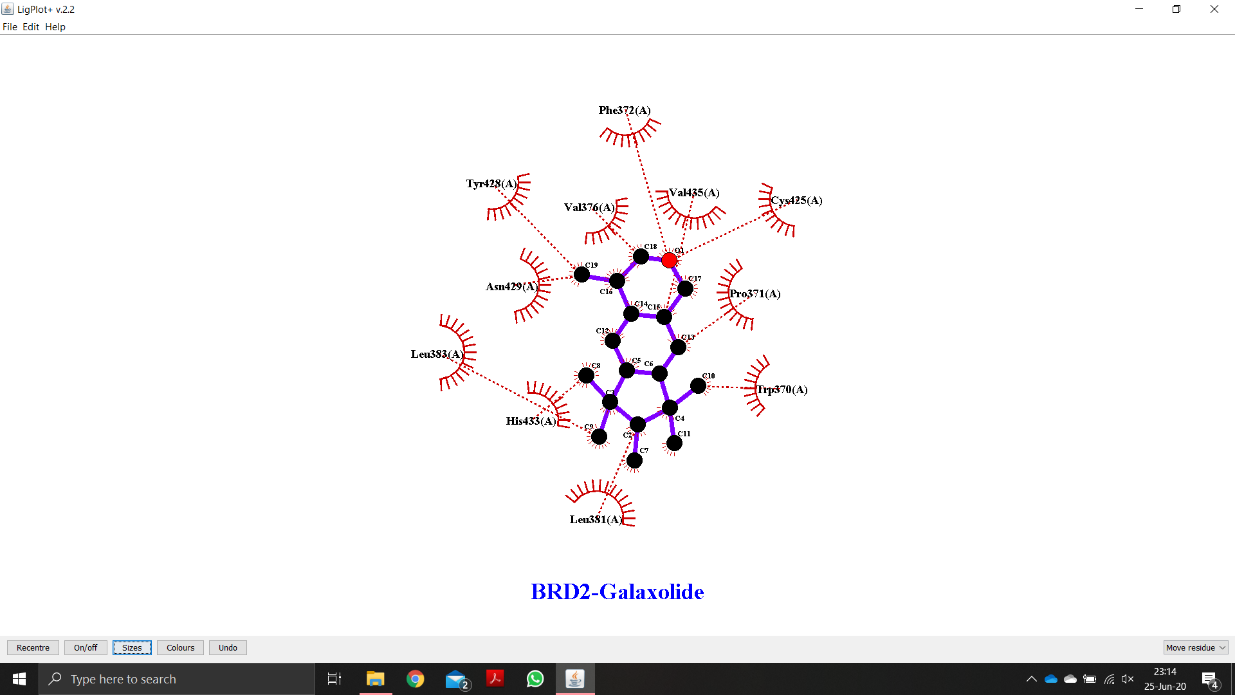 |
| 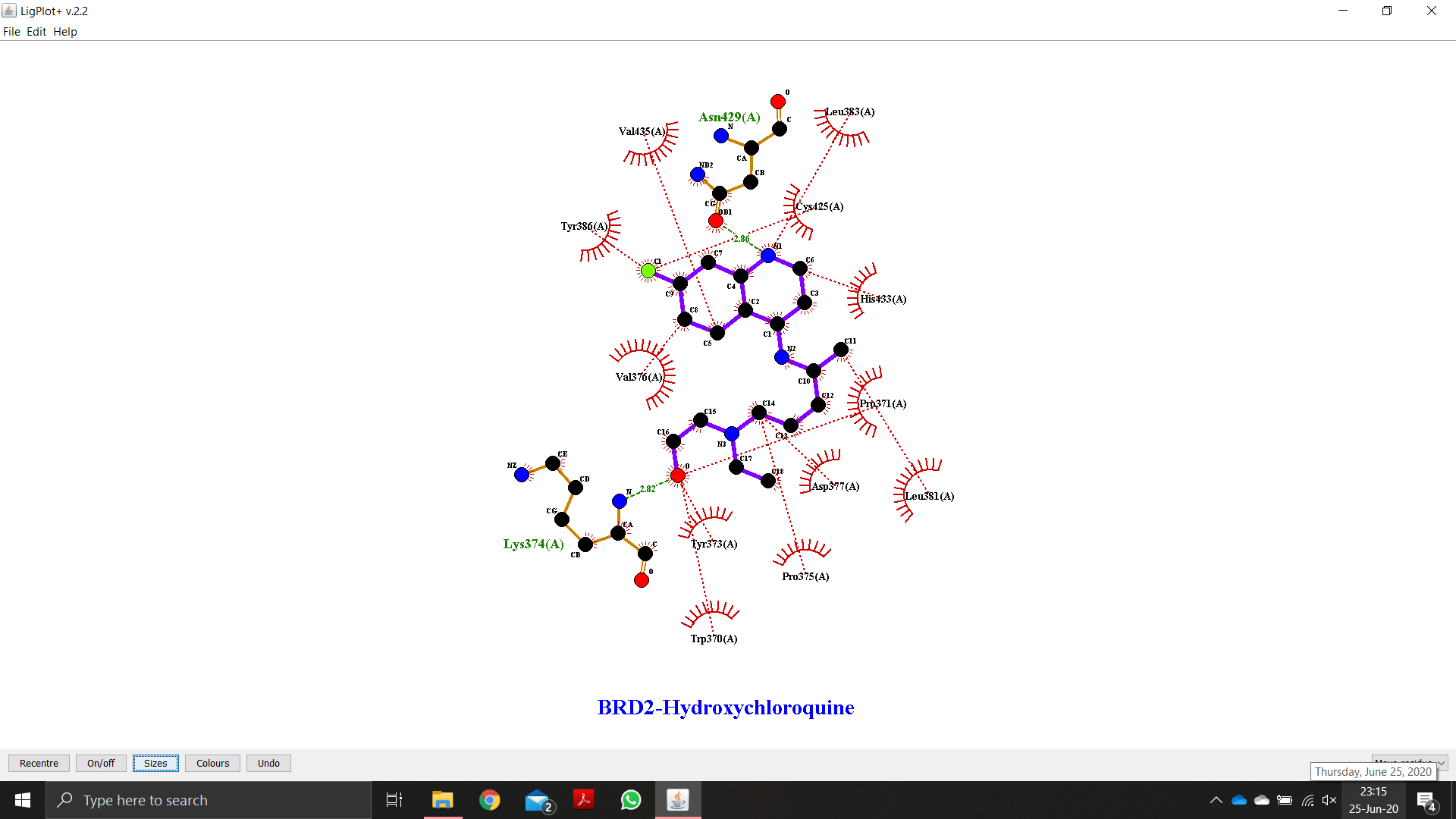 | 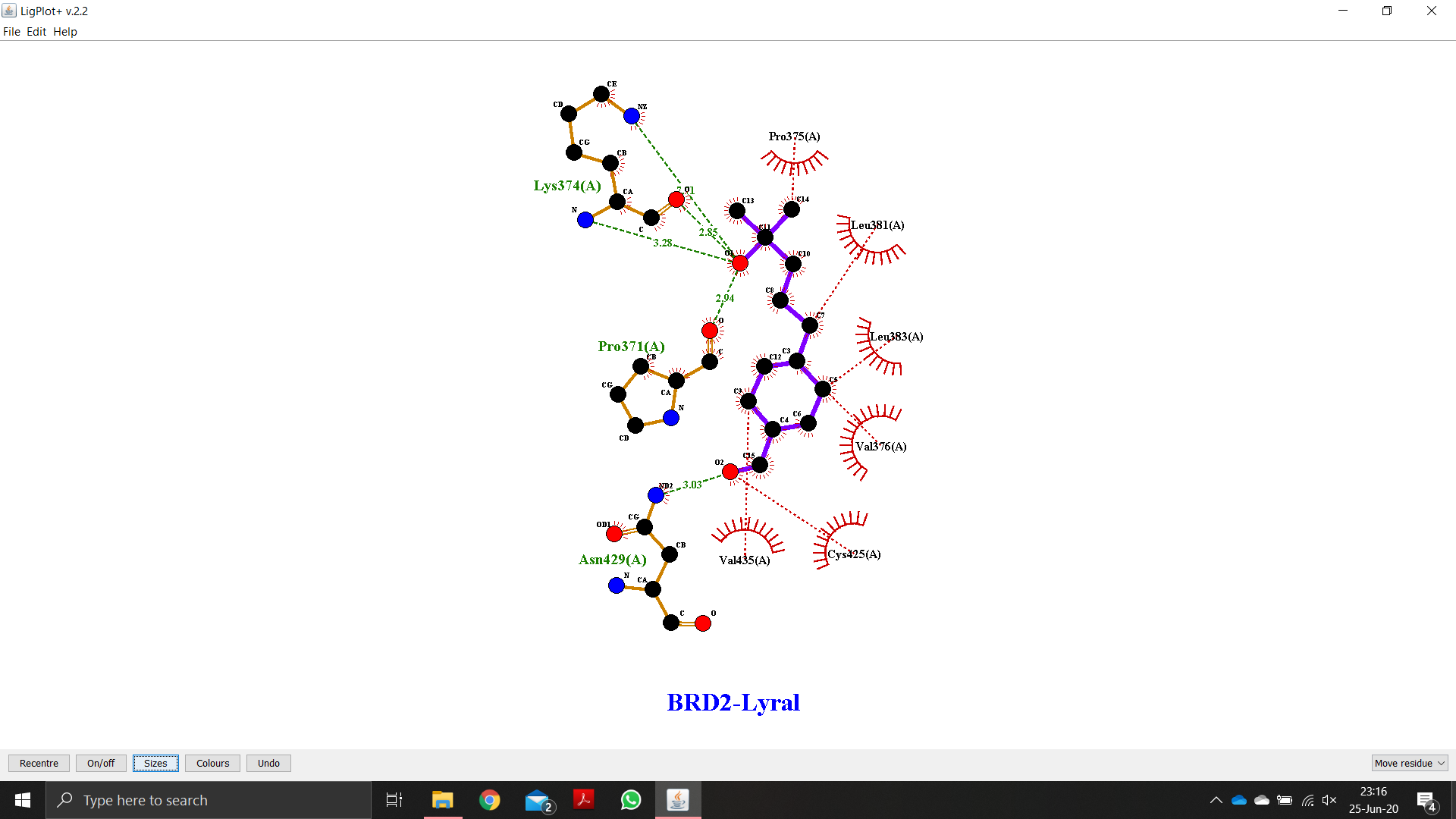 | 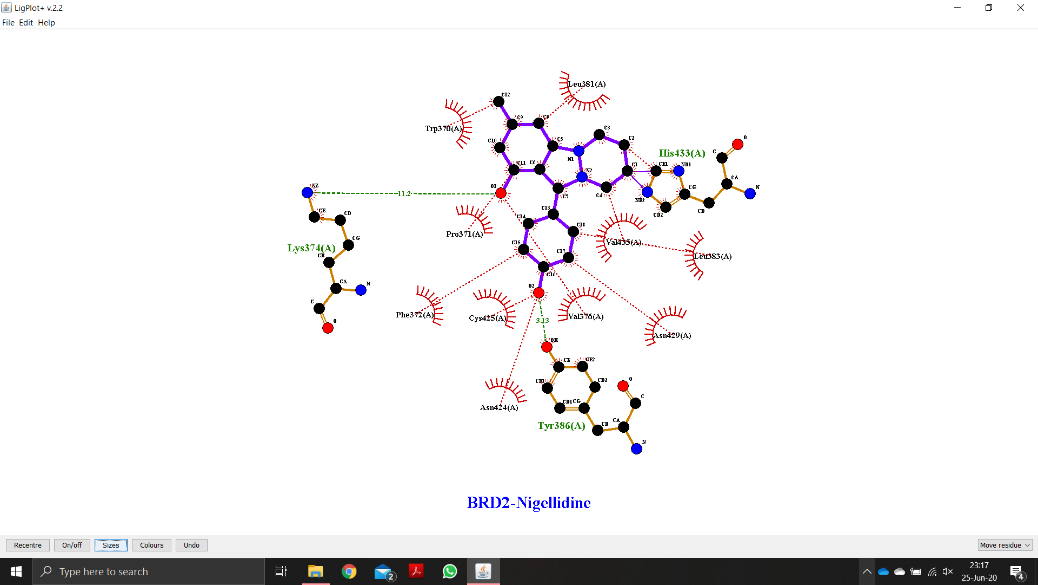 |
| 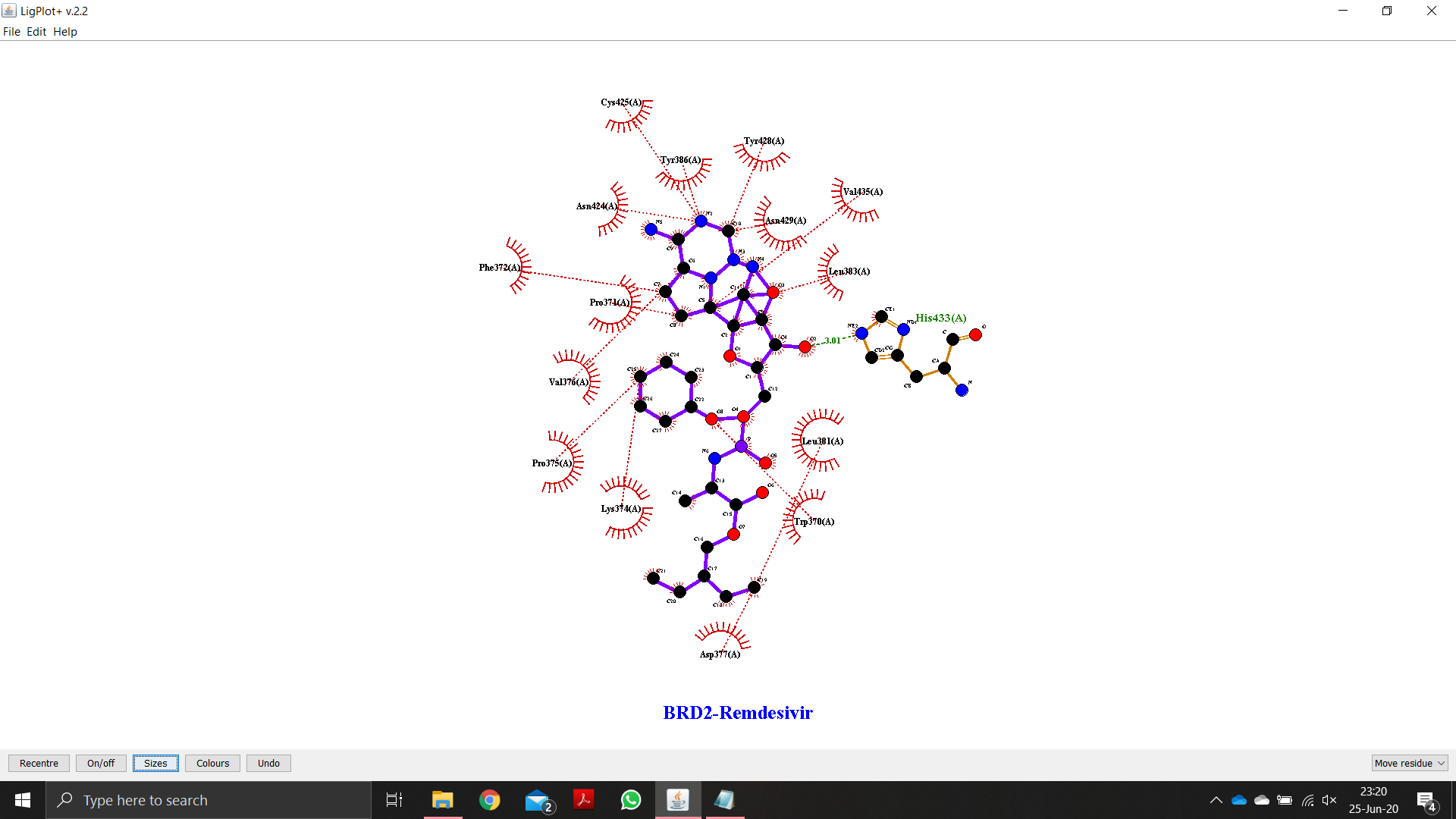 | 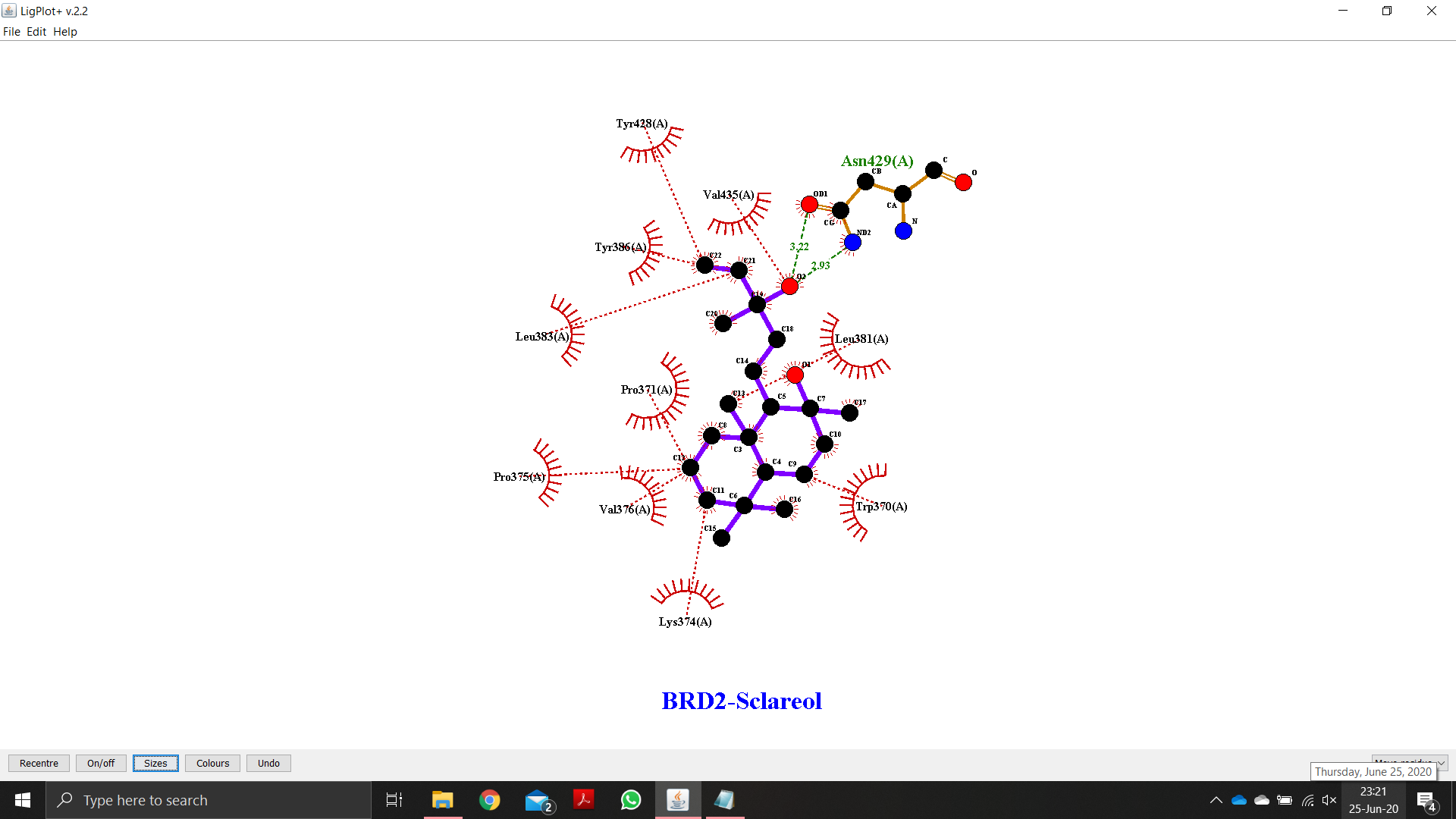 | 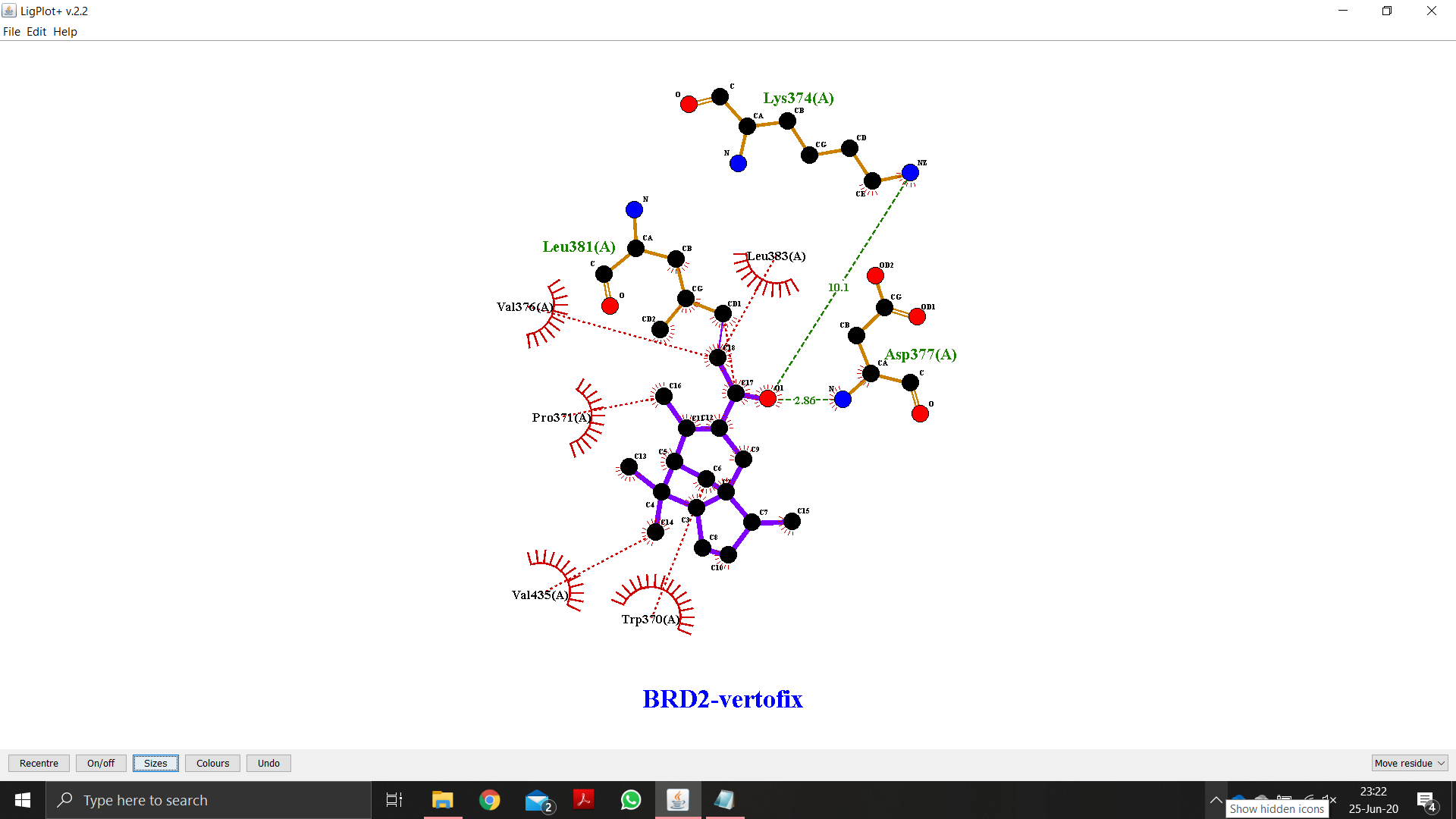 |

**SF2: Figures for Flexible Docking with Mpro.**

| 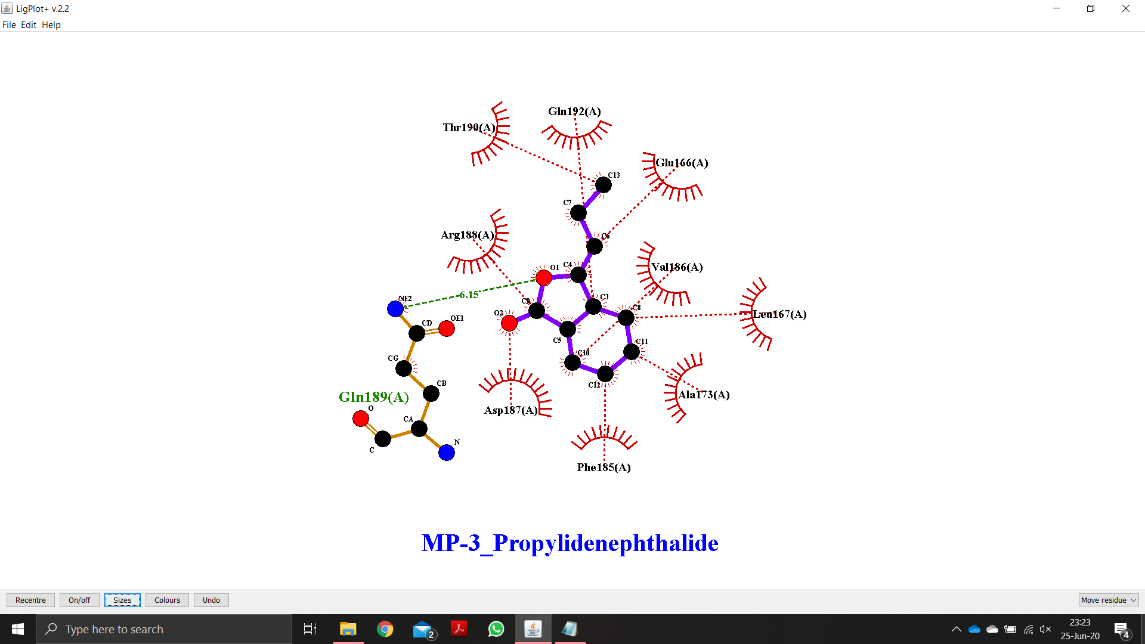 | 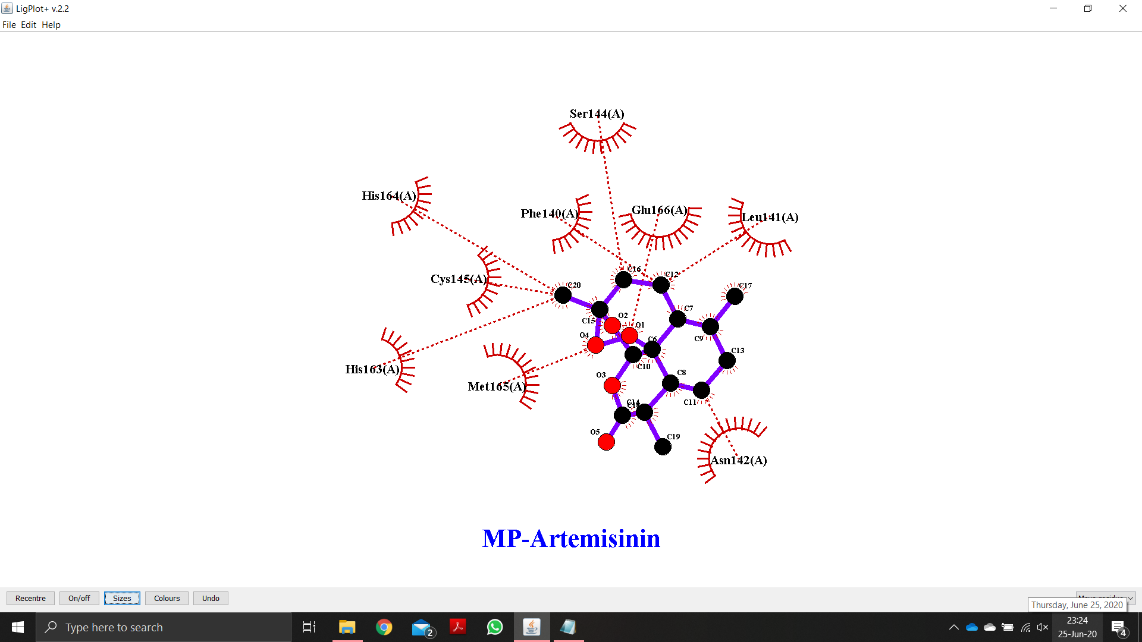 | 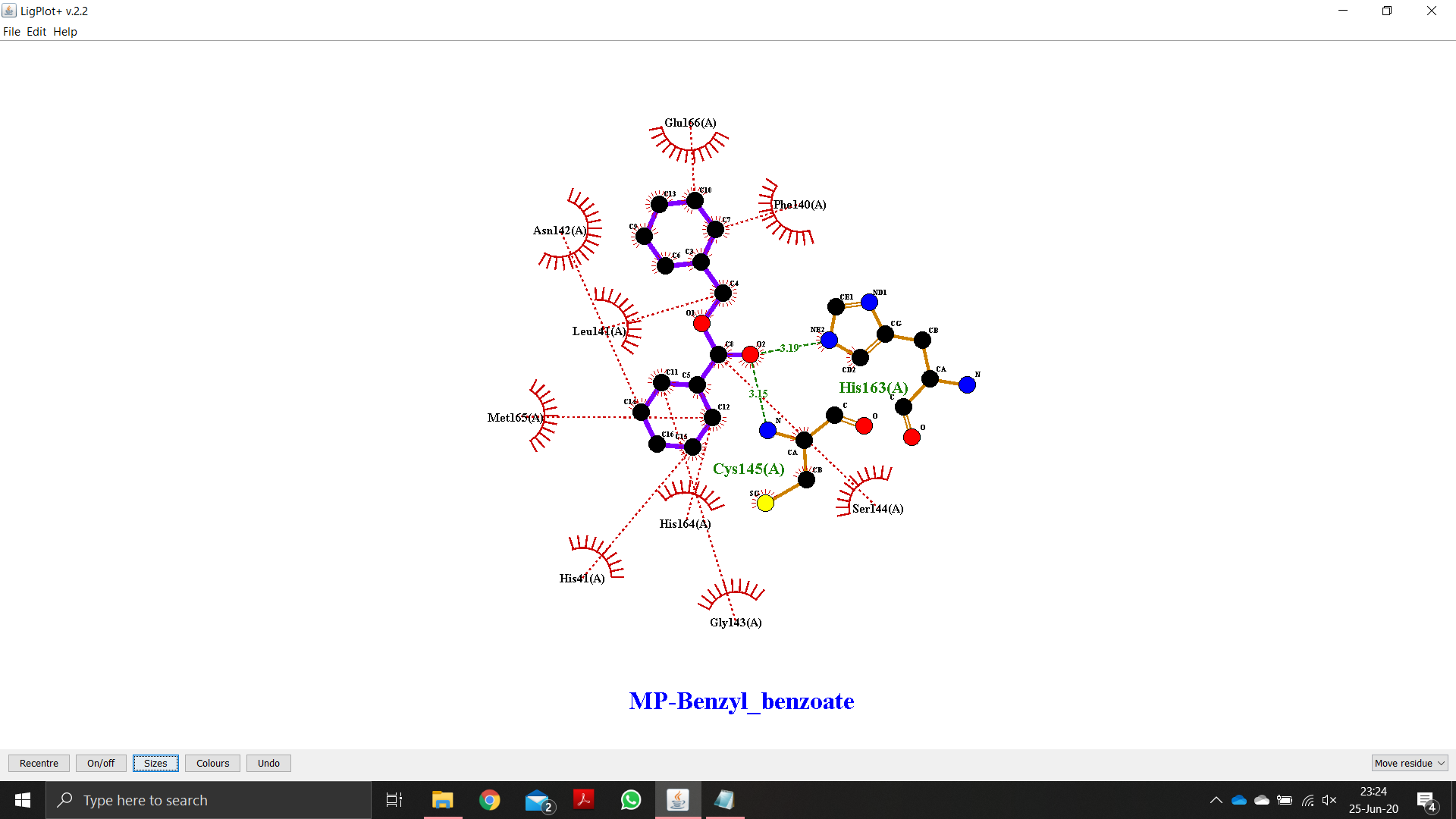 |
| --- | --- | --- |
| 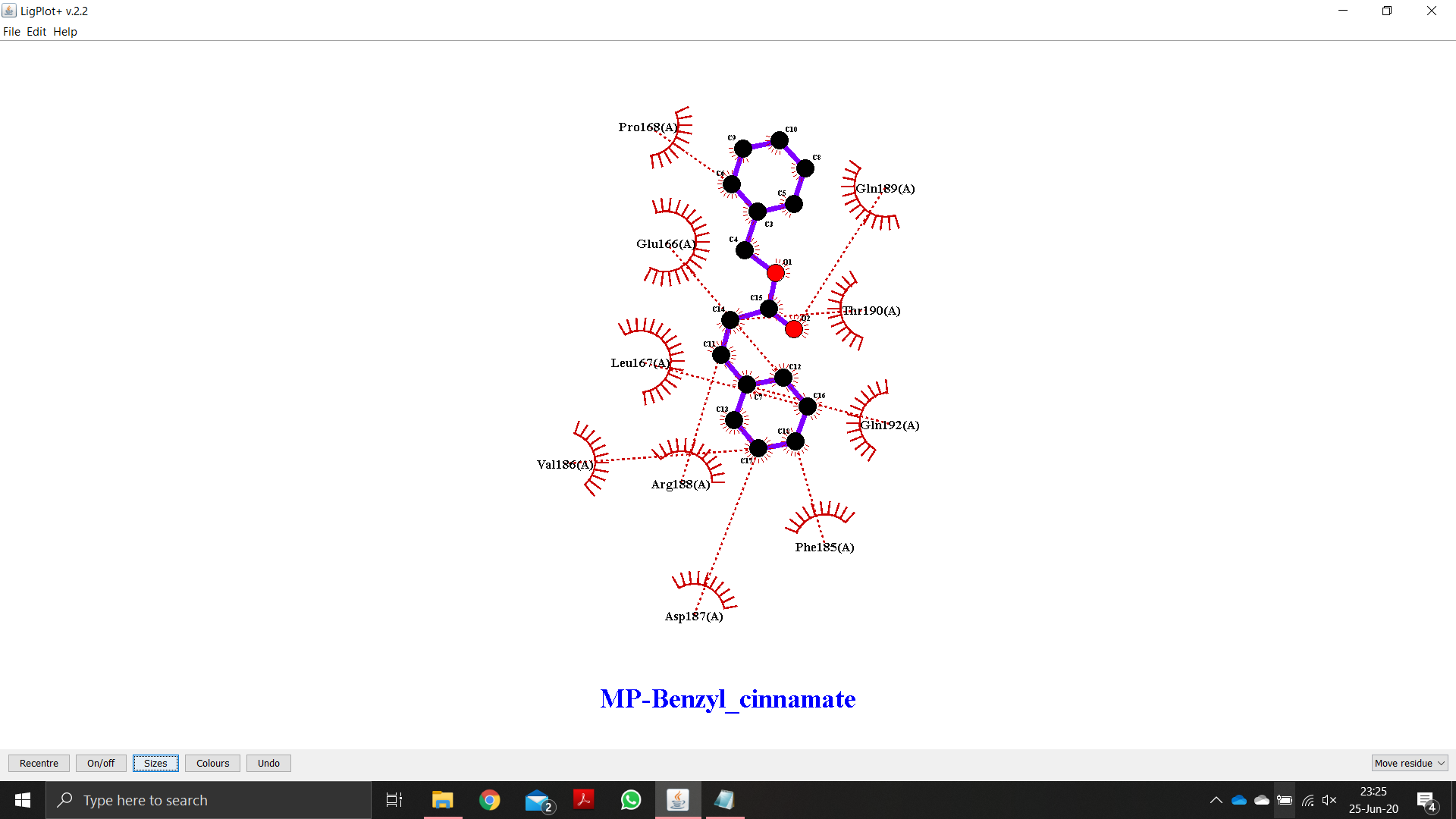 | 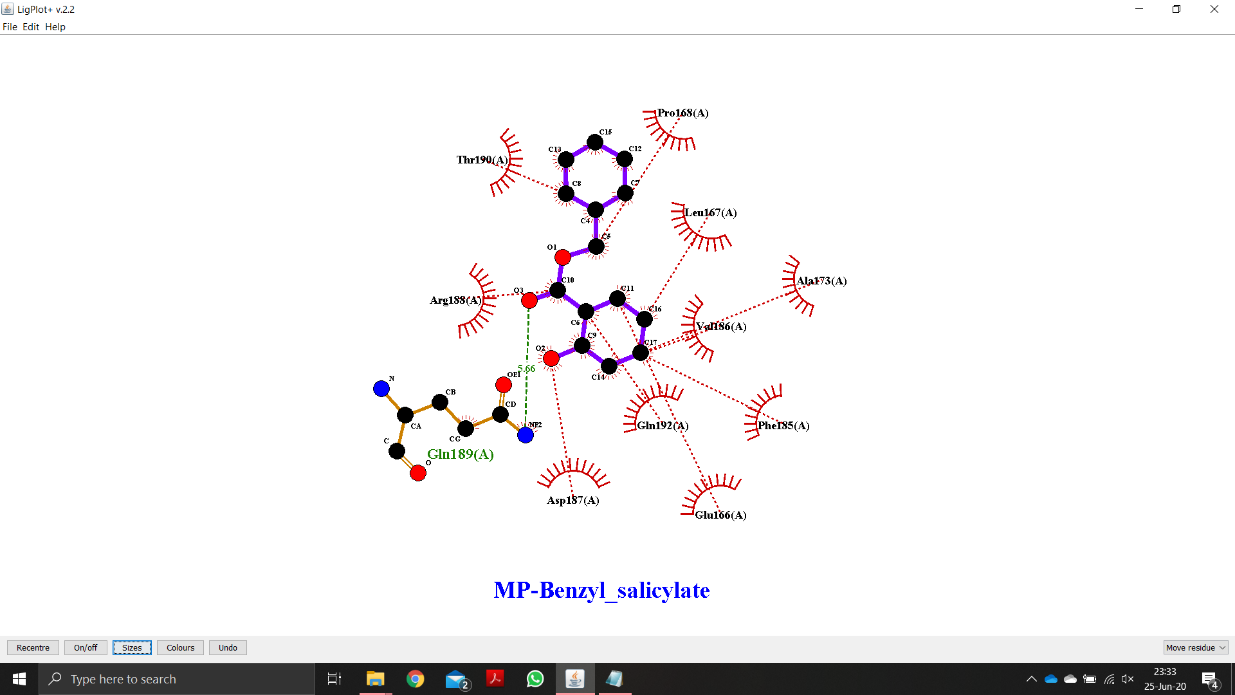 | 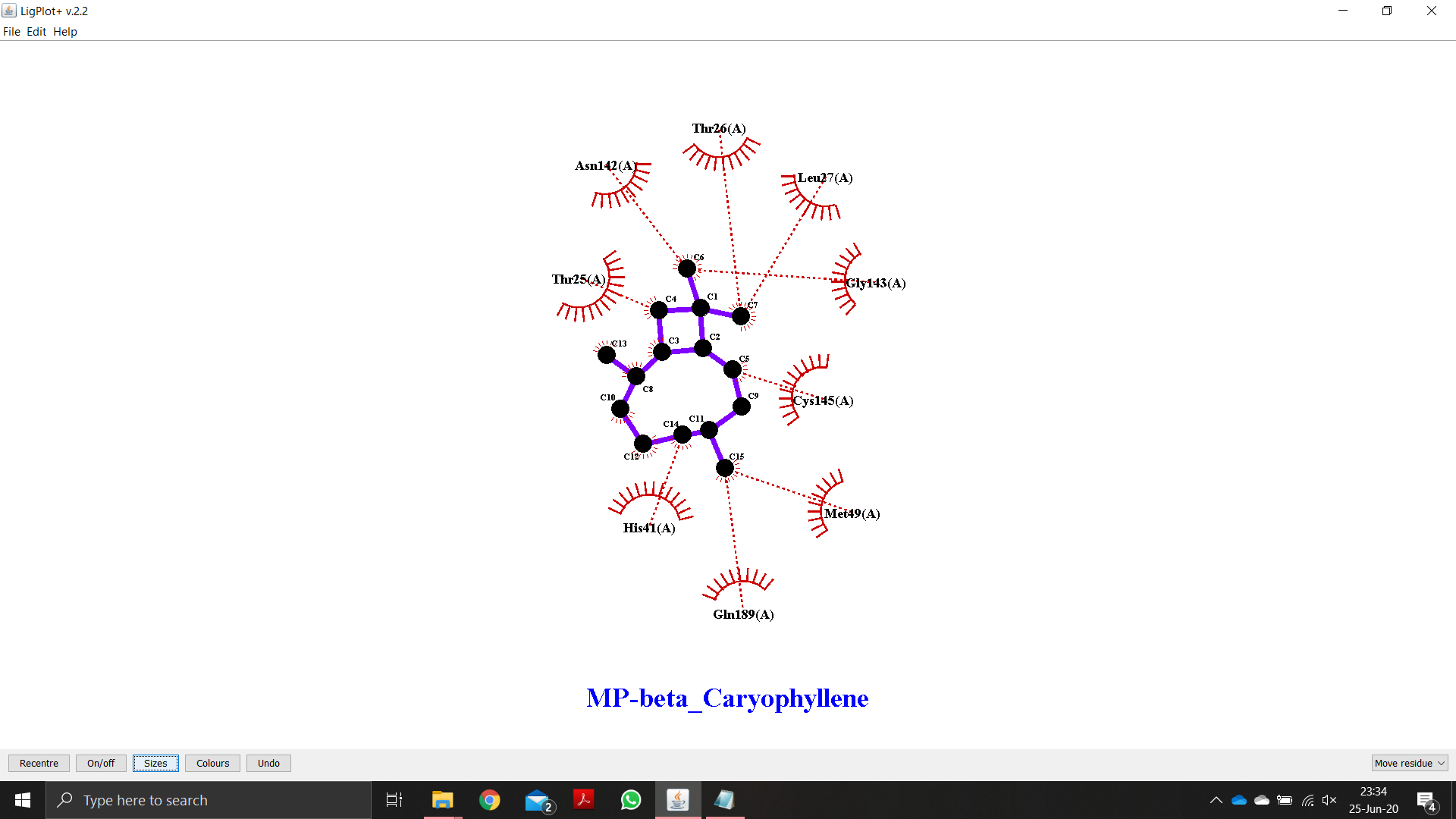 |
| 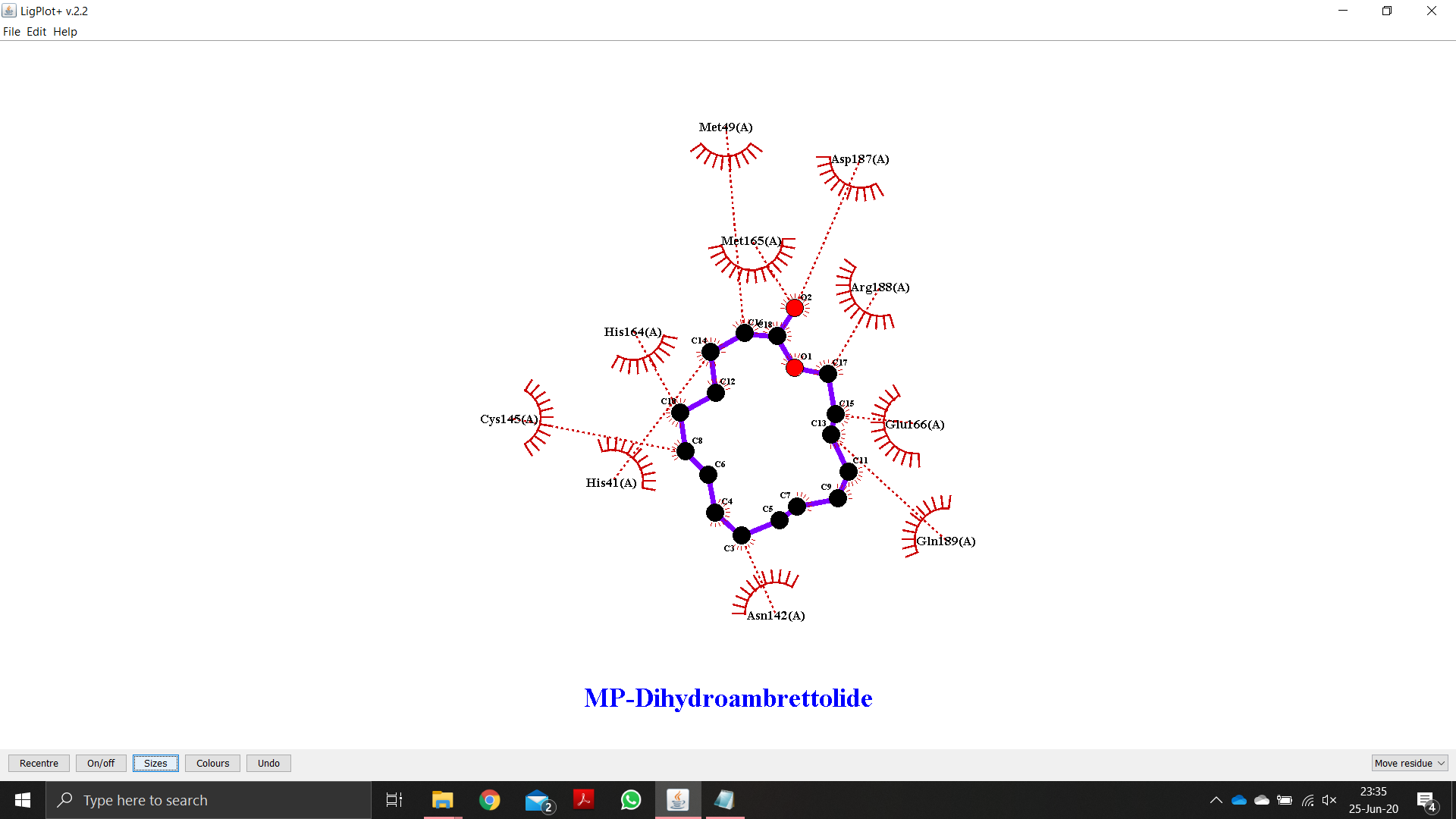 | 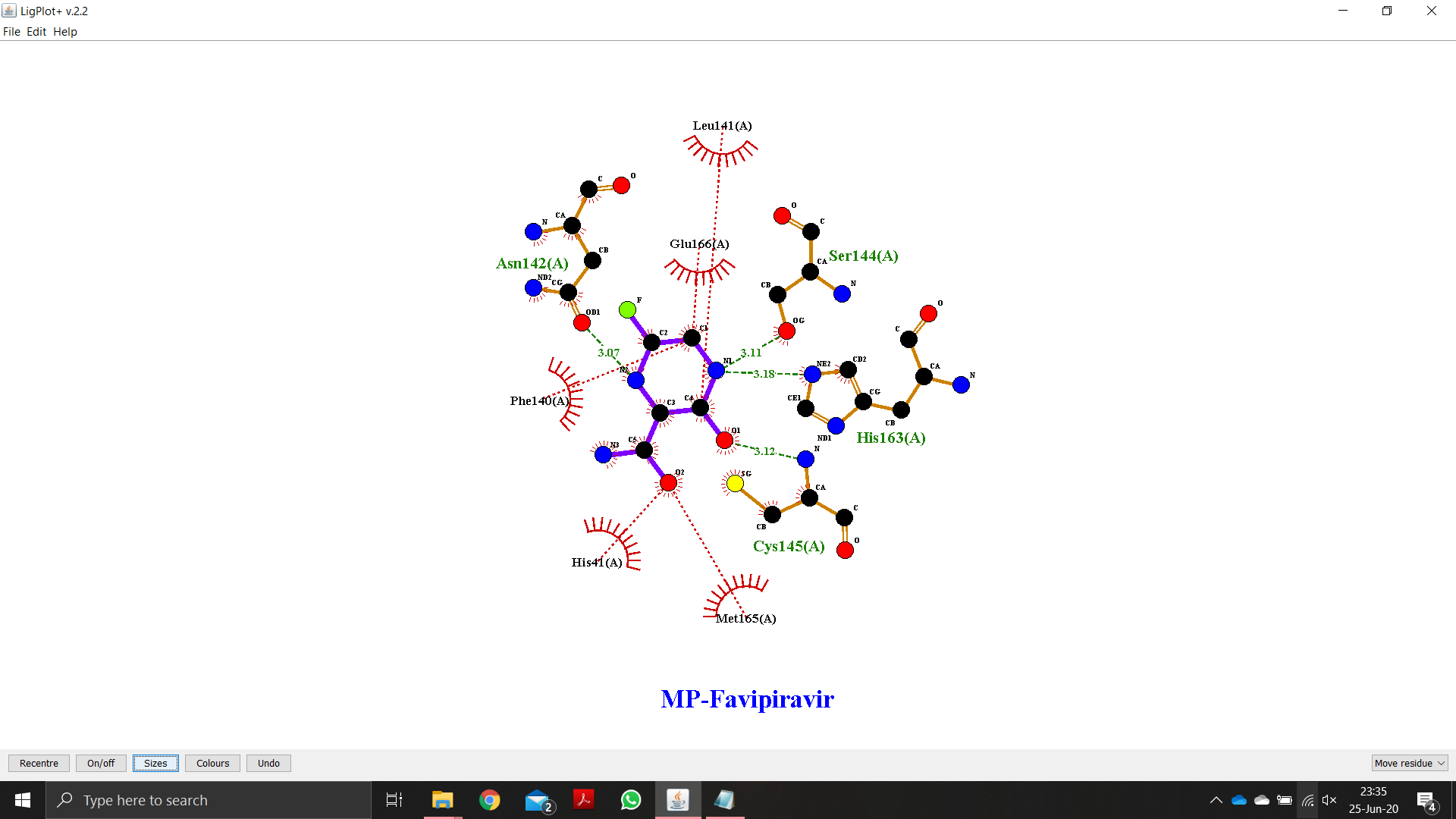 | 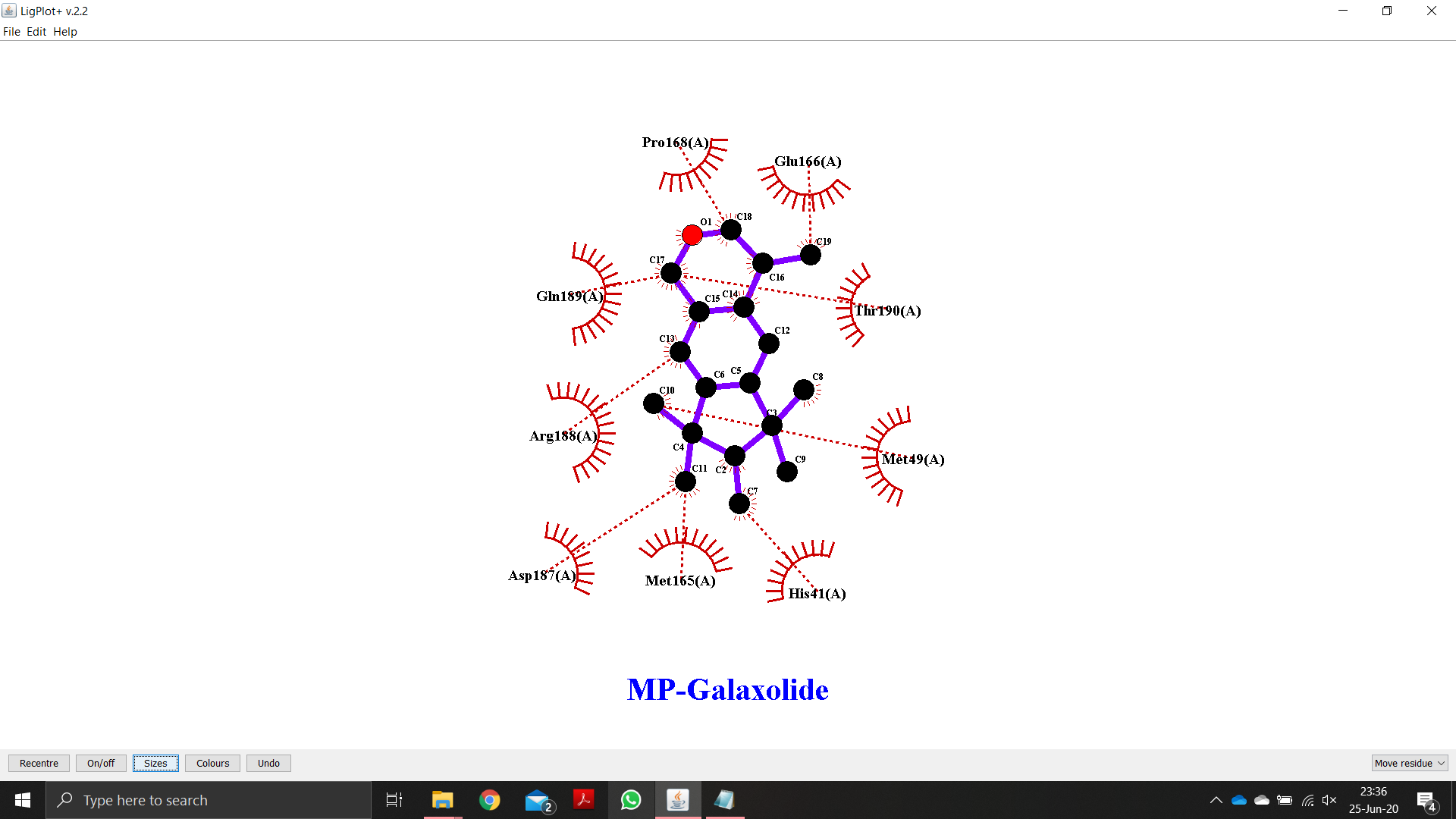 |
| 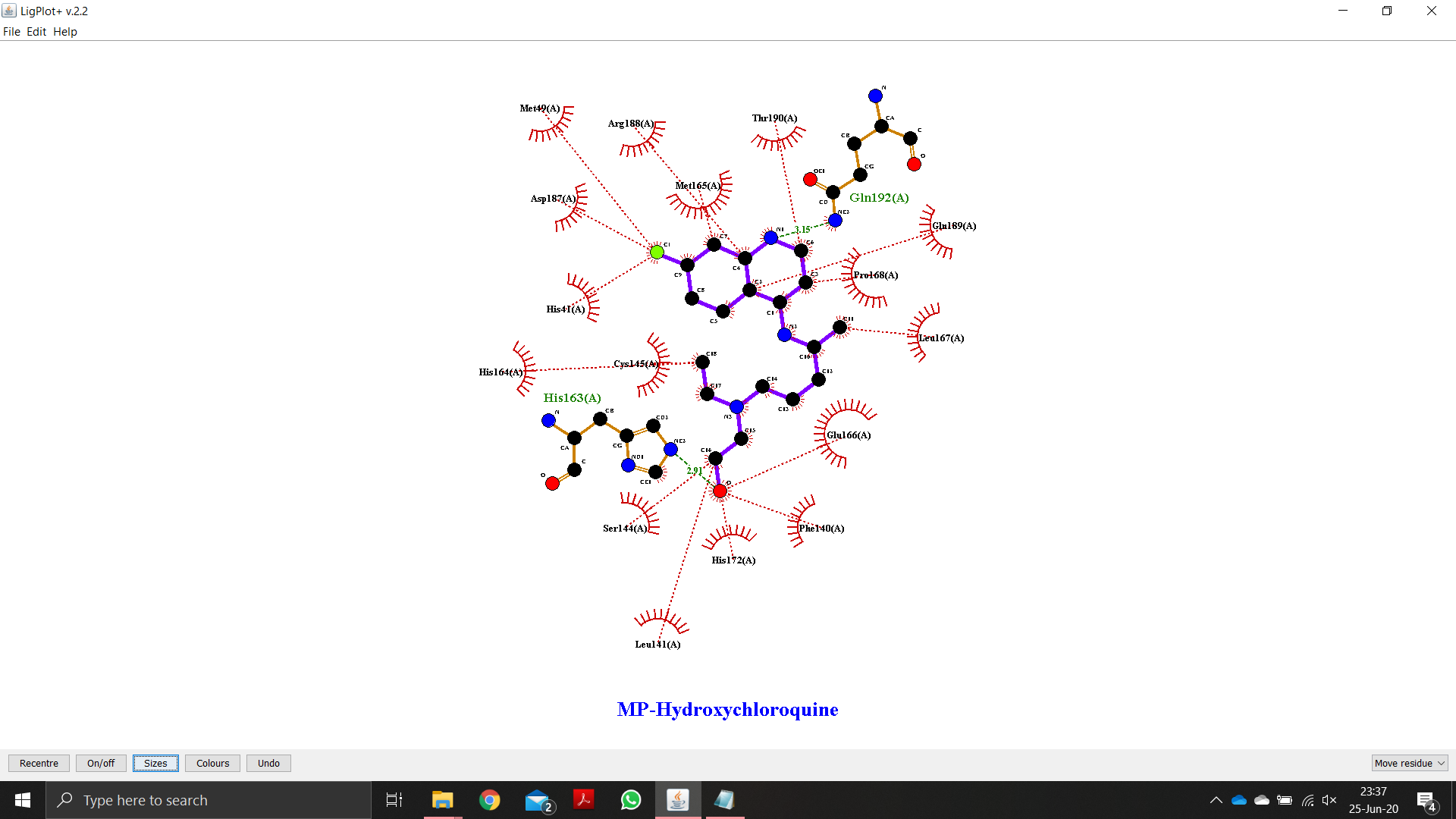 | 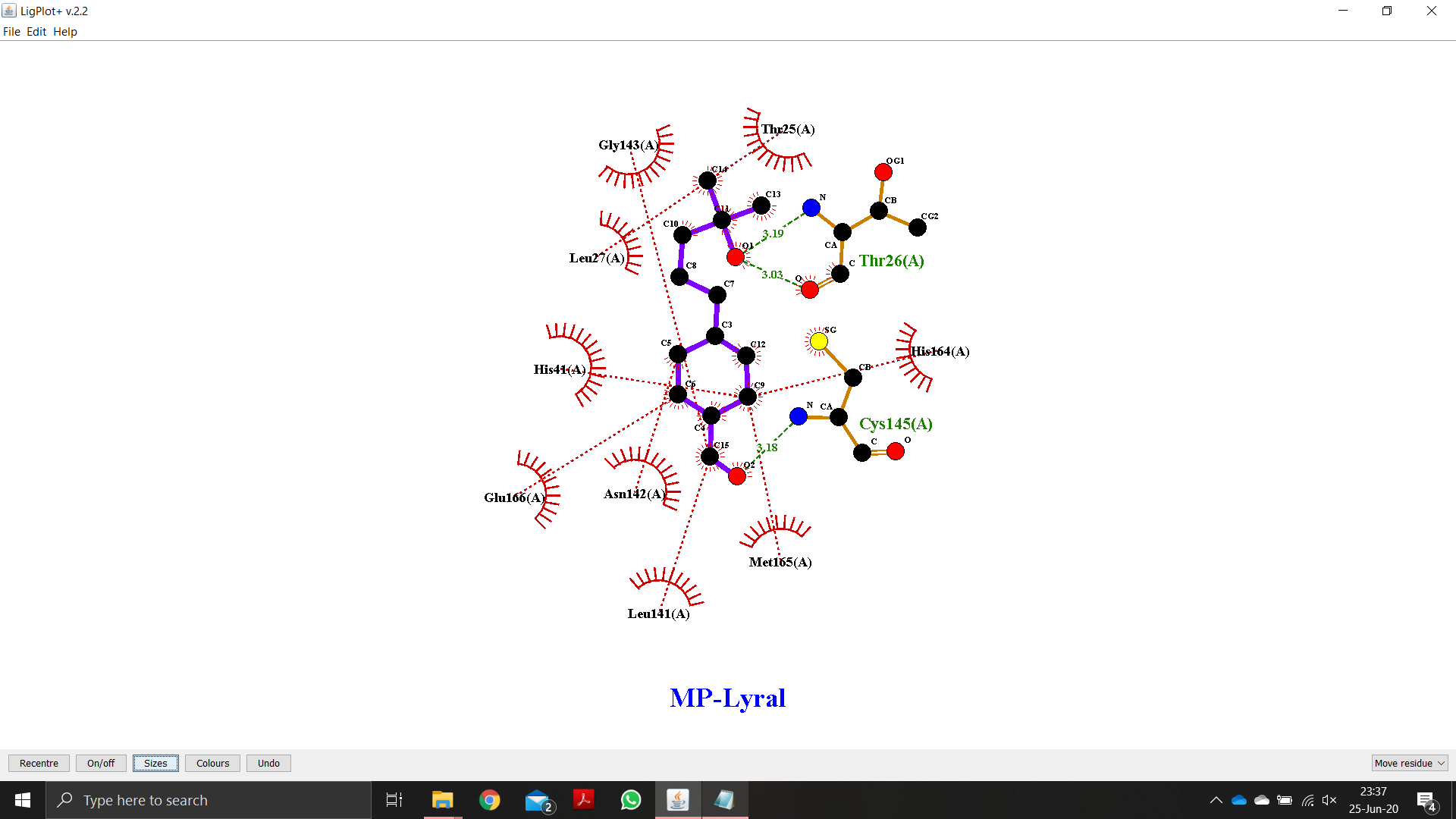 | 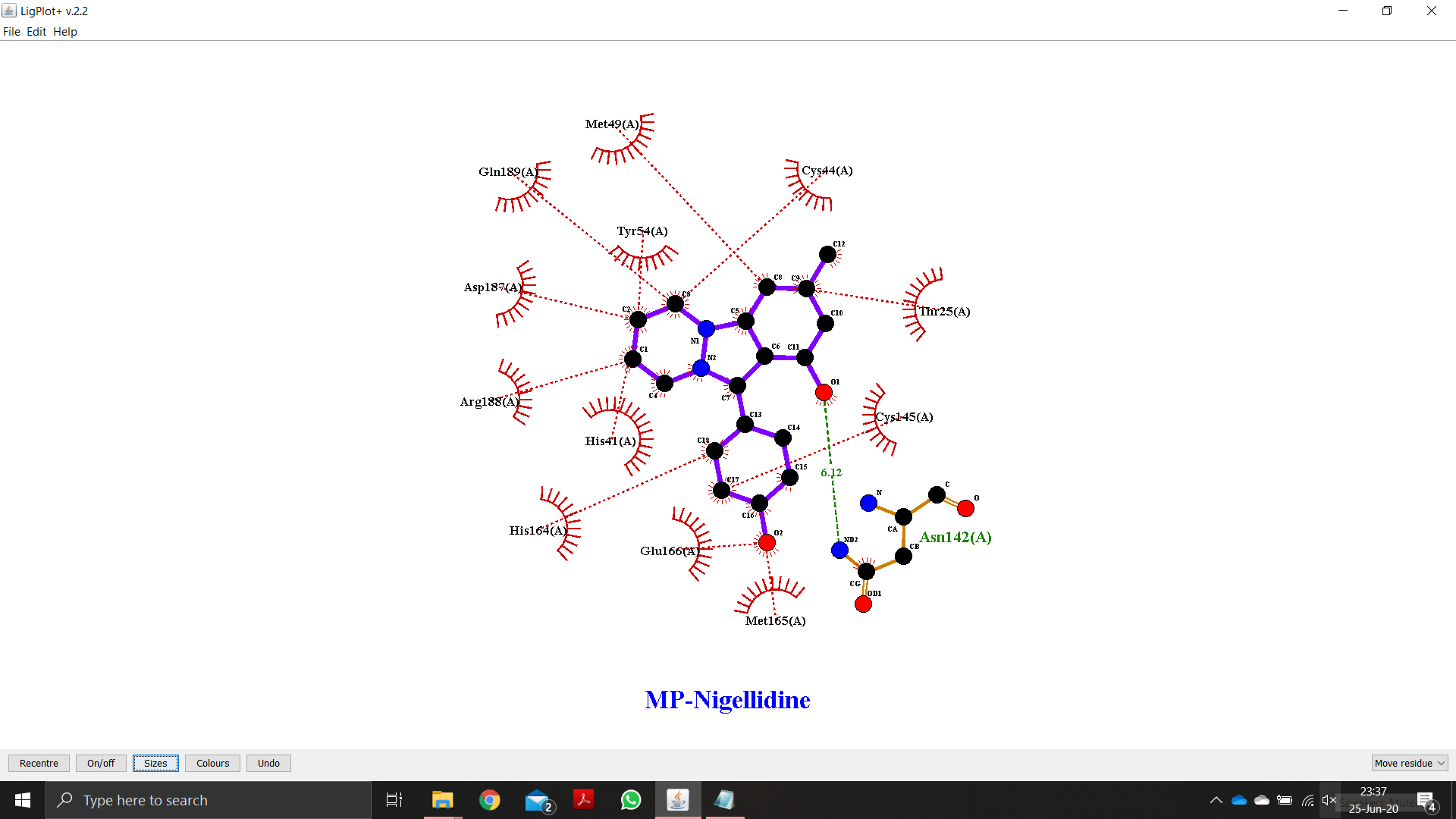 |
| 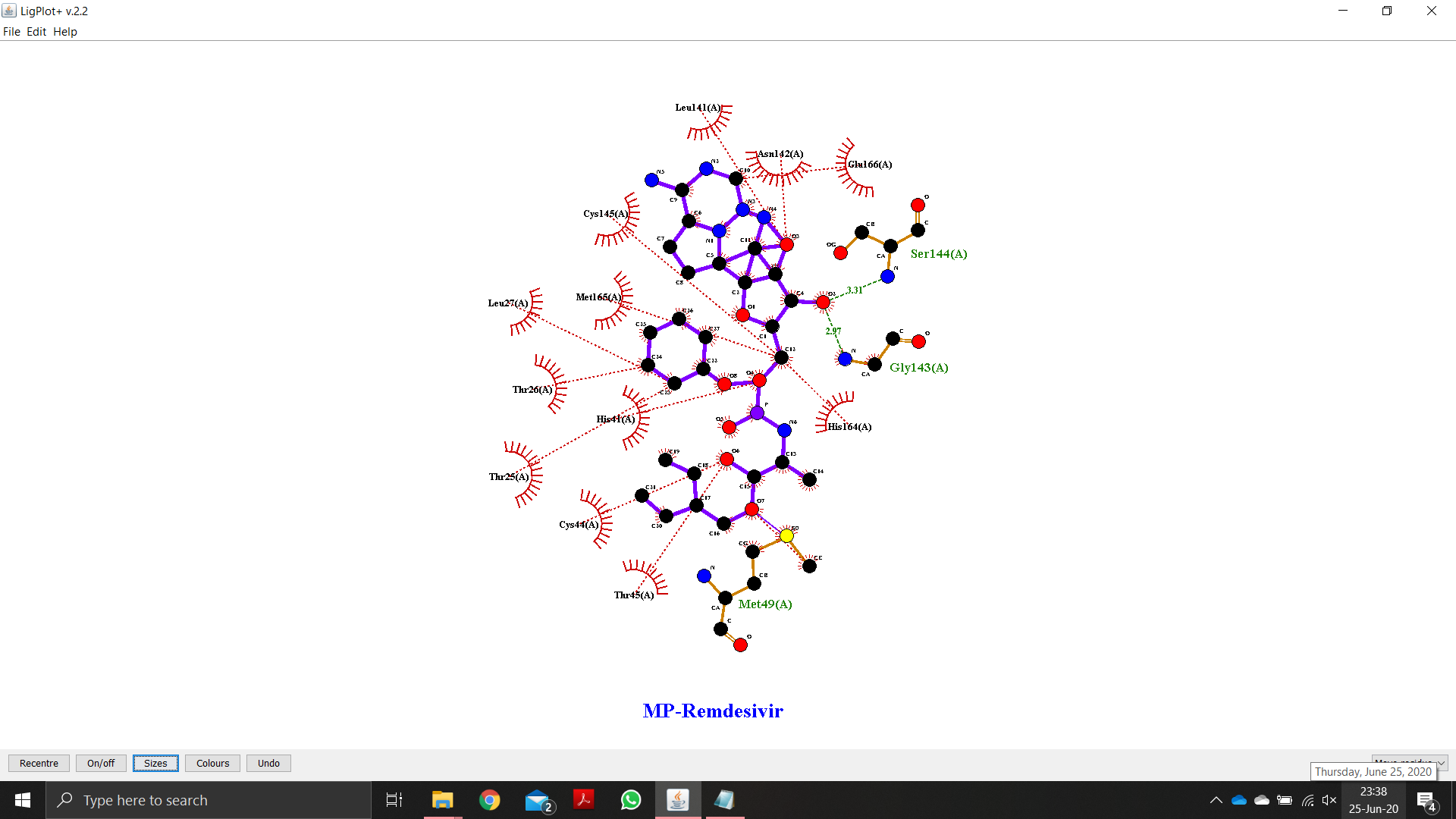 | 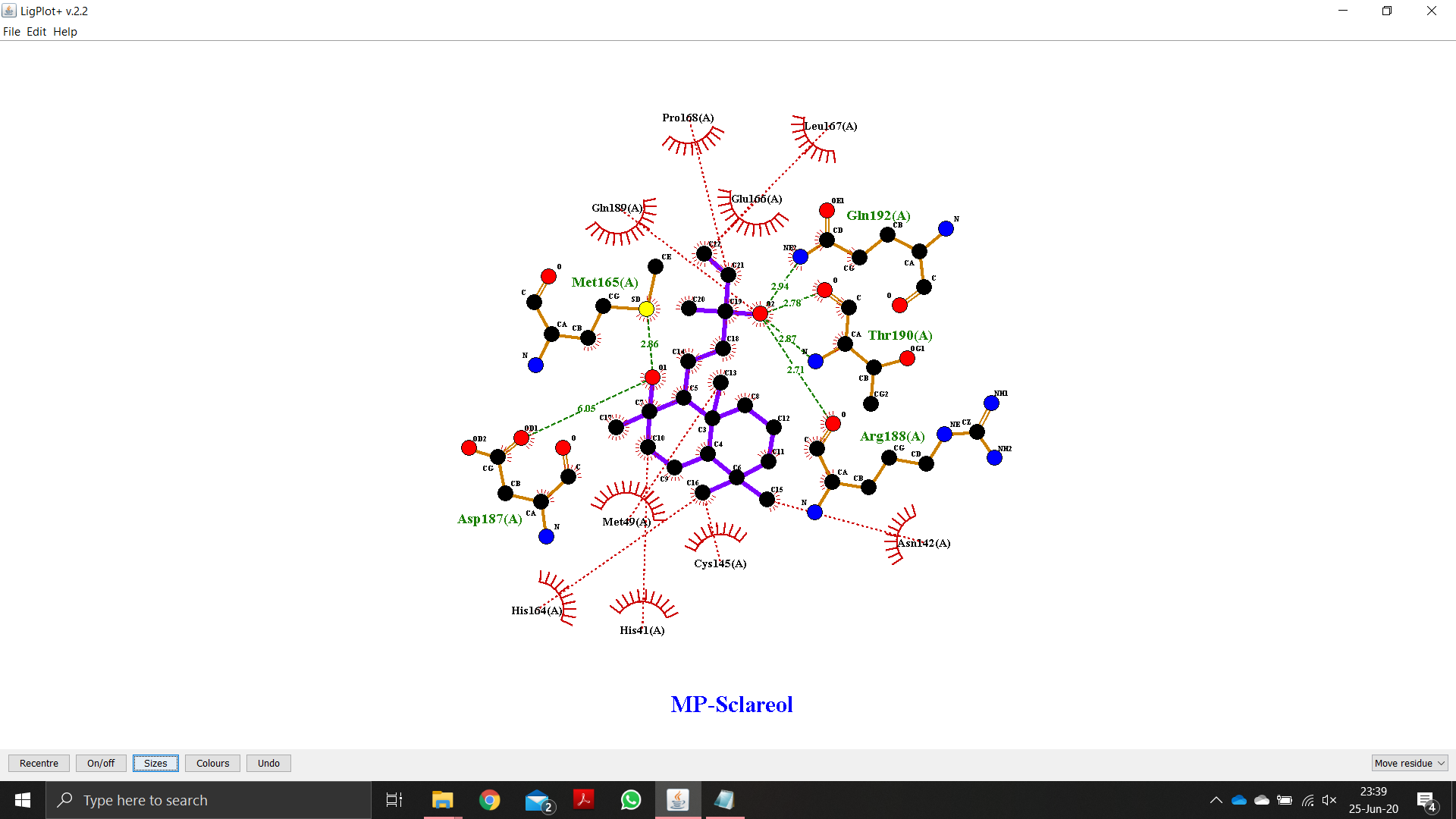 | 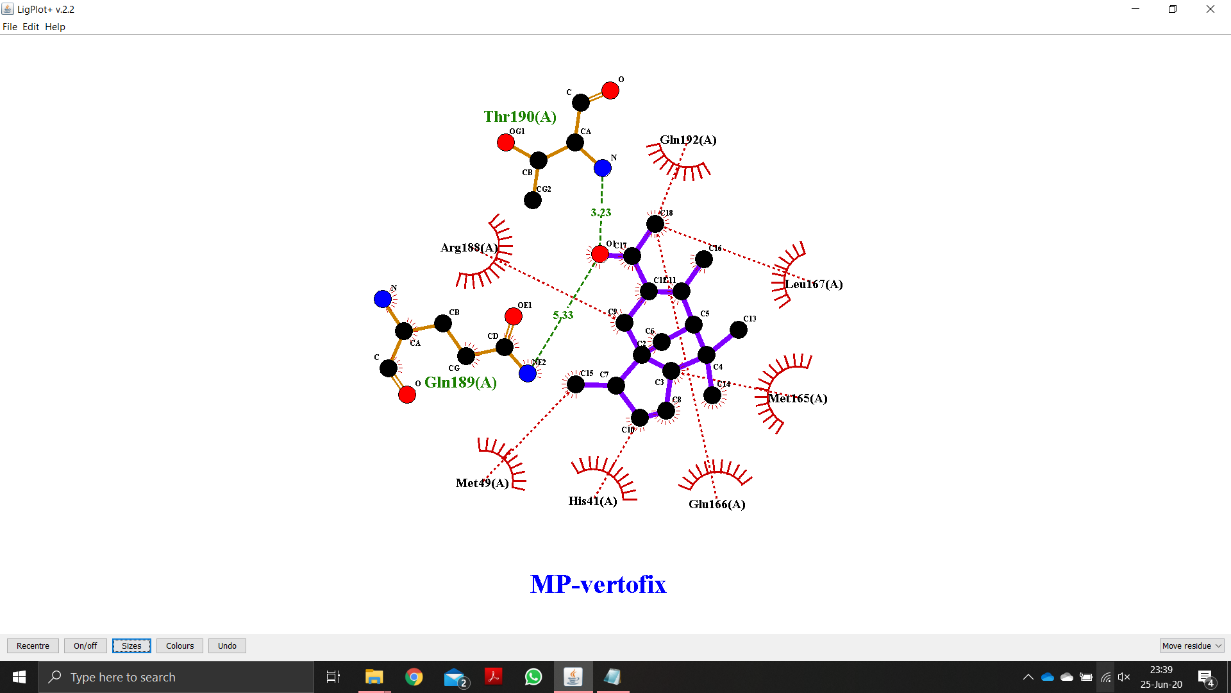 |

**SF3: Figures AutoDock poses for BRD2.**

**
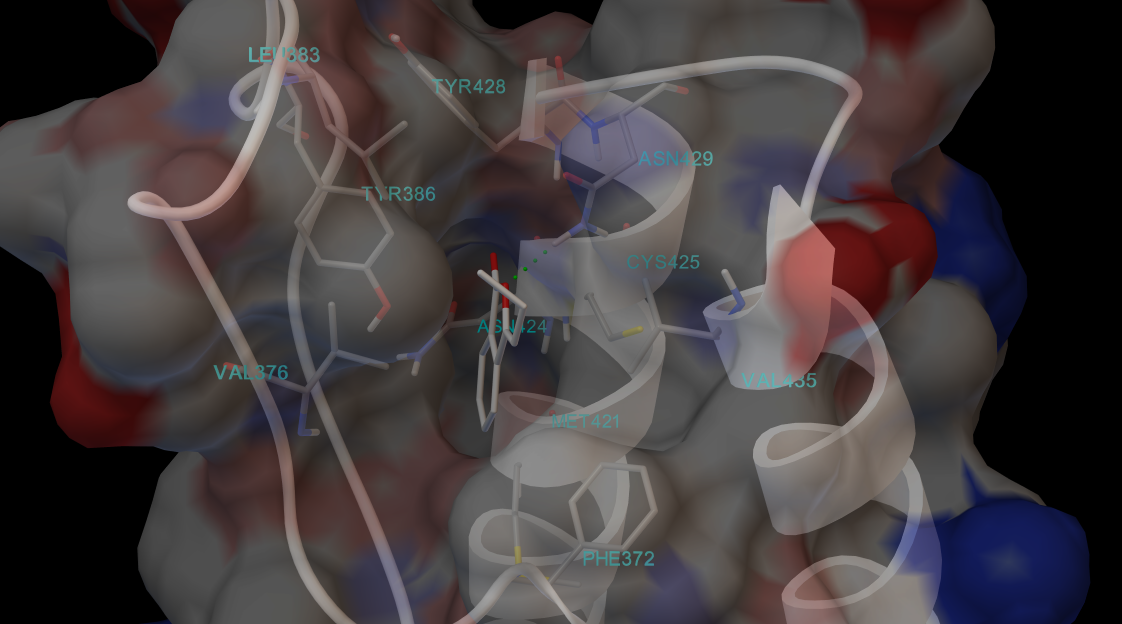

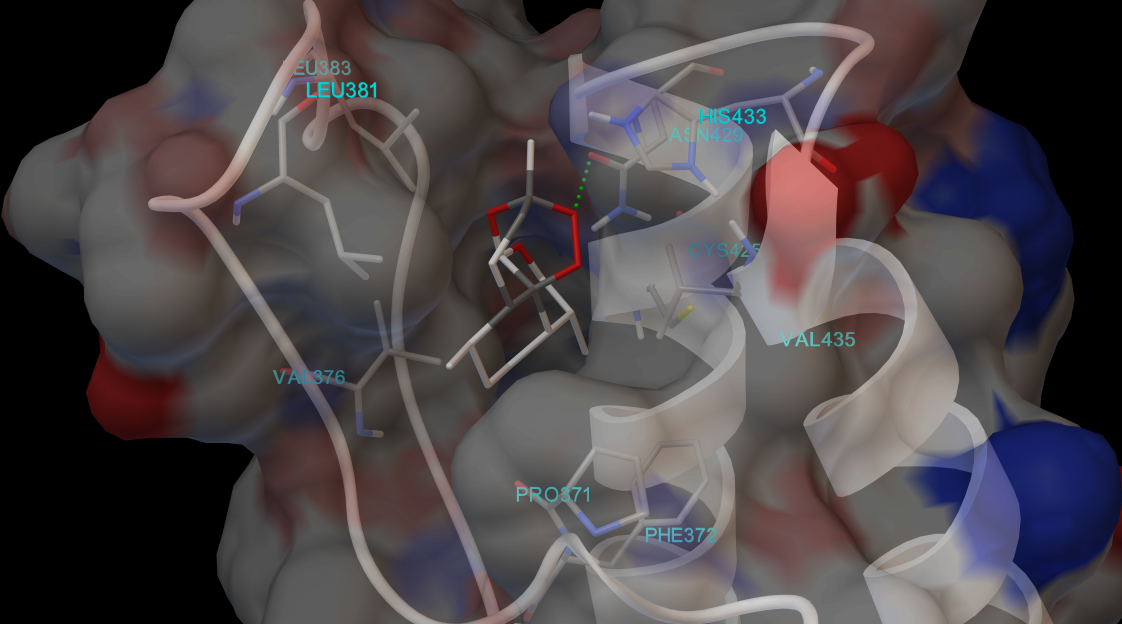

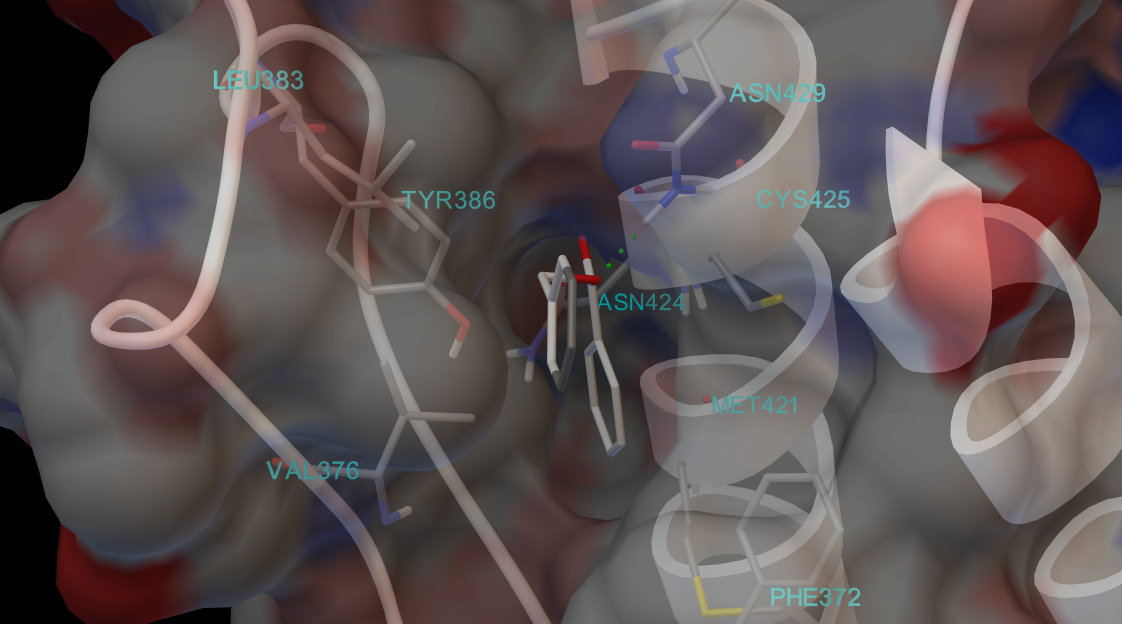

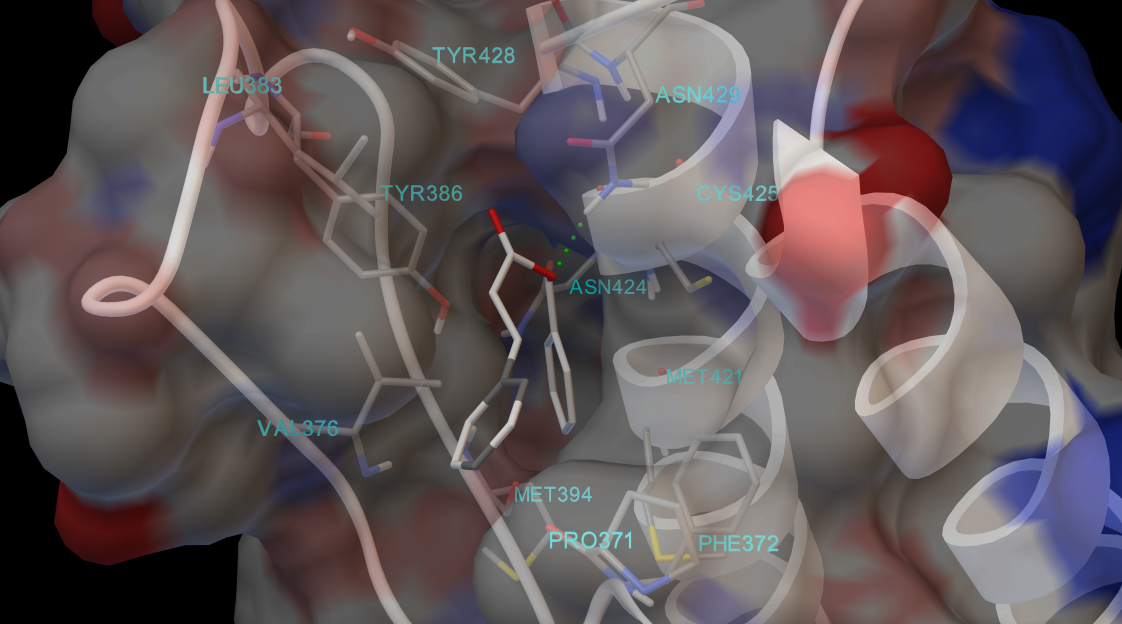

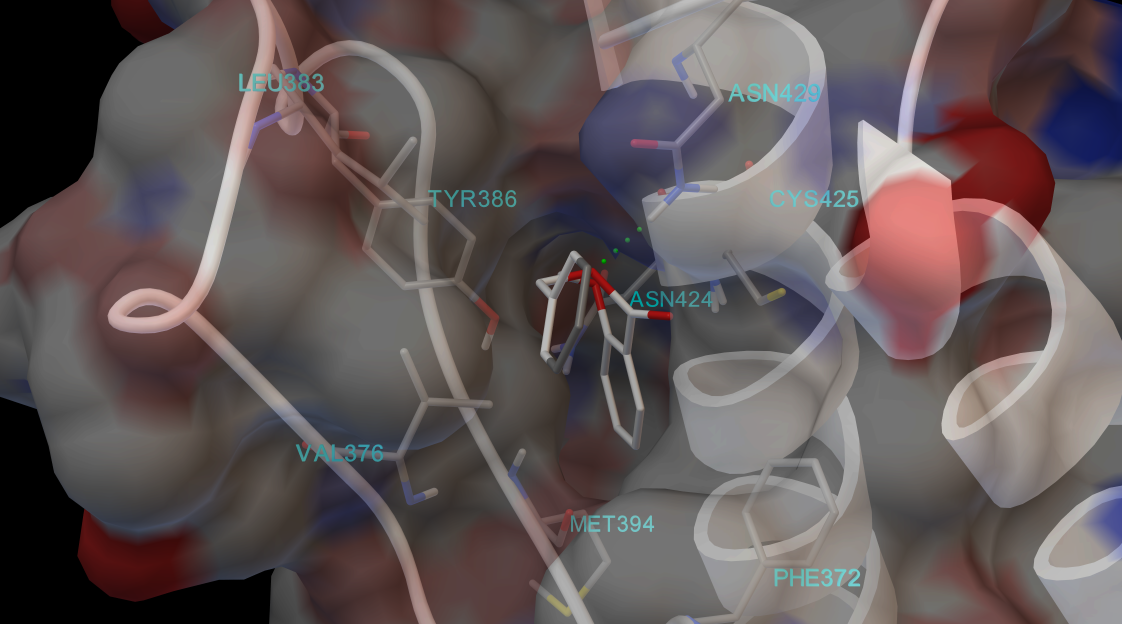

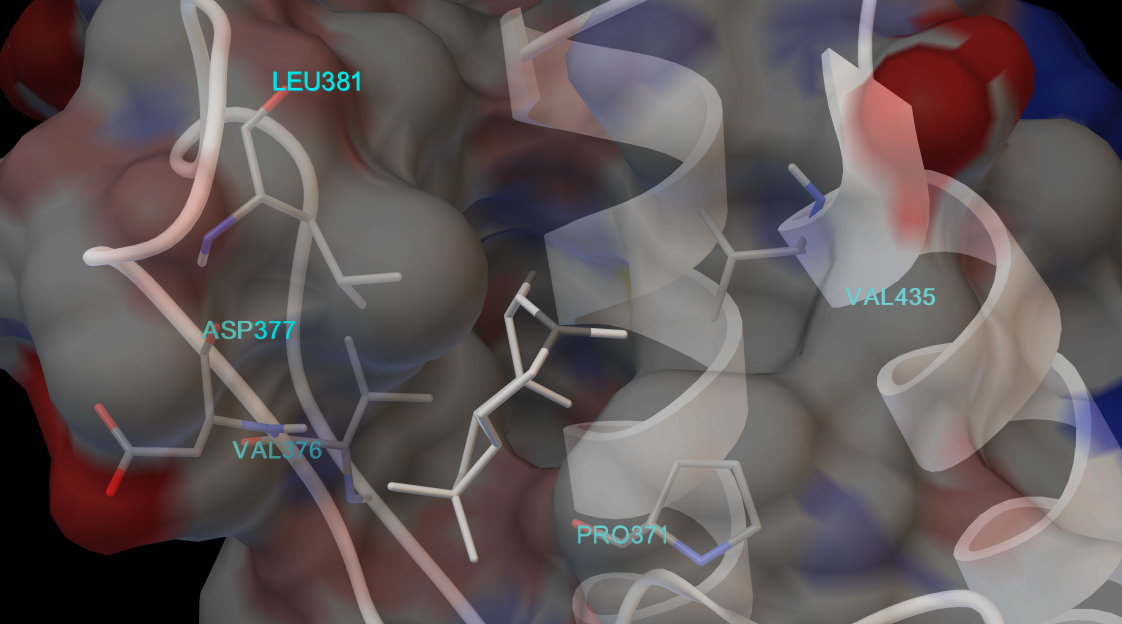

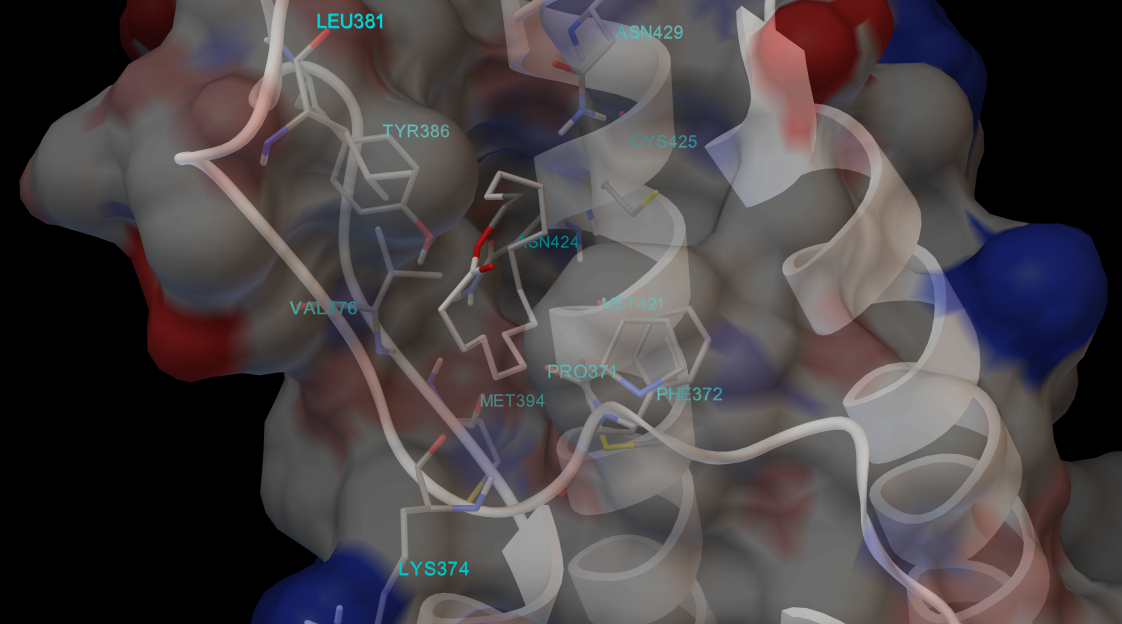

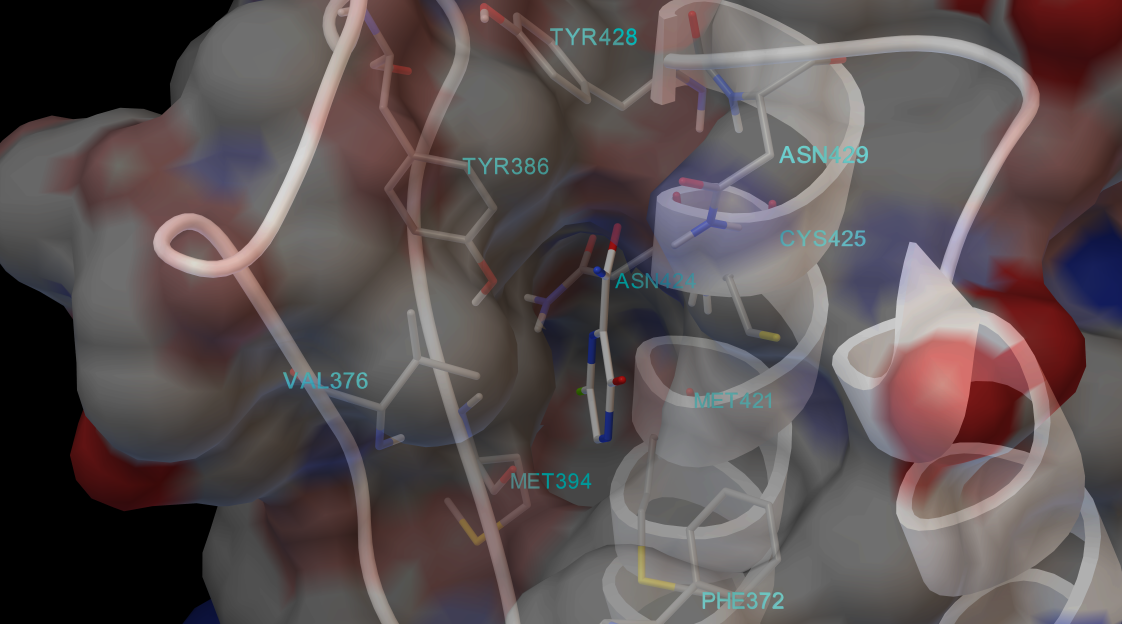

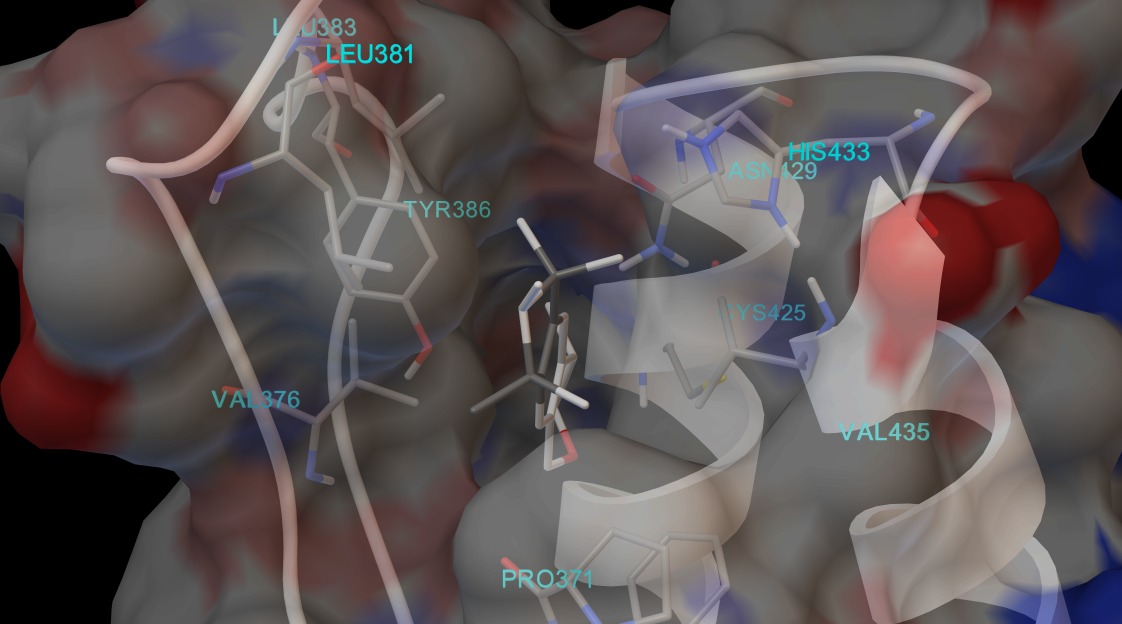

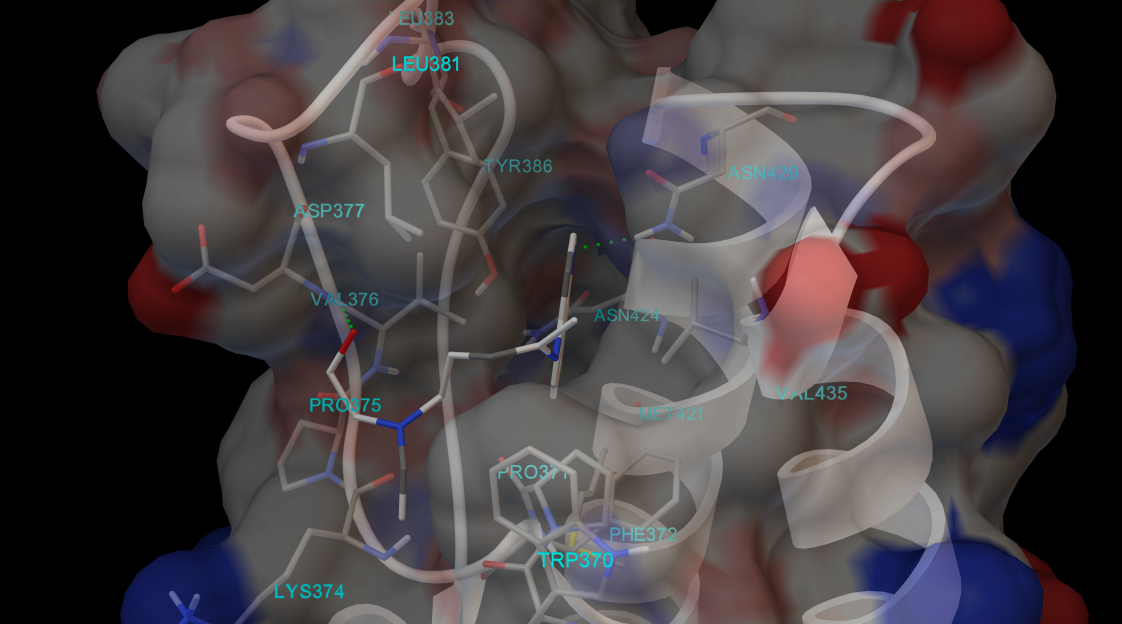

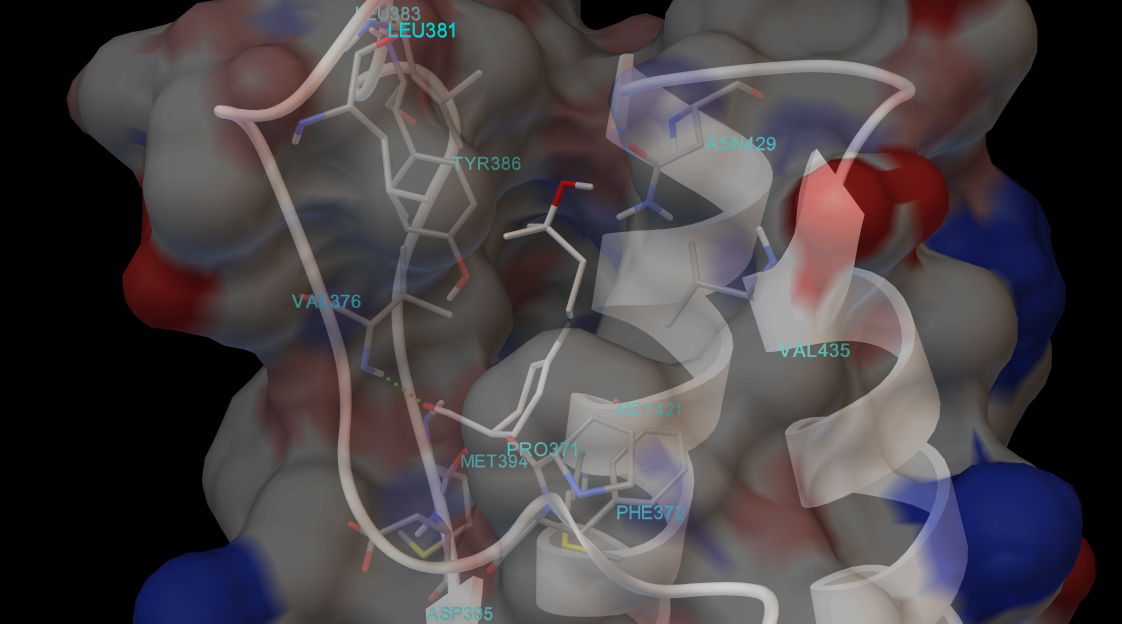

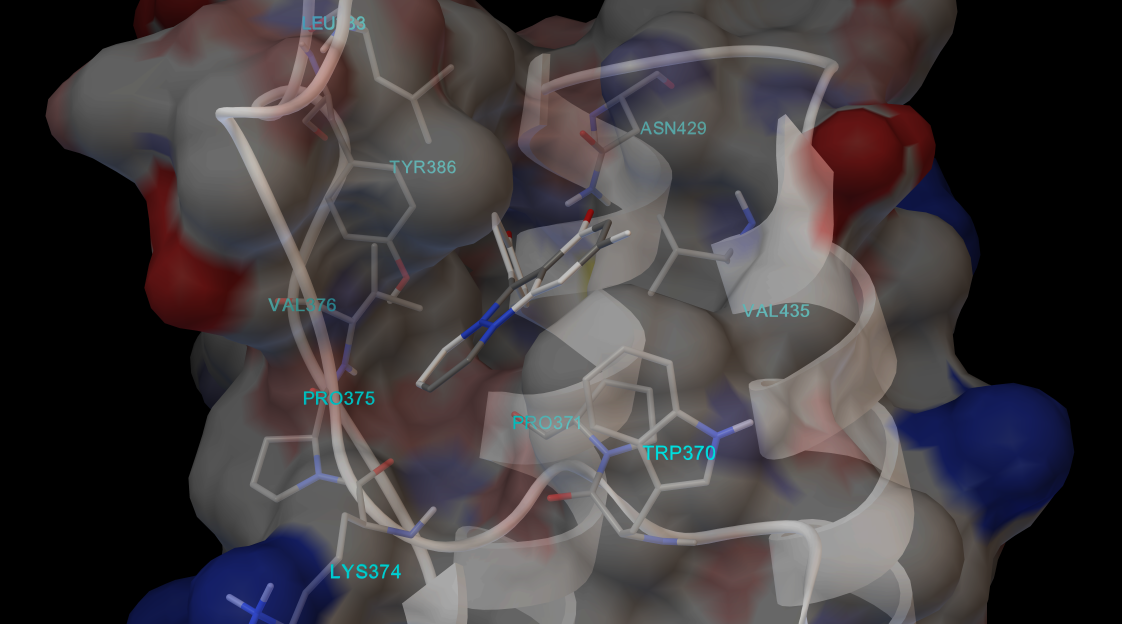

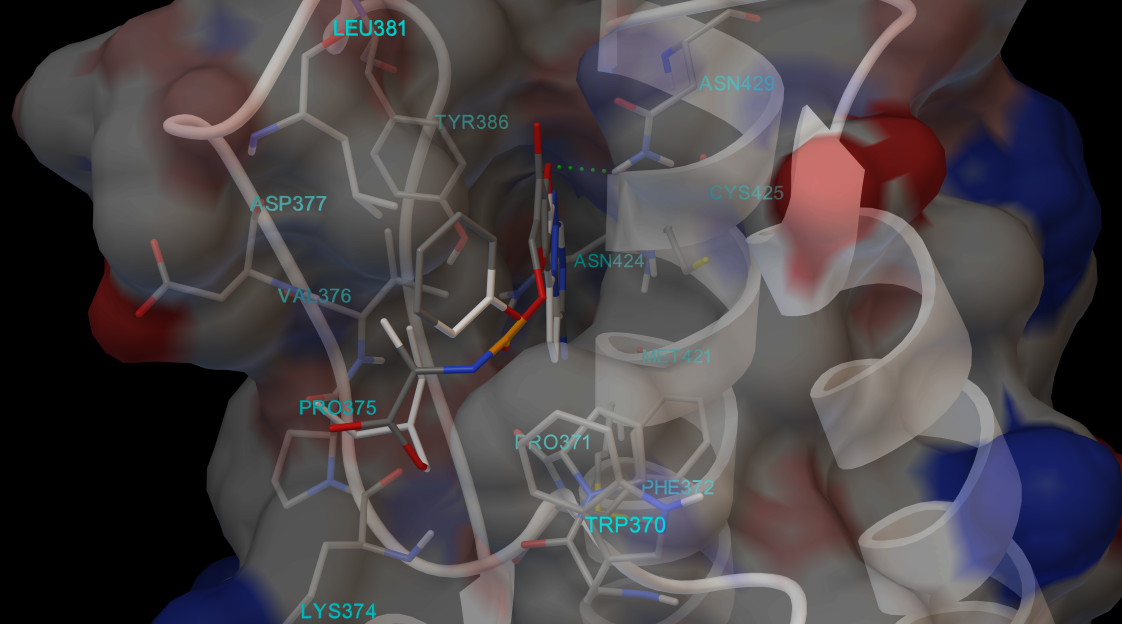

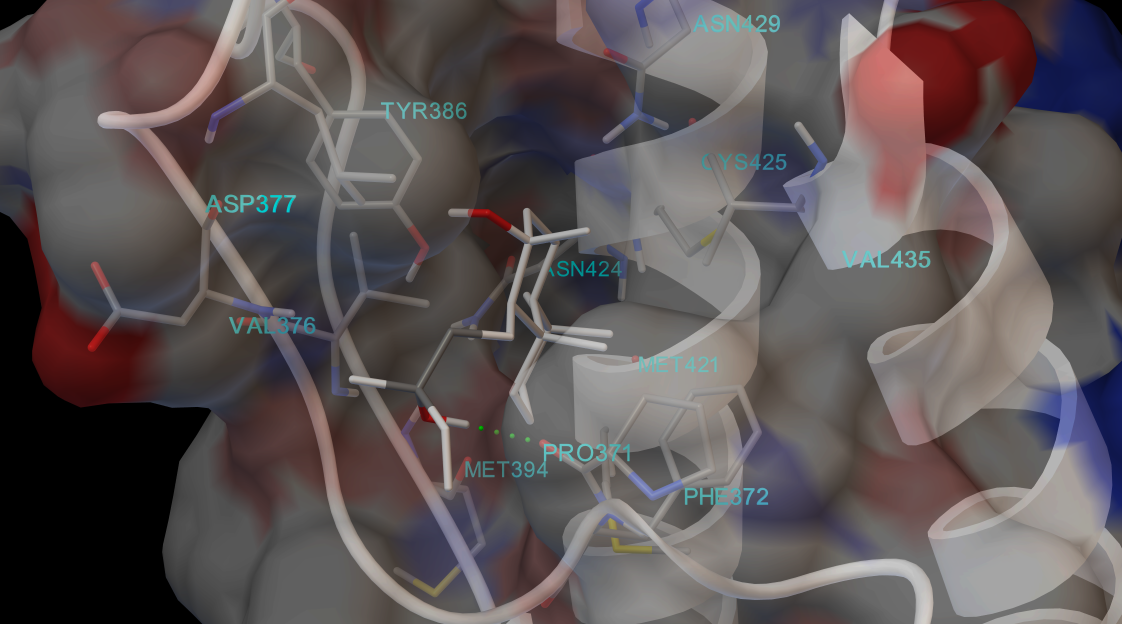

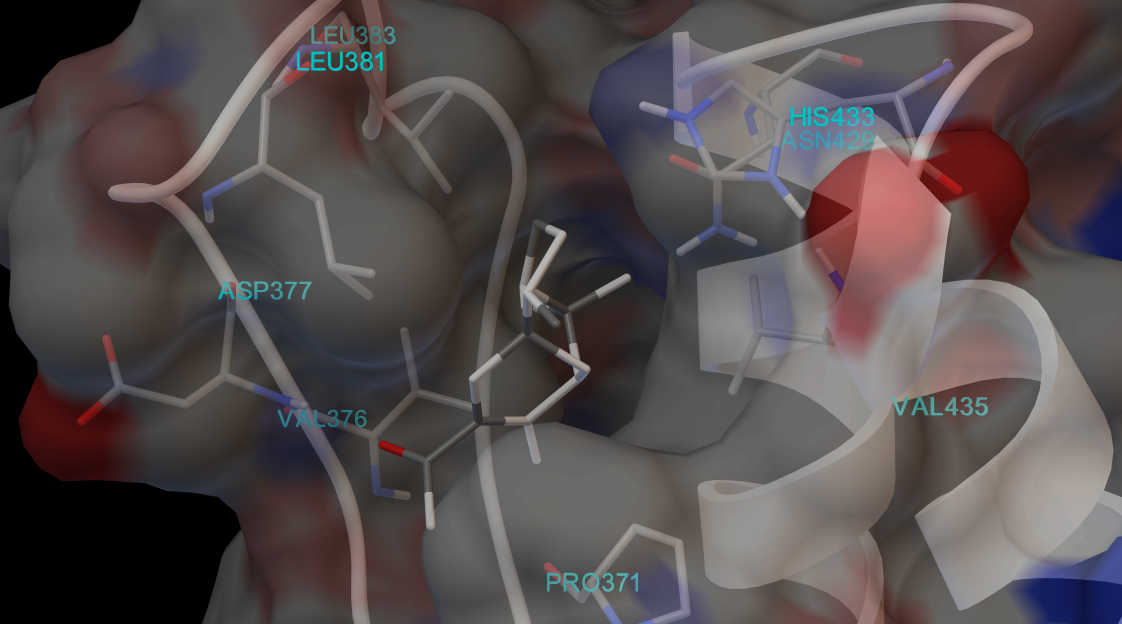
**

Artemisinin

3-PROPYLIDENE PHTHALIDE

BENZYL CINNAMATE

BENZYL BENZOATE

BENZYL SALICYLATE

BETA CARYOPHYLLENE

DIHYDROAMBRETTOLIDE

Favipiravir

Hydroxychloroquine

GALAXOLIDE

LYRAL

Nigellidine

SCLAREOL

Remdesivir

VERTOFIX (ALPHA ACETYL CEDRENE)

**SF4: Figures AutoDock poses for MPro.**

**
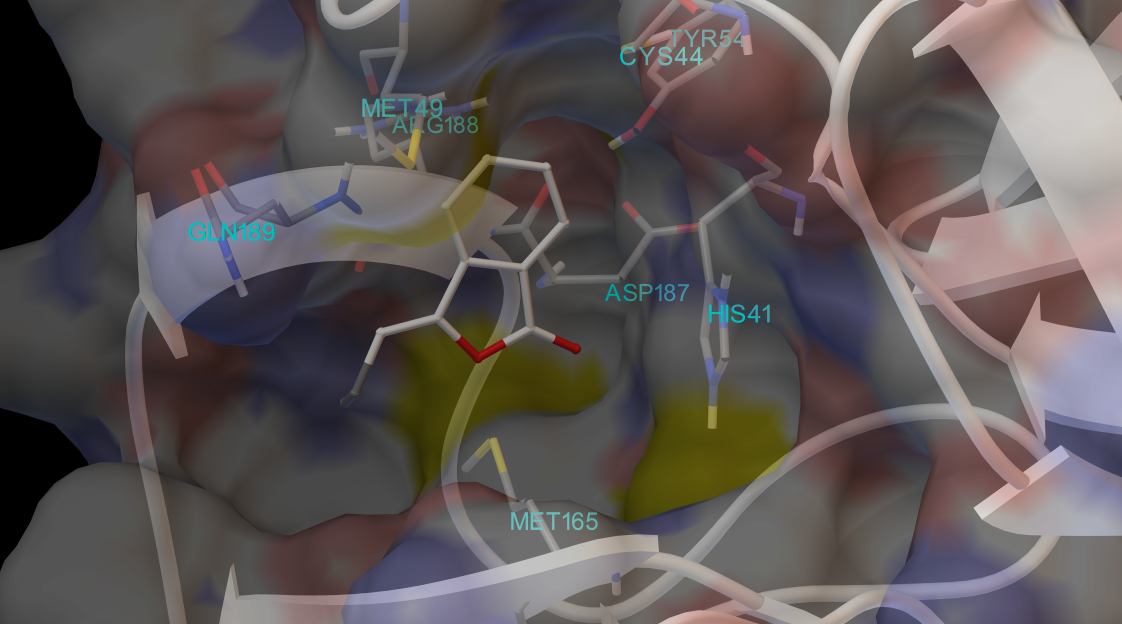

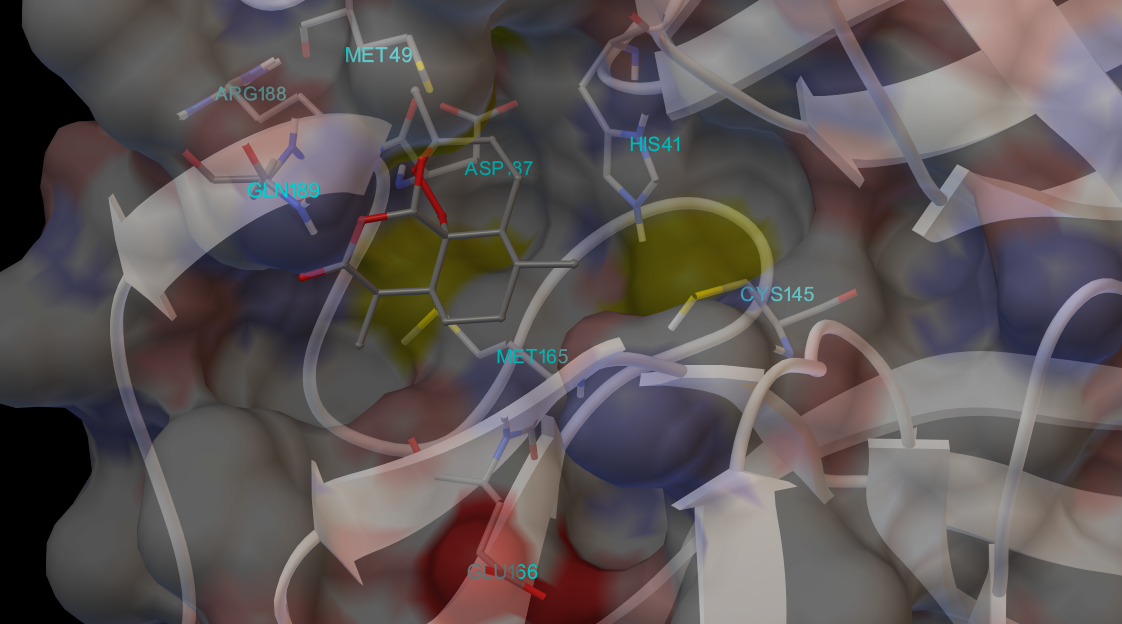

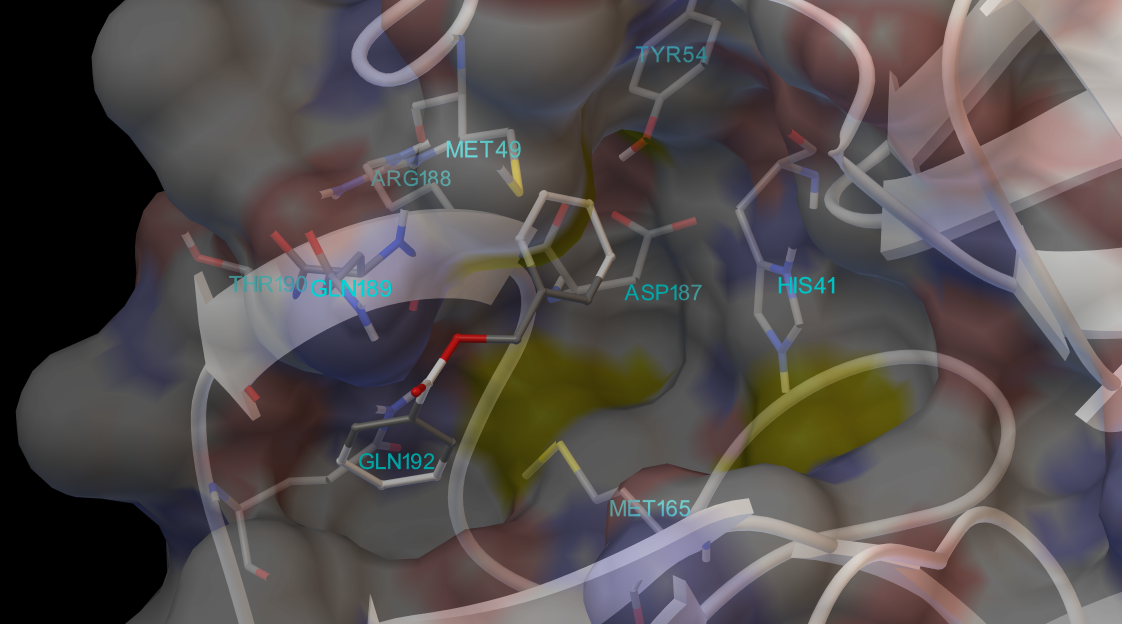

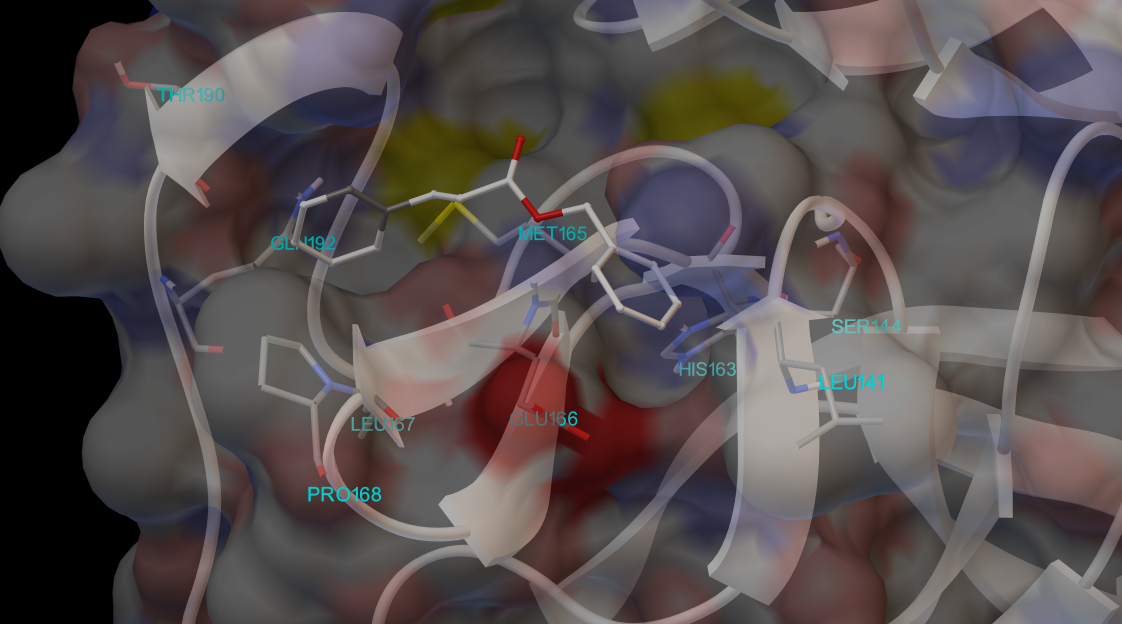

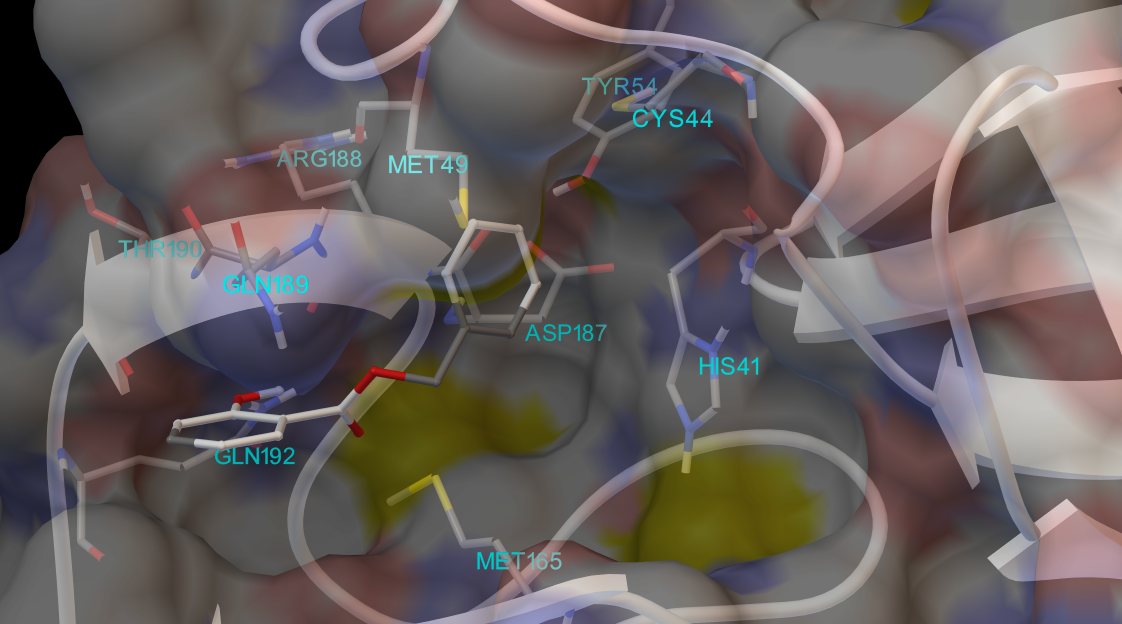

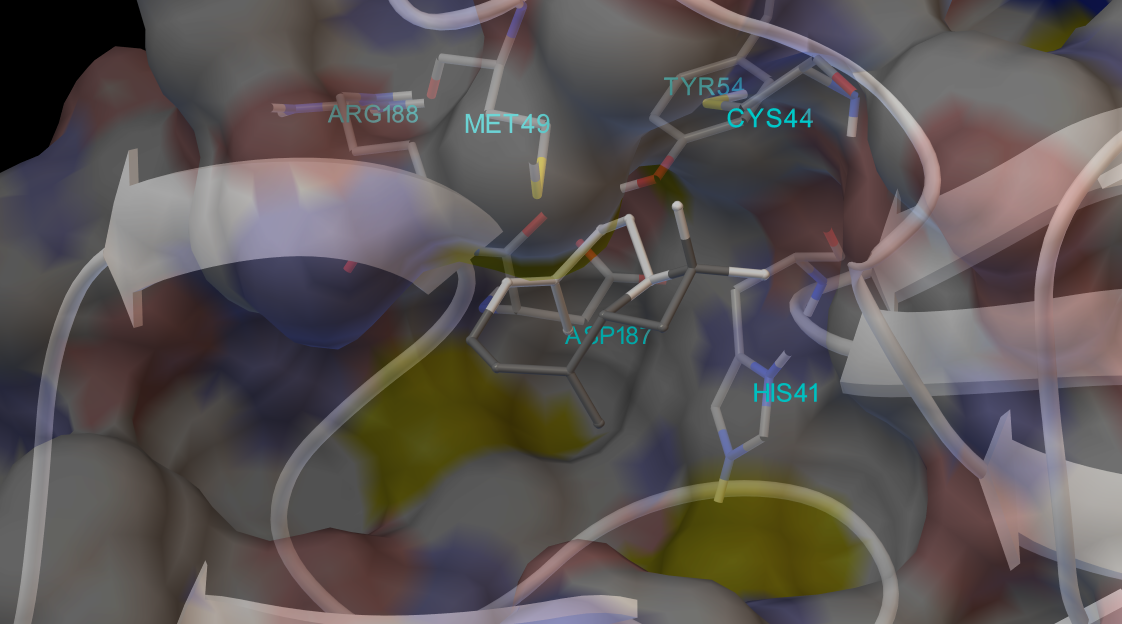

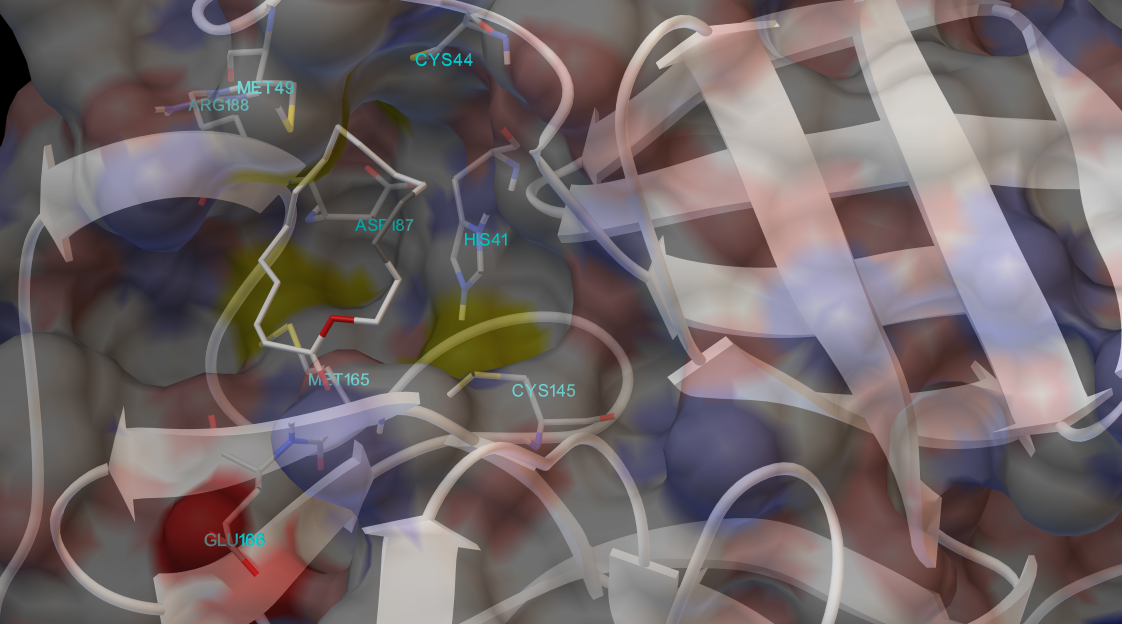

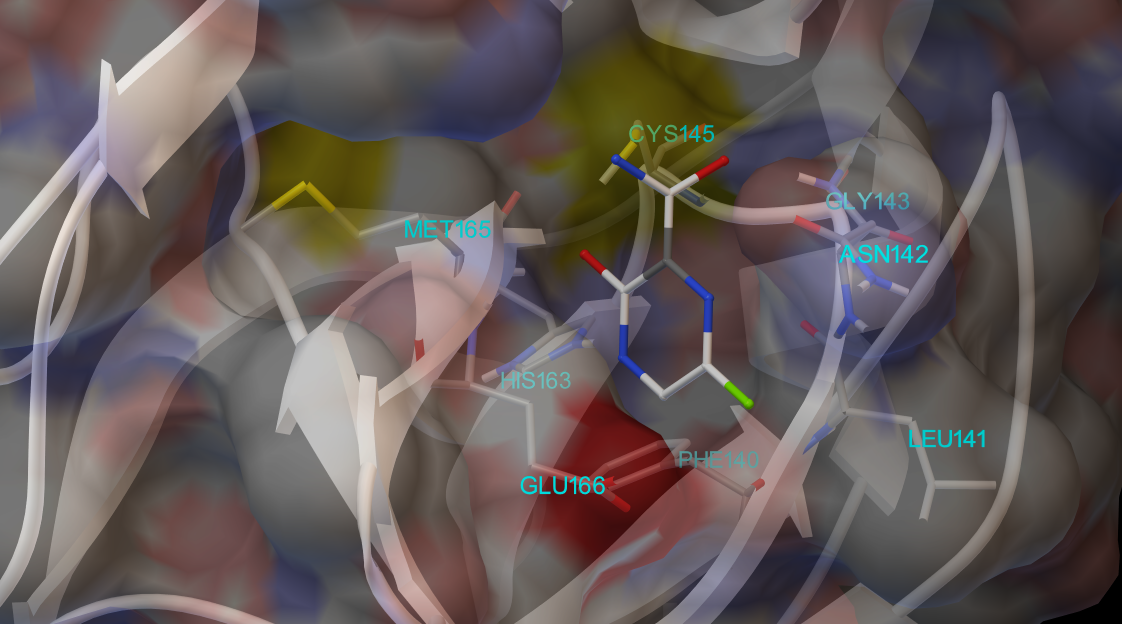

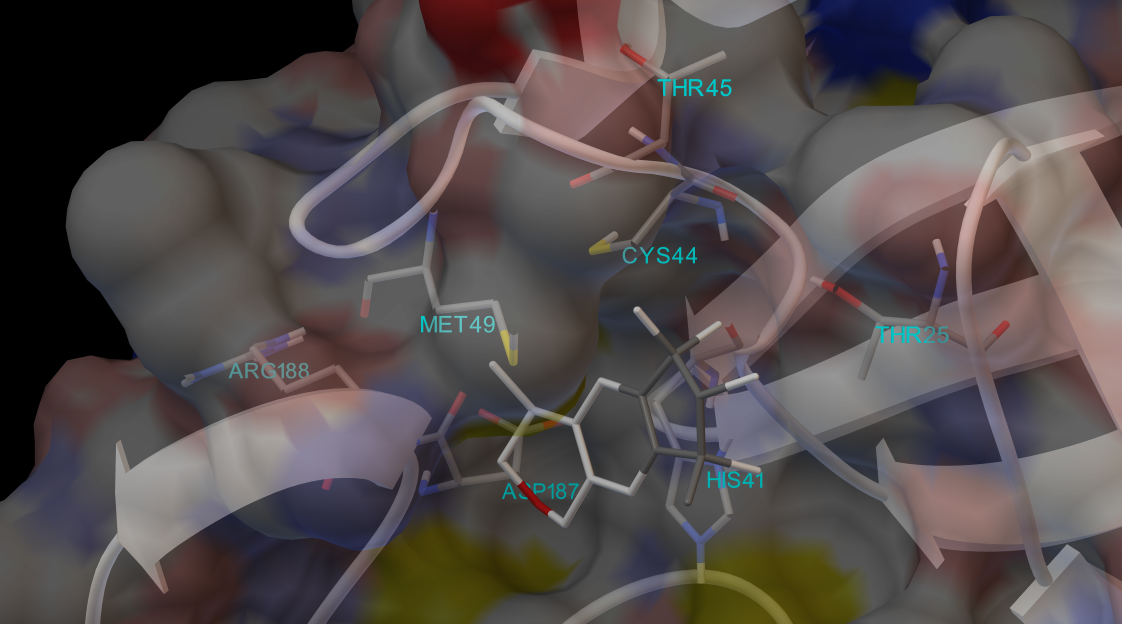

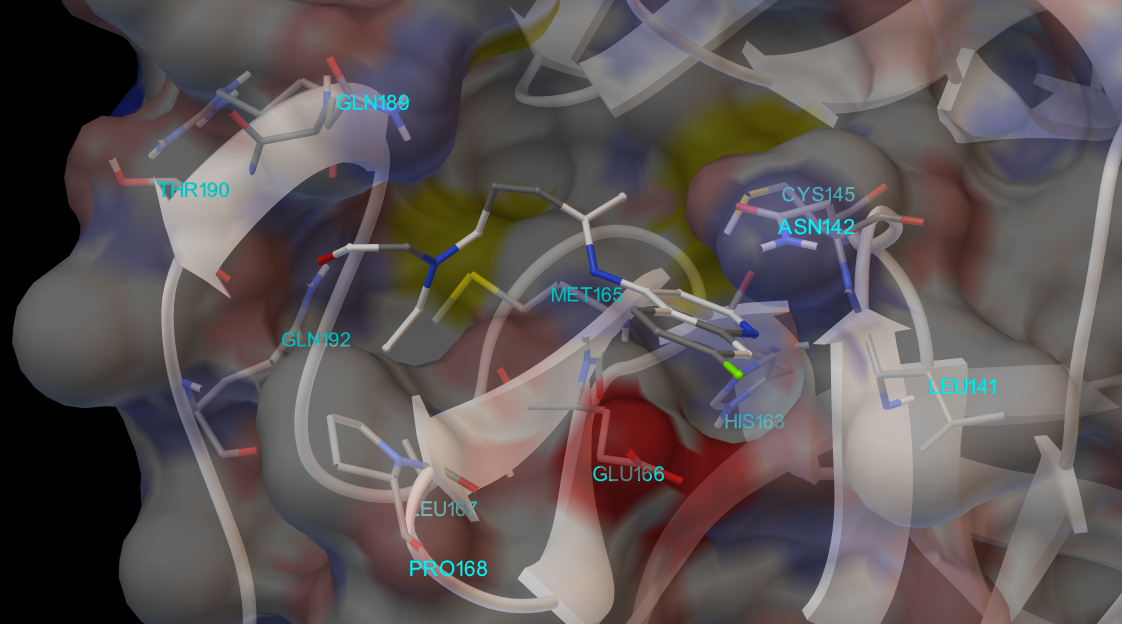

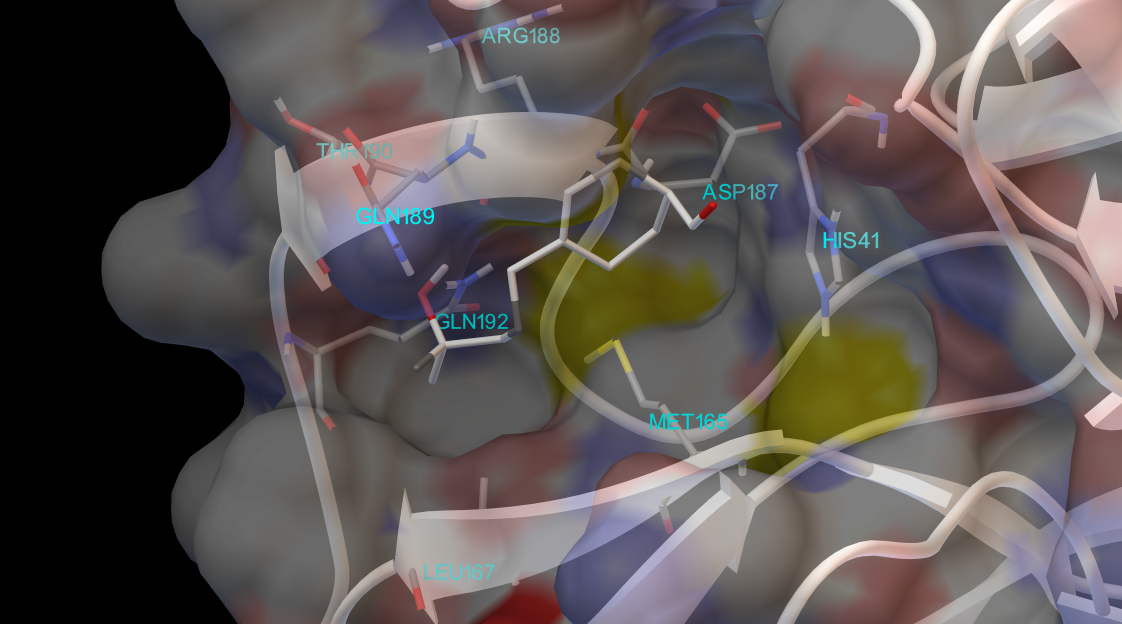

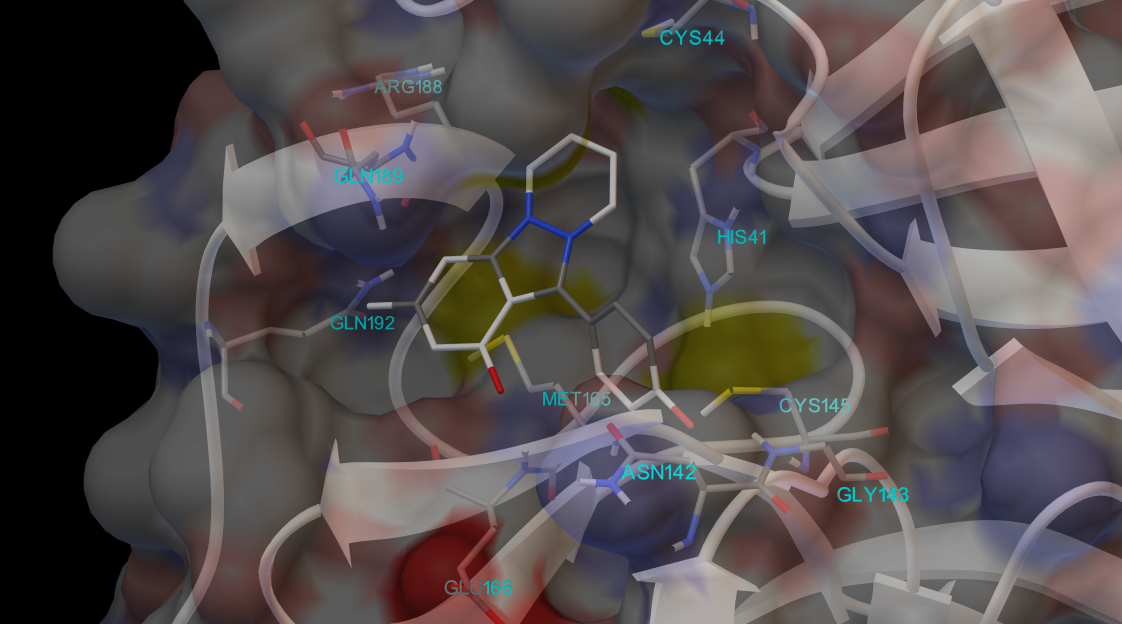

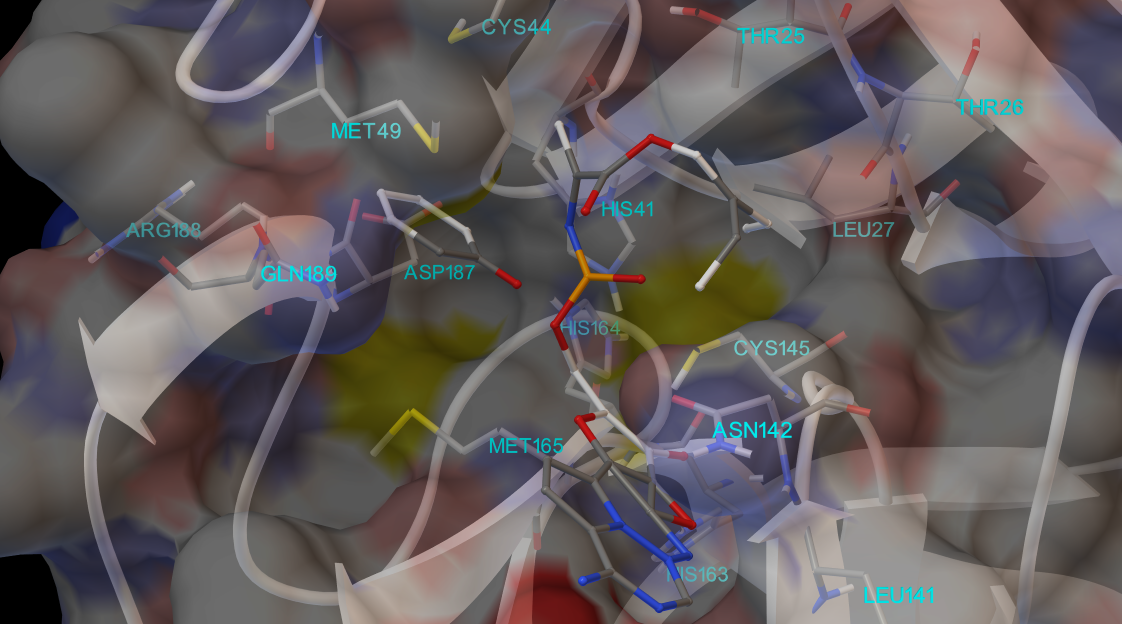

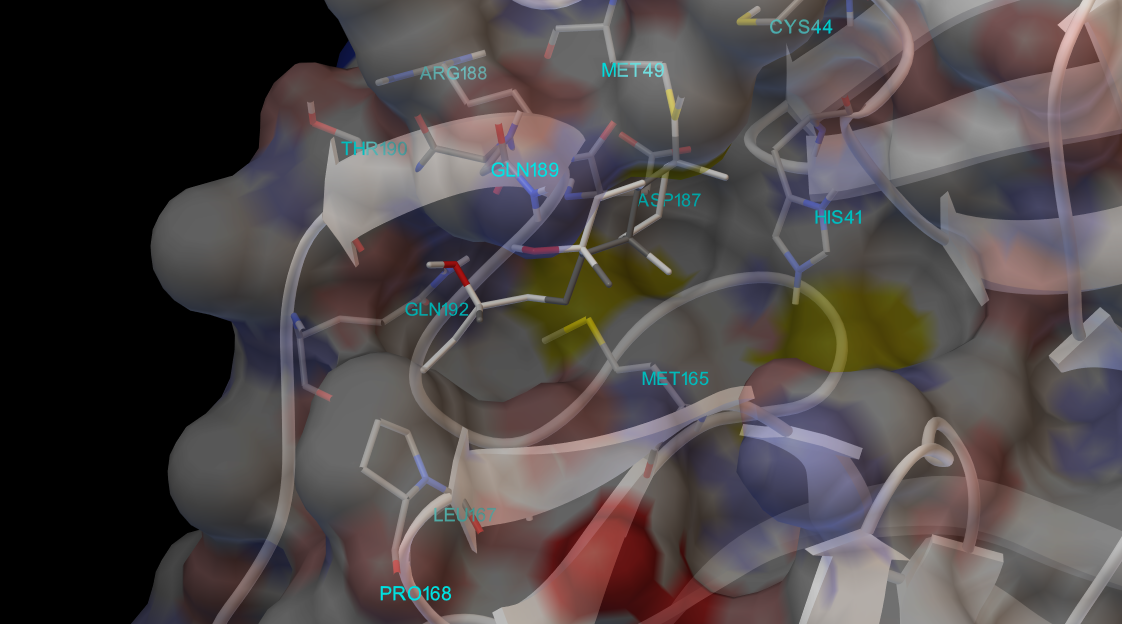

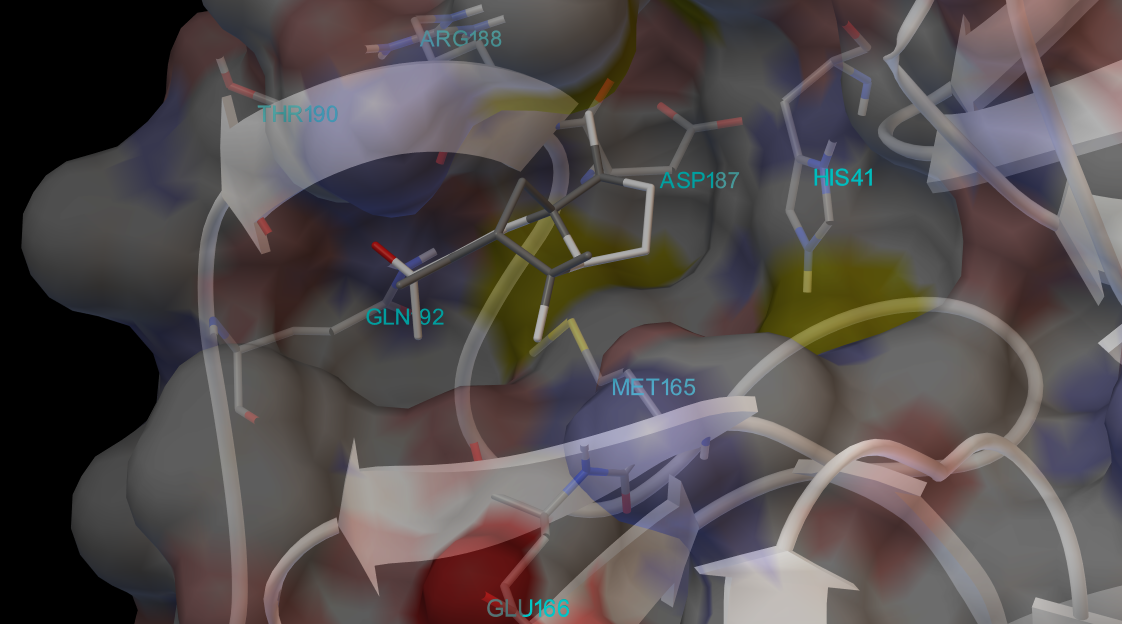
**

Artemisinin

3-PROPYLIDENE PHTHALIDE

BENZYL CINNAMATE

BENZYL BENZOATE

BETA CARYOPHYLLENE

BENZYL SALICYLATE

Favipiravir

DIHYDROAMBRETTOLIDE

GALAXOLIDE

Hydroxychloroquine

LYRAL

Nigellidine

Remdesivir

SCLAREOL

VERTOFIX (ALPHA ACETYL CEDRENE)

**Table S1:** Mean binding affinity (kcal/mol) calculated by Vina. Selected top 9 compounds in this study, previously reported compounds, and binding affinities ≤ -6 kcal/mol were printed in bold letters.

|  | BRD2 | | Main Protease | | Nucleocapsid | | Receptor Bimding Domain | |
| --- | --- | --- | --- | --- | --- | --- | --- | --- |
|  | Mean | SEM | Mean | SEM | Mean | SEM | Mean | SEM |
| **3-PROPYLIDENE PHTHALIDE** | **-6.50** | 0.00 | -5.43 | 0.02 | -5.83 | 0.03 | -5.18 | 0.03 |
| ACETYL ISOEUGENOL | **-6.30** | 0.00 | -5.50 | 0.00 | -5.10 | 0.00 | -5.00 | 0.00 |
| ALPHA AMYL CINNAMALDEHYDE | **-6.08** | 0.26 | -4.98 | 0.02 | -5.30 | 0.00 | -4.63 | 0.17 |
| ALPHA AMYLCINNAMYL ALCOHOL | **-6.20** | 0.07 | -5.25 | 0.03 | -5.20 | 0.04 | -4.53 | 0.02 |
| ALPHA DAMASCONE | -5.80 | 0.00 | -5.25 | 0.05 | -5.05 | 0.03 | -4.80 | 0.00 |
| ALPHA HEXYL CINNAMALDEHYDE | **-6.10** | 0.33 | -4.85 | 0.12 | -5.03 | 0.14 | -4.78 | 0.17 |
| ALPHA ISOMETHYLIONONE | -5.58 | 0.18 | -5.00 | 0.06 | -4.78 | 0.11 | -4.43 | 0.08 |
| ALPHA PINENE | -5.10 | 0.00 | -5.10 | 0.00 | -4.00 | 0.00 | -4.23 | 0.02 |
| ALPHA TERPINENE | -5.75 | 0.03 | -4.80 | 0.00 | -5.18 | 0.03 | -4.40 | 0.00 |
| ALPHA TERPINEOL | -5.33 | 0.03 | -5.18 | 0.03 | -4.75 | 0.03 | -4.35 | 0.03 |
| AMYL SALICYLATE | **-6.10** | 0.07 | -4.83 | 0.03 | -5.15 | 0.09 | -4.95 | 0.03 |
| ANETHOLE | -5.95 | 0.03 | -4.78 | 0.02 | -4.68 | 0.23 | -4.18 | 0.03 |
| ANISE ALCOHOL | -5.23 | 0.14 | -4.68 | 0.03 | -4.33 | 0.13 | -4.18 | 0.03 |
| BENZALDEHYDE | -4.80 | 0.00 | -4.08 | 0.02 | -4.33 | 0.03 | -4.15 | 0.09 |
| BENZYL ALCOHOL | -4.73 | 0.05 | -4.23 | 0.08 | -4.25 | 0.05 | -4.00 | 0.10 |
| **BENZYL BENZOATE** | **-6.98** | 0.02 | **-6.15** | 0.26 | -5.93 | 0.08 | -5.73 | 0.18 |
| **BENZYL CINNAMATE** | **-7.30** | 0.04 | **-6.40** | 0.14 | -5.90 | 0.10 | **-6.15** | 0.10 |
| **BENZYL SALICYLATE** | **-7.20** | 0.00 | **-6.28** | 0.06 | -5.93 | 0.08 | **-6.13** | 0.05 |
| **BETA CARYOPHYLLENE** | **-6.60** | 0.00 | -5.68 | 0.03 | -5.90 | 0.00 | -5.00 | 0.00 |
| BETA DAMASCENONE | -5.58 | 0.02 | -5.80 | 0.00 | -5.30 | 0.00 | -5.20 | 0.00 |
| BETA DAMASCONE | -5.70 | 0.00 | -5.80 | 0.00 | -5.23 | 0.02 | -5.15 | 0.03 |
| BETA PINENE | -4.90 | 0.00 | -5.20 | 0.00 | -3.93 | 0.03 | -4.10 | 0.00 |
| CAMPHOR | -4.60 | 0.00 | -5.15 | 0.03 | -3.48 | 0.06 | -4.20 | 0.00 |
| CARVONE | **-6.00** | 0.00 | -5.10 | 0.00 | -5.05 | 0.05 | -4.50 | 0.00 |
| CINNAMALDEHYDE | -5.30 | 0.00 | -4.58 | 0.02 | -4.73 | 0.05 | -4.15 | 0.05 |
| CINNAMYL ALCOHOL | -5.18 | 0.19 | -4.58 | 0.13 | -4.68 | 0.13 | -4.30 | 0.07 |
| CITRAL | -5.40 | 0.04 | -4.53 | 0.02 | -4.55 | 0.12 | -4.15 | 0.03 |
| CITRONELLOL | -5.10 | 0.06 | -4.68 | 0.09 | -4.33 | 0.03 | -3.80 | 0.12 |
| COUMARIN | **-6.30** | 0.00 | -5.10 | 0.00 | -5.23 | 0.02 | -4.80 | 0.00 |
| DELTA DAMASCONE | -5.70 | 0.00 | -5.60 | 0.00 | -5.10 | 0.00 | -5.25 | 0.05 |
| **DIHYDROAMBRETTOLIDE** | **-6.20** | 0.00 | **-6.50** | 0.00 | **-6.03** | 0.02 | -5.28 | 0.02 |
| DIMETHYLBENZYLCARBINYL ACETATE | **-6.00** | 0.00 | **-6.20** | 0.00 | -5.03 | 0.06 | -4.63 | 0.05 |
| EBANOL | **-6.00** | 0.00 | **-6.23** | 0.02 | -5.08 | 0.05 | -4.73 | 0.02 |
| EUGENOL | -5.80 | 0.00 | -4.90 | 0.00 | -4.80 | 0.00 | -4.53 | 0.02 |
| EUGENYL ACETATE | **-6.10** | 0.00 | -5.30 | 0.00 | -5.00 | 0.00 | -4.83 | 0.03 |
| FARNESOL | -5.88 | 0.46 | -4.93 | 0.23 | -4.83 | 0.14 | -4.20 | 0.11 |
| **GALAXOLIDE** | **-6.95** | 0.03 | **-6.60** | 0.00 | -5.98 | 0.02 | -5.55 | 0.15 |
| GERANIOL | -5.33 | 0.05 | -4.65 | 0.22 | -4.63 | 0.03 | -4.05 | 0.09 |
| GERANYL ACETATE | -5.85 | 0.03 | -5.20 | 0.06 | -4.75 | 0.05 | -4.50 | 0.00 |
| HYDROXYCITRONELLAL | -5.33 | 0.33 | -4.95 | 0.06 | -4.23 | 0.23 | -4.18 | 0.31 |
| ISO E SUPER | -5.88 | 0.03 | **-6.23** | 0.02 | -5.25 | 0.03 | -5.10 | 0.00 |
| ISOEUGENOL | -5.75 | 0.25 | -5.10 | 0.17 | -4.90 | 0.10 | -4.55 | 0.03 |
| LILIAL (BUTYLPHENYL METHYLPROPIONAL) | -5.90 | 0.57 | -5.48 | 0.13 | -5.35 | 0.05 | -4.63 | 0.13 |
| LIMONENE | -5.60 | 0.00 | -4.70 | 0.00 | -4.80 | 0.00 | -4.30 | 0.00 |
| LINALOOL | -4.95 | 0.13 | -4.53 | 0.14 | -4.33 | 0.08 | -4.40 | 0.14 |
| LINALYL ACETATE | -5.45 | 0.05 | -5.10 | 0.00 | -4.68 | 0.05 | -4.83 | 0.08 |
| **LYRAL** | **-6.79** | 0.09 | **-6.73** | 0.02 | -5.43 | 0.28 | -5.05 | 0.03 |
| MAJANTOL | **-6.03** | 0.02 | -5.05 | 0.03 | -5.08 | 0.02 | -4.68 | 0.03 |
| MENTHOL | -5.80 | 0.00 | -4.70 | 0.00 | -4.88 | 0.03 | -4.58 | 0.02 |
| METHL-2-OCTYNOATE | -5.13 | 0.23 | -4.23 | 0.06 | -4.05 | 0.05 | -3.80 | 0.04 |
| METHYL SALICYLATE | -5.63 | 0.03 | -5.03 | 0.02 | -4.70 | 0.00 | -4.53 | 0.02 |
| SALICYLALDEHYDE | -5.15 | 0.03 | -4.50 | 0.00 | -4.30 | 0.00 | -4.33 | 0.03 |
| SANTALOL | -5.75 | 0.06 | -5.83 | 0.14 | -5.23 | 0.02 | -4.58 | 0.12 |
| **SCLAREOL** | -5.83 | 0.09 | **-6.50** | 0.00 | -5.30 | 0.16 | -5.80 | 0.00 |
| TERPINOLENE | -5.90 | 0.00 | -4.80 | 0.00 | -4.70 | 0.00 | -4.50 | 0.00 |
| VANILLIN | -5.40 | 0.00 | -4.88 | 0.03 | -4.50 | 0.00 | -4.88 | 0.03 |
| VERTOFIX (ALPHA ACETYL CEDRENE) | **-6.30** | 0.00 | **-6.63** | 0.03 | -5.13 | 0.03 | -5.20 | 0.00 |
| **Artemisinin** | **-6.98** | 0.02 | **-7.20** | 0.00 | **-6.03** | 0.02 | **-6.05** | 0.03 |
| **Favipiravir** | -5.50 | 0.00 | -4.70 | 0.00 | -4.00 | 0.00 | -4.43 | 0.02 |
| **Hydroxychloroquine** | **-6.18** | 0.15 | -5.83 | 0.14 | -4.80 | 0.14 | -4.50 | 0.08 |
| **Nigellidine** | **-8.20** | 0.00 | **-7.40** | 0.00 | -5.45 | 0.06 | -5.88 | 0.03 |
| **Remdesivir** | **-6.90** | 0.08 | **-6.65** | 0.13 | -5.95 | 0.29 | **-6.95** | 0.13 |

**Table S2:** Reranked scores from Molegro Virtual Docker. Top nine molecules from Vina results and previously reported compounds printed in bold letters.

|  | BRD2 | Main Protease | Nucleocapsid | Receptor Binding Domain |
| --- | --- | --- | --- | --- |
| **3-PROPYLIDENE PHTHALIDE** | -46.80 | -47.72 | -42.92 | -53.55 |
| ACETYL ISOEUGENOL | -55.28 | -63.85 | -15.72 | -54.26 |
| ALPHA AMYL CINNAMALDEHYDE | -61.94 | -70.20 | -50.68 | -65.18 |
| ALPHA AMYLCINNAMYL ALCOHOL | -59.09 | -73.69 | -60.25 | -42.07 |
| ALPHA DAMASCONE | -49.19 | -61.78 | -43.18 | -49.09 |
| ALPHA HEXYL CINNAMALDEHYDE | -60.74 | -77.78 | -61.62 | -61.11 |
| ALPHA ISOMETHYLIONONE | -43.65 | -54.98 | -22.34 | -42.55 |
| ALPHA PINENE | -32.69 | -49.66 | -27.11 | -36.65 |
| ALPHA TERPINENE | -42.96 | -54.79 | -34.60 | -44.95 |
| ALPHA TERPINEOL | -50.03 | -56.77 | -41.78 | -42.07 |
| AMYL SALICYLATE | -59.78 | -81.63 | -42.17 | -60.79 |
| ANETHOLE | -49.89 | -49.86 | -55.41 | -46.65 |
| ANISE ALCOHOL | -41.78 | -50.33 | -53.67 | -43.09 |
| BENZYL ALCOHOL | -35.02 | -45.54 | -45.48 | -36.59 |
| **BENZYL BENZOATE** | -58.28 | -70.43 | -75.74 | -48.56 |
| **BENZYL CINNAMATE** | -66.03 | -70.12 | -68.95 | -49.24 |
| **BENZYL SALICYLATE** | -52.66 | -76.54 | -65.86 | -60.78 |
| **BETA CARYOPHYLLENE** | -17.83 | -49.10 | 66.48 | -51.20 |
| BETA DAMASCENONE | -57.01 | -49.79 | -43.56 | -56.51 |
| BETA DAMASCONE | -54.18 | -51.68 | -41.65 | -50.02 |
| BETA PINENE | -29.62 | -47.82 | -27.00 | -35.30 |
| CAMPHOR | -15.60 | -60.16 | -13.15 | -34.24 |
| CARVONE | -46.78 | -56.60 | -51.28 | -48.83 |
| CINNAMALDEHYDE | -45.48 | -52.64 | -48.71 | -41.38 |
| CINNAMYL ALCOHOL | -44.92 | -55.74 | -49.01 | -33.53 |
| CITRAL | -50.15 | -54.75 | -56.65 | -47.66 |
| CITRONELLOL | -49.00 | -57.72 | -38.38 | -48.98 |
| COUMARIN | -37.97 | -52.33 | -51.28 | -36.50 |
| DELTA DAMASCONE | -41.74 | -46.16 | -44.61 | -55.09 |
| **DIHYDROAMBRETTOLIDE** | -28.82 | -49.49 | -48.30 | -58.99 |
| DIMETHYLBENZYLCARBINYL ACETATE | -48.94 | -60.93 | -35.93 | -50.46 |
| EBANOL | -55.15 | -58.66 | -44.04 | -55.18 |
| EUGENOL | -51.07 | -64.13 | -66.82 | -52.15 |
| EUGENYL ACETATE | -52.37 | -57.57 | -41.08 | -54.81 |
| FARNESOL | -63.59 | -88.32 | -62.75 | -50.89 |
| **GALAXOLIDE** | -45.93 | -64.23 | -7.08 | -56.72 |
| GERANIOL | -52.34 | -59.89 | -58.16 | -54.21 |
| GERANYL ACETATE | -58.23 | -75.64 | -57.13 | -54.44 |
| HYDROXYCITRONELLAL | -66.07 | -60.19 | -61.01 | -47.50 |
| ISO E SUPER | -41.89 | -40.88 | 2.33 | -44.72 |
| ISOEUGENOL | -47.78 | -70.12 | -68.40 | -53.89 |
| LILIAL (BUTYLPHENYL METHYLPROPIONAL) | -50.95 | -59.22 | -47.27 | -46.66 |
| LIMONENE | -40.21 | -45.37 | -39.08 | -47.11 |
| LINALOOL | -44.57 | -54.75 | -49.28 | -42.29 |
| LINALYL ACETATE | -51.61 | -61.89 | -42.43 | -51.35 |
| **LYRAL (HYDROXYISOHEXYL-3-CYCLOHEXENE CARBOXALDEHYDE)** | -67.75 | -62.46 | -40.17 | -47.32 |
| MAJANTOL | -42.97 | -62.09 | -37.85 | -46.70 |
| MENTHOL | -44.18 | -50.87 | -13.54 | -46.98 |
| METHL-2-OCTYNOATE | -51.54 | -64.29 | -60.74 | -51.83 |
| METHYL SALICYLATE | -44.47 | -64.58 | -37.12 | -48.89 |
| SALICYLALDEHYDE | -38.55 | -55.48 | -34.42 | -42.89 |
| SANTALOL | -54.84 | -65.81 | -49.04 | -53.84 |
| **SCLAREOL** | -34.02 | -74.88 | 41.09 | -59.10 |
| TERPINOLENE | -40.80 | -50.49 | -24.96 | -46.65 |
| VANILLIN | -44.27 | -66.26 | -46.84 | -48.40 |
| VERTOFIX (ALPHA ACETYL CEDRENE) | -47.27 | -49.31 | -22.09 | -56.30 |
| **Artemisinin** | -54.01 | -17.63 | -5.73 | -29.13 |
| **Favipiravir** | -57.67 | -67.64 | -73.96 | -50.66 |
| **Hydroxychloroquine** | -74.13 | -92.39 | -73.65 | -55.37 |
| **Nigellidine** | -72.07 | -99.17 | -77.09 | -61.11 |
| **Remdesivir** | -118.45 | -137.03 | -54.14 | -95.58 |
